# Efficient generation of human cDC1-like cells to detect and enhance tumor-reactive T cells via CD4^+^ T cell help

**DOI:** 10.1016/j.crmeth.2026.101533

**Published:** 2026-07-22

**Authors:** Yizhi Wang, Xin Lei, Tom de Wit, Ellen Schrama, Marij J.P. Welters, Karel van Leeuwen, Sander de Kivit, Miriam de Boer, Jitske van den Bulk, Noel F.C. C. de Miranda, Ton N. Schumacher, Chong Sun, Jannie Borst, Yanling Xiao

**Affiliations:** 1 Department of Immunology, Leiden University Medical Center, Leiden, the Netherlands; 2 Department of Medical Oncology, Leiden University Medical Center, Leiden, the Netherlands; 3 Department of Pathology, Leiden University Medical Center, Leiden, the Netherlands; 4 Department of Molecular Oncology and Immunology, the Netherlands Cancer Institute, Amsterdam, the Netherlands; 5 Immune Regulation in Cancer, German Cancer Research Center, Heidelberg, Germany; 6 Oncode Institute, Utrecht, the Netherlands

**Keywords:** cDC1-licensing, blood progenitor, cDC1-like cell generation, tumor antigen-specific CTL, adoptive cell therapy

## Abstract

Classical type 1 dendritic cells (cDC1s) are crucial to anti-tumor immunity by cross-presenting tumor antigens and priming cytotoxic CD8^+^ T lymphocytes (CTLs). Licensing via CD4^+^ T cell help endows cDC1s with enhanced cross-presentation and CTL-priming capacity, making them a promising tool to improve adoptive T cell therapies. However, the scarcity of primary human cDC1s limits their in-depth translational investigation and application. Here, we describe a method to efficiently generate DCs *in vitro* from CD34^+^c-KIT^+^ progenitors in non-mobilized peripheral blood. This DC population is enriched for cDC1-like cells that respond to CD4^+^ T cell help by upregulation of key molecules involved in antigen cross-presentation and T cell costimulation. Upon CD4^+^ T cell-mediated licensing, the progenitor-derived DC promotes tumor-specific CTL priming and facilitates detection of rare tumor antigen-specific CD8^+^ T cells in blood and tumor tissues. This DC culture platform incorporating CD4^+^ T cell help provides a scalable system for immunomonitoring and optimizing adoptive T cell therapies in cancer.

## Highlights

• Generation of cDC1-like cells from progenitors in non-mobilized human blood
• CD4^+^ T cell-mediated licensing of cDC1-like cells improves CTL priming
• Antigen-loaded, helped/licensed cDC1-like cells detect rare tumor-specific CD8^+^ T cells
• Antigen-loaded, helped/licensed cDC1-like cells improve effective CTL differentiation

## Motivation

Classical type 1 dendritic cells (cDC1s) are specialized in cross-presenting tumor antigens and relaying CD4^+^ T cell help to prime CD8^+^ T cells, yet their scarcity in peripheral blood limits their practical use. We have developed a method to generate cDC1-like cells from hematopoietic progenitors as present in non-mobilized adult human blood. Once helped/licensed by activated CD4^+^ T cells, tumor antigen-loaded helped cDC1-like cells can identify and expand rare tumor antigen-specific CD8^+^ T cells from blood and induce their differentiation into cytotoxic T lymphocytes. This cDC1/T cell co-culture platform thereby improves detection and isolation of tumor-specific CD8^+^ T cells to serve immunomonitoring and adoptive T cell therapies in cancer.

## Graphical abstract

**Figure:**
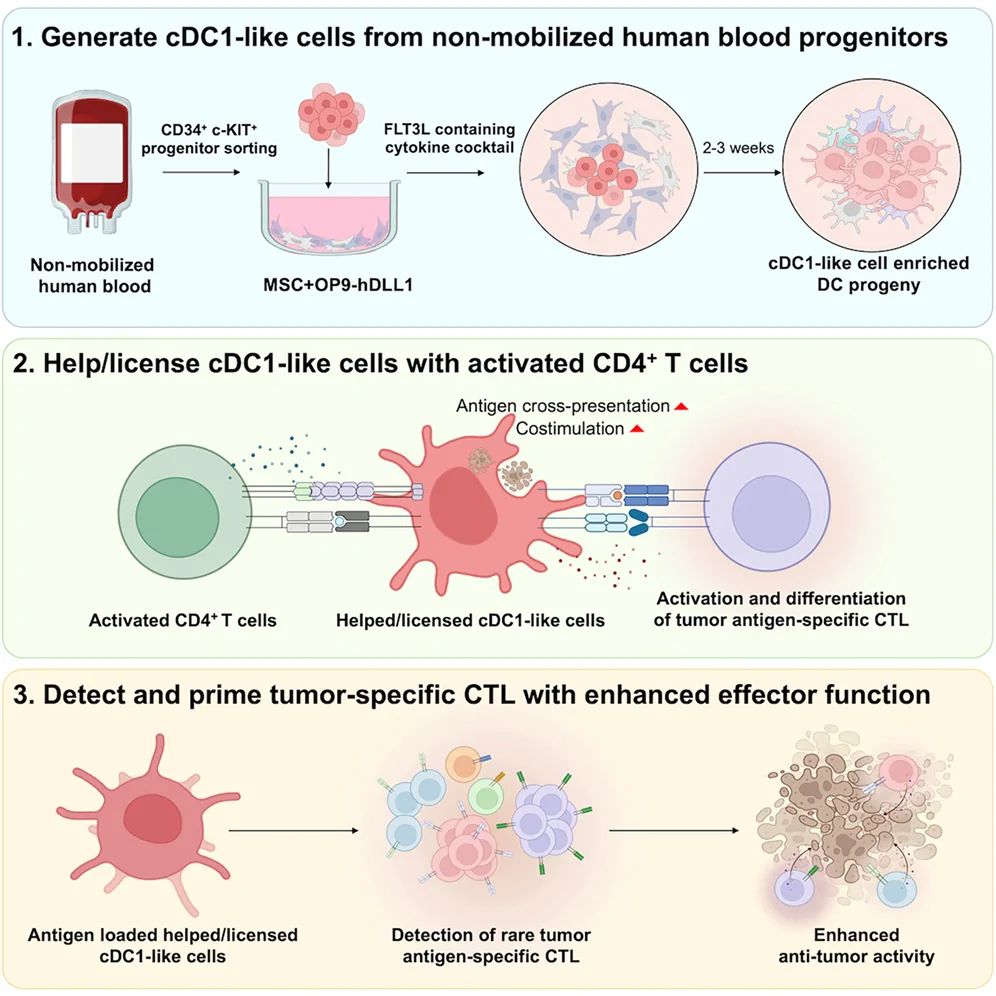

## Teaser

Wang et al. describe a methodology to generate cDC1-like cells from hematopoietic progenitors in non-mobilized human blood. These cDC1-like cells can be licensed by activated CD4^+^ T cells to detect rare tumor-specific CD8^+^ T cells in blood and prime functional tumor-reactive cytotoxic T cells. This platform supports immune monitoring and cell therapies in cancer.

## Introduction

Dendritic cells (DCs) are antigen-presenting cells that act as immune sentinels.^1^ They patrol tissues, phagocytose debris, and detect danger signals via pattern recognition receptors (PRRs).^2^ They migrate to lymph nodes (LNs) to induce either T cell tolerance or immunity. The DC lineage includes plasmacytoid DC (pDC) and classical DC (cDC) subsets,^3^^,^^4^ with cDCs further divided into cDC1 and cDC2^5^ that differ from monocyte-derived DCs (moDCs), which arise under inflammatory conditions.^3^^,^^6^^,^^7^ cDCs develop under homeostatic conditions in the bone marrow (BM) from hematopoietic progenitor cells (HPCs), driven by FLT3 receptor signaling.^8^ Subset development is regulated by transcription factor networks, ZEB2 for pDCs, IRF8 and BATF3 for cDC1s, and IRF4 for cDC2s and moDCs.^4^^,^^9^^,^^10^ DC subsets are also distinguished by key markers: CD123 and CD303 for pDCs, CD141 and XCR1 for cDC1s, CD1c and SIRPα for cDC2s, and CD14 and CD1a for moDCs.^4^^,^^11^

Among DC lineages, cDCs uniquely transport antigens from peripheral tissues to draining (d)LNs to initiate T cell activation.^12^^,^^13^ Specifically, cDC1s excel in cross-presenting cell-associated antigens on major histocompatibility complex (MHC) class I to activate CD8^+^ T cells.^14^ In tumors, cDC1s can maintain a reservoir of primed, stem-like CD8^+^ T cells in the tumor draining (td)LN^15^ and shape T cell states in the tumor microenvironment (TME).^16^^,^^17^^,^^18^^,^^19^ A key feature of cDC1s is their ability to interact with and respond to activated CD4^+^ T cells, a process known as DC licensing, which enhances their survival, maturation, migration, and antigen (cross)-presentation abilities.^19^^,^^20^ CD4^+^ T cell help promotes human cDC1 maturation and endows cDC1s with optimal cross-presentation ability, leading to superior priming of cytotoxic T lymphocytes (CTLs) against tumor-associated antigens as compared to cDC1s matured by PRR stimuli. The response to activated CD4^+^ T cells is specific for cDC1s and not displayed by primary cDC2s, pDCs, or moDCs.^19^ Activated CD4^+^ T cells license cDC1s via both CD40 ligand (L) and IFN-I,^20^^,^^21^^,^^22^ with the latter specifically promoting antigen cross-presentation features. cDC1 licensing by CD4^+^ T cells is required for optimal primary CTL responses to cancer, as shown by intervention studies in mouse models^20^^,^^23^ and by correlative evidence in humans.^19^^,^^22^ Also in infection, where PRR stimuli are abundant, cDC1 licensing by CD4^+^ T cells is required for CTL-based immunity.^24^ CD4^+^ T cell help promotes clonal expansion and effector and effector-memory differentiation of CD8^+^ T cells, making them highly functional CTLs.^25^^,^^26^ Through these mechanisms, cDC1s play a pivotal role in CTL-mediated anti-tumor immunity^27^^,^^28^^,^^29^ and are attractive targets or tools in cancer immunotherapy.

While immune checkpoint blockade, primarily programmed cell death protein 1 (PD-1) or programmed death-ligand 1 (PD-L1) inhibition, has achieved clinical success, its efficacy is largely limited to tumors already infiltrated by tumor-specific CTLs.^30^^,^^31^^,^^32^^,^^33^ For immunologically “cold” or non-immunogenic tumors, adoptive transfer therapy (adoptive cell therapy [ACT]) offers promise, via tumor-infiltrating lymphocytes (TILs)^34^ or genetically engineered T cells expressing tumor-specific receptors.^35^ Tumor-specific CD8^+^ T cells can now be identified using MHC class I multimers labeled with fluorophores^36^ or metals,^37^ phenotypic markers such as PD-1^38^ and CD39,^37^ functional assays measuring secretion of cytokines like IFN-γ,^39^ or through screening of TCRs.^40^^,^^41^ Although TILs are a rich source of tumor-reactive T cells, they are not readily available for all patients, and expanding them to clinically relevant numbers remains laborious and is not always successful. Circulating tumor-specific T cells offer an alternative, but their low frequency poses a major challenge.^42^^,^^43^^,^^44^ Additionally, resistance to ACT may result from impairment of T cell function or antigen recognition.^45^^,^^46^^,^^47^ This highlights the need for improved methods to detect tumor-specific T cells in blood and to enhance the functionality of therapeutic T cell products. In this context, cDC1s hold strong potential, both as tools to identify rare tumor-specific T cells and to increase their number and improve their function *in vivo*.

However, clinical application of cDC1s is limited by their scarcity, constituting only ∼0.02% of peripheral blood cells^19^ and showing substantial donor variability. A promising alternative is to generate cDC1-like cells *in vitro* from HPCs, which in current protocols is achieved by combining FTL3 ligand with delta-like ligand 1 (DLL1) that induces Notch signaling and improves murine^48^ and human^49^ cDC1 differentiation from progenitors. As HPC sources, cord blood^49^ and mobilized peripheral blood^50^ are used, which may be impractical for routine clinical application.

Here, we present an optimized method to generate cDC1-like cells in high yield from CD34^+^c-KIT^+^ HPCs, as isolated from non-mobilized adult blood. The *in*-*vitro*-generated progenitor-derived DC population is highly enriched for cDC1-like cells that respond to CD4^+^ T cell help as do primary cDC1s. The helped/licensed progenitor-derived DC population can improve effector responses of tumor-specific CTLs and enable sensitive detection of rare tumor antigen-specific CD8^+^ T cells in healthy human blood. We hereby offer a new methodology to improve personalized CTL-based immunotherapy.

## Results

### *In vitro* generation of human cDC1-like cells from adult blood-derived progenitors

**Figure 1:**
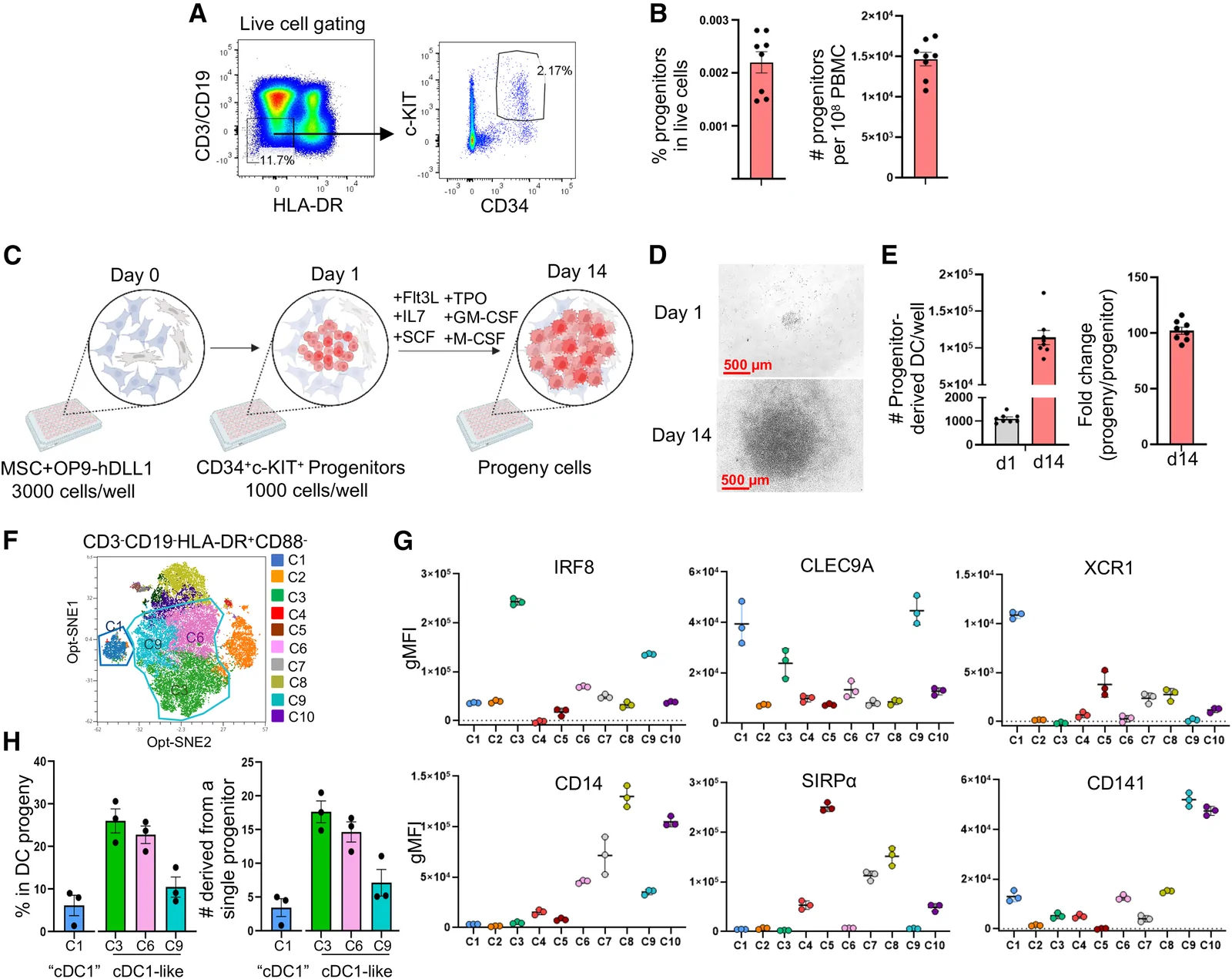
Generating human cDC1-like cells *in vitro* from progenitors in non-mobilized blood of healthy adult donors CD34^+^c-KIT^+^ progenitors were flow cytometrically sorted from PBMCs and co-cultured for 14–21 days at ∼1,000 cells per well with −3,000 feeder cells (70% hMSC + 30% hDLL1^+^OP9 cells) in the presence of FLT3L (100 ng/mL), TPO/SCF/GM-CSF (each 5 ng/mL), IL-7 (1 ng/mL), and M-CSF (0.5 ng/mL). (A) Gating strategy for progenitor cell isolation from PBMCs. (B) Frequency (%) of progenitors among live PBMCs and absolute numbers (#) of progenitors isolated from 100 × 10^6^ PBMCs. (C) Illustration of the culture system for *in vitro* DC differentiation from progenitors. (D) Microscopic pictures depicting outgrowth of cells from progenitors. (E) Absolute numbers (#) of progenitors on day 1 and progeny cells on day 14, and the fold change of progeny versus progenitor cells. (F) Opt-SNE plot of 10 clusters identified within CD3^−^CD19^−^HLA-DR^+^CD88^−^ progeny cells. Cluster 1 (C1) outlined in blue denotes cells with cDC1 phenotype, based on XCR1, CD141, CLEC9A, and IRF8. Combined C3, C6, and C9 outlined in light blue denote cells with cDC1-like phenotype. (G) gMFI quantifications for IRF8, CLEC9A, XCR1, CD14, SIRPα, and CD141 across the 10 progenitor-derived DC clusters. (H) Frequencies (%) of C1, C3, C6, and C9 in HLA-DR^+^CD88^−^ DC progeny and absolute numbers (#) of C1, C3, C6, and C9 calculated per input progenitor. Data are pooled from eight (*n* = 8) independent donors in (B) and (E); data are pooled from three independent donors (*n* = 3) in (G) and (H). Data are shown as means ± standard error of mean (SEM). See also Figure S2.

To facilitate implementation, we aimed to use non-mobilized adult blood as a source of CD34^+^cKIT ^+^ HPCs to generate cDC1s. Flow cytometric sorting (Figure 1A) yielded ∼15,000 progenitors per 10^8^ peripheral blood mononuclear cells (PBMCs) (Figure 1B). The progenitors failed to expand during the cytokine-driven phase when we used the protocol of Kirkling et al.^47^ for DC generation (method 1; Figures S1A and S1B). Directly culturing CD34^+^c-KIT ^+^ HPCs on different feeder layers (Figures S1A and S1B) with a cytokine cocktail of FLT3L, SCF, TPO, IL-7, GM-CSF, and M-CSF (FST7GM), based on Balan et al.^49^ and our previous work,^51^^,^^52^^,^^53^ yielded significantly more live cell progeny (methods 2 and 3; Figure S1C). As feeders, we first used OP9 cells of which 30% expressed human DLL1 (hDLL1) to engage Notch signaling and support cDC1 differentiation^49^ (method 2). We next used OP9-hDLL1 cells in combination with human BM-derived mesenchymal stromal cells (MSCs) to support HPC growth^51^^,^^52^^,^^53^^,^^54^^,^^55^ (method 3). This led to a 10-fold increase in live cell yield (Figure S1C) and a significantly higher frequency and absolute number of cells with general DC markers (CD11c^+^HLA-DR^+^) and cDC1 markers (XCR1 and CLEC9A) (Figures S1D–S1F). Using method 3, progenitors expanded ∼100-fold (Figures 1C–1E), yielding ∼60% DCs with a CD3^−^CD19^−^HLA-DR^+^CD88^−^ phenotype, wherein CD88 is a marker associated with moDCs^56^ (Figures S2A and S2B).

To assess the lineage composition of the progenitor-derived DC culture, we analyzed it by spectral flow cytometry for many relevant markers. The culture was clearly heterogeneous and resolved into ten clusters with discernible phenotypes (Figure 1F; Figure S2C). Both CD123 and CD303 were not detected, indicating that pDCs were absent among the progenitor-derived DCs (Figure S2D). Quantitative analysis of all markers by OMIQ identified clusters C1, C3, C6, and C9 as cDC1-like cells, on the basis of expression of CLEC9A and IRF8, as well as the lack of SIRPα (Figure 1G; Figure S2D). Notably, CD141, a classical marker of *ex vivo* cDC1,^4^^,^^11^ was highly expressed on cDC1-like cells in cluster 9 but also on the CD14^+^ monocytic cluster 10 (Figure 1G). Up to 60% of the population consisted of cDC1-like cells (Figure 1H; Figure S2C). The other clusters contained cells with cDC2 features and monocytic features (Figure 1G; Figures S2D and S2E). Manual gating also discerned CD14^−^CLEC9A^+^XCR1^+^ cDC1-like cells, CD14^−/+^IRF8^+^SIRPα^−^ cDC1-like cells, and CD14^+^IRF8^−^SIRPα^+^ cDC2-like/monocytic cells (Figure S2F). To assess the maturity of the cDC-like populations in our culture, we compared them to pre-DC phenotype cells in peripheral blood that express AXL, SIGLEG6, and FLT3 and low levels of CD45RA.^7^^,^^57^ Unlike *ex vivo* AXL^+^SIGLEC6^+^ cells in peripheral blood, progenitor-derived CLEC9A^+^ and IRF8^+^SIRPα^−^ cDC1-like cells as well as IRF8^−^SIRPα^+^ cDC2-like/monocytic cells lacked AXL and SIGLEC6 expression, while retaining FLT3 and exhibiting variable levels of CD45RA (Figures S2G–S2I). These data argue that the DC population obtained from the progenitors was of variable maturity, differentiated beyond the pre-DC state. Based on the total input of progenitors and final cDC1-like cell counts (C1, C3, C6, and C9), this corresponds to an average yield of approximately 45 cDC1-like cells per input progenitor (Figure 1H).

### Response of progenitor-derived cDC1-like cells to activated CD4^+^ T cells

We previously demonstrated that *ex vivo* human cDC1s are unique as compared to cDC2s, pDCs, and moDCs in responding to activated CD4^+^ T cells by upregulating multiple pathways important for antigen cross-presentation and CTL priming.^19^ In that *in vitro* setup, we mimicked cDC1 licensing by CD4^+^ T cells by activating CD4^+^ T cells with agonistic anti-CD3 monoclonal antibodies (mAb) instead of letting them recognize specific antigen, as occurs physiologically. We here tested, in the same way, whether the progenitor-derived DC population responded to activated CD4^+^ T cells by reading out the same costimulatory molecules, chemokines, and antigen-presenting molecules. Despite the heterogeneity of the gated CD3^−^HLA-DR^+^ population, various DC licensing markers^19^ were significantly upregulated in response to activated, but not naive, CD4^+^ T cells, including CD70, CD80, CD83, PD-L1, chemokines CXCL9/10, as well as MHC class I and II (Figures S3A–S3F). Comparison of the response of the progenitor-derived-DCs to activated CD4^+^ T cells versus PRR stimulation showed that stimulation with activated CD4^+^ T cells led to significantly higher expression of various costimulatory molecules and of CXCL9/10 (Figures S3H–S3K).

**Figure 2:**
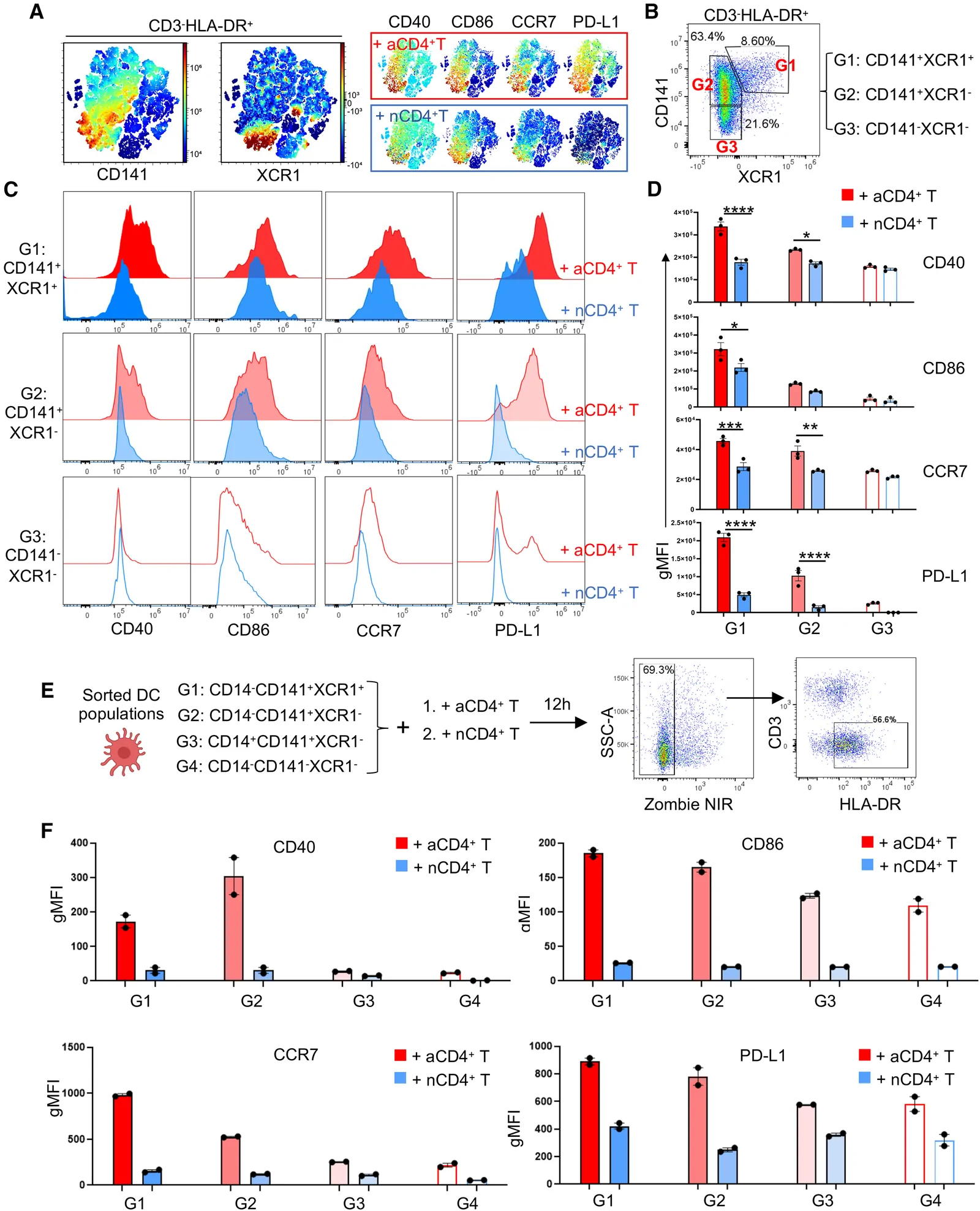
Response of progenitor-derived cDC1-like cells to activated CD4^+^ T cells Progenitor-derived DCs were co-cultured with activated (a) or naive (n) CD4^+^ T cells overnight. Next, key molecules of the cDC1 “help” signature^19^ were analyzed by flow cytometry. (A) Opt-SNE plots (left) showing clusters of progenitor-derived DCs incorporating both culture conditions (+nCD4^+^ T and + aCD4^+^ T), colored by CD141 or XCR1 expression. Opt-SNE plots (right) showing CD40, CD86, CCR7, and PD-L1 expression on progenitor-derived DCs after co-culture with either aCD4^+^ or nCD4^+^ T cells. (B) Gating strategy for defining 3 subpopulations within CD3^−^HLA-DR^+^ progenitor-derived DC population after co-culture with either aCD4^+^ or nCD4^+^ T cells, based on XCR1 and CD141 expression. (C) Representative histogram showing expression of the indicated markers in the 3 progenitor-derived DC subpopulations under indicated conditions. (D) Quantification of gMFI comparing expression levels of the indicated markers across the 3 progenitor-derived DC subpopulations after co-culture with aCD4^+^ or nCD4^+^ T cells. (E and F) Responses of sorted progenitor-derived DCs to activated upon CD4^+^ T cell. (E) Schematic illustration of the experimental design. The four sorted populations were cultured either alone or together with activated CD4^+^ T cells. (F) Quantification of gMFI of the indicated markers on the corresponding sorted progenitor-derived DC populations under the indicated conditions. Data in (D) are pooled from three independent donors (*n* = 3), and data in (F) are pooled from two independent donors (*n* = 2). Data are shown as means ± SEM in (D) and means ± standard deviation (SD) in (F). ∗*p* < 0.05, ∗∗*p* < 0.01, ∗∗∗*p* < 0.001, ∗∗∗∗*p* < 0.0001. Statistical analyses were performed using a two-way ANOVA with Tukey’s multiple comparisons for (D). See also Figures S4A and S4B.

To assess whether cDC1-like cells were the responders to the activated CD4^+^ T cells, we further zoomed in on the CD3^−^HLA-DR^+^ population tested in Figures S3A–S3F, analyzing separately the response of gated CD141^+^XCR1^+^, CD141^+^XCR1^−^, and CD141^−^XCR1^−^ cells (Figures 2A and 2B). The most pronounced upregulation of cDC1 licensing markers CD40, CD86, CCR7, and PD-L1 in response to stimulation by activated versus naive CD4^+^ T cells was observed in the CD141^+^XCR1^+^ population, followed to a lesser extent by the CD141^+^XCR1^−^ population, whereas the CD141^−^XCR1^−^ population showed minimal responses (Figures 2C and 2D). These results indicate that the response can primarily be attributed to the cDC1-like compartment within the heterogeneous DC population. To further substantiate these findings, we examined the response of flow cytometrically sorted subsets of the progenitor-derived DC population. Four test populations were sorted from CD11c^+^HLA-DR^+^ cells: CD14^−^CD141^+^XCR1^+^ (cDC1-like), CD14^−^CD141^+^XCR1^−^ (cDC1-like), CD14^+^CD141^+^XCR1^−^ (cDC2-like and moDC-like), and CD14^−^CD141^−^XCR1^−^ (undefined), which were stimulated with activated or naive CD4^+^ T cells (Figure 2E; Figure S4A). Consistent with the gating-based analysis, the cDC1-like fractions showed the most pronounced and consistent upregulation of cDC1 licensing markers CD40, CD86, CCR7, and PD-L1 (Figure 2F; Figure S4B). These results indicate that cDC1-like cells are the dominant responders to CD4^+^ T cell help, while other myeloid populations may contribute, but to a lesser extent.

**Figure 3:**
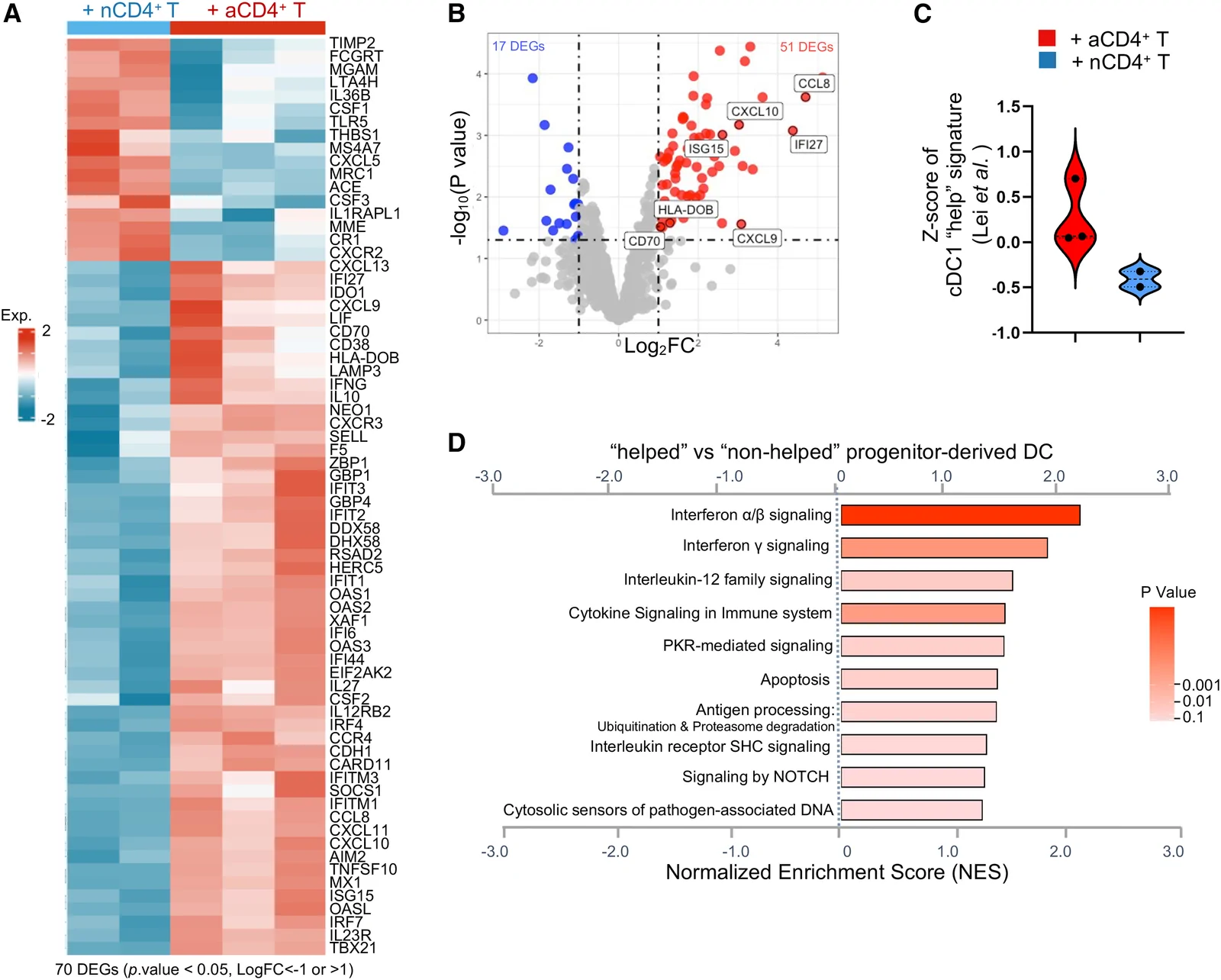
Gene profiling by NanoString reveals defined licensing signature in progenitor-derived DCs Bulk progenitor-derived DCs were co-cultured with either activated (a) or naive (n) CD4^+^ T cells. Subsequently, HLA-DR^+^CD3^−^ cells were sorted, lysed, and subjected to NanoString nCounter analysis. (A) Heatmap displaying normalized and log_2_-transformed transcript levels of differentially expressed genes (DEGs) (*p* < 0.05) across progenitor-derived DC samples co-cultured with either aCD4^+^ T cells (*n* = 3) or nCD4^+^ T cells (*n* = 2). (B) Volcano plot depicting DEGs between progenitor-derived DCs co-cultured with aCD4^+^ or nCD4^+^ T cells. Genes shown in red or blue have *p* < 0.05 and log_2_ fold change (log_2_FC) > 1 in the respective conditions. (C) *Z* scores were calculated per sample by aggregating expression values of genes from a previously reported cDC1 “help” signature,^19^ using the average of scaled (z-transformed) expression values. Violin plots depict the distribution of *Z* scores between progenitor-derived DCs co-cultured with aCD4^+^ or nCD4^+^ T cells. Each dot represents one sample. (D) Bar chart showing the top ten enriched Reactome pathways among genes upregulated in “helped” progenitor-derived DCs. Pathways are ranked by normalized enrichment score (NES), and only positively enriched pathways (NES >0) are shown. See also Figures S4C and S4D.

Analysis of gene expression in the CD3^−^HLA-DR^+^ progenitor-derived DC population using the nCounter Host Immune Response Panel (Figure S4C; Table S1) revealed 70 differentially expressed genes (DEGs) between the two conditions (Figure S4C; Figure 3A; Table S2), including characteristic upregulation of CD70, CXCL9, and CXCL10^19^ in response to activated CD4^+^ T cells (Figure 3B). In fact, after culture with activated CD4^+^ T cells, progenitor-derived DCs showed enrichment of the “help” gene signature we previously defined in primary *ex vivo* cDC1s^19^ (Figure 3C; Tables S3 and S4), along with upregulation of IFN-α/β/γ and IL-12 cytokine signaling, as well as of the antigen presentation pathways (Figure 3D; Figure S4D). Collectively, these findings indicate that progenitor-derived DCs respond to activated CD4^+^ T cells, with the response primarily driven by the cDC1-like population.

### Progenitor-derived DCs can relay CD4^+^ T cell help for priming of (tumor) antigen-specific CTL

**Figure 4:**
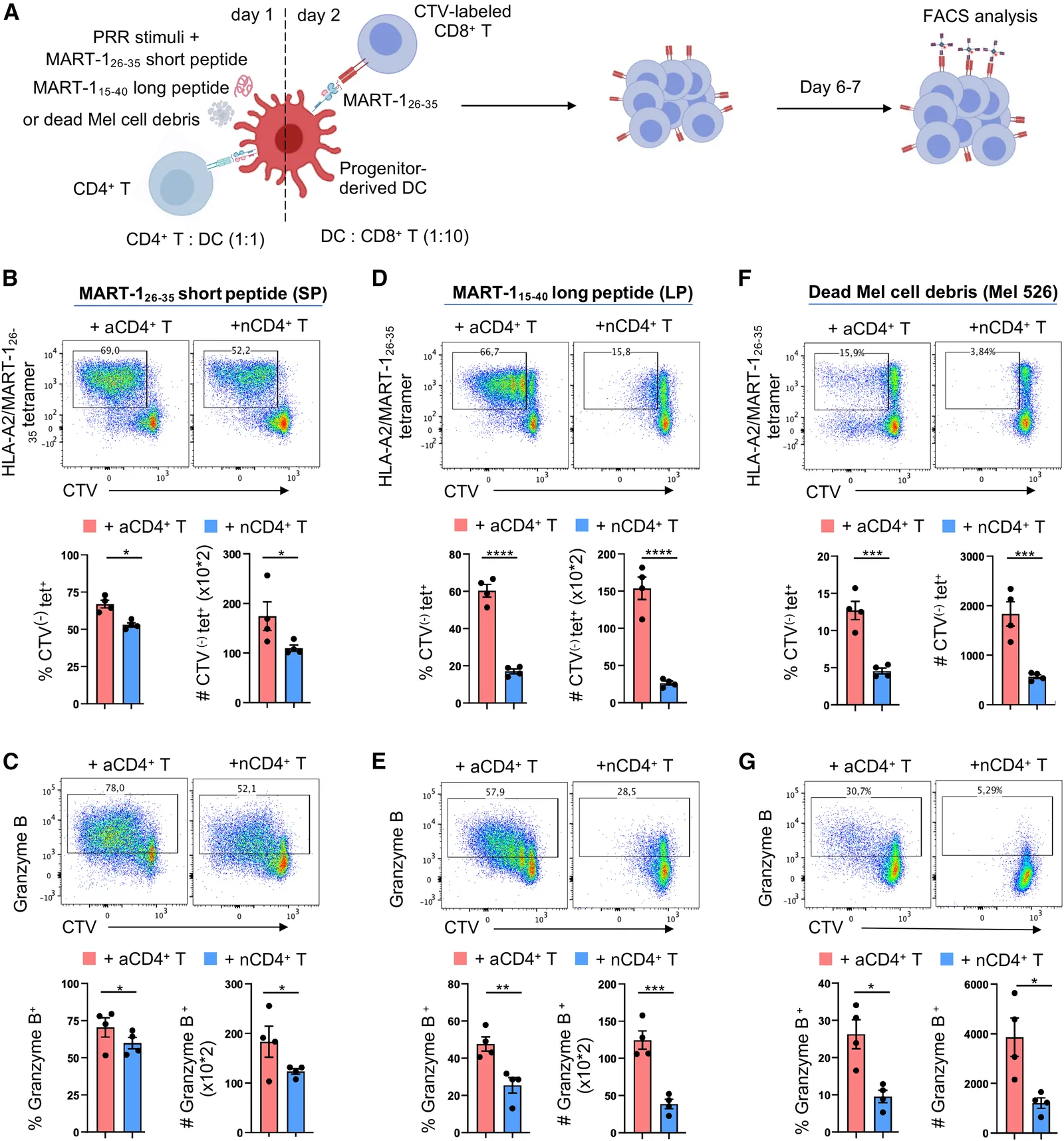
Progenitor-derived DCs have the ability to relay CD4^+^ T cell help for CTL responses (A) Scheme of the tumor antigen-specific CTL priming system. On day 1, ∼5,000 total progenitor-derived DCs from HLA-A2^+^ healthy donors were incubated with equal numbers of activated (a) or naive (n) CD4^+^ T cells, MART-1 peptide or MART-1^+^ melanoma cell debris, and a mixture of PRR stimuli. On day 2, ∼50,000 CD8^+^ T cells transduced to express the MART-1_26–35_/HLA-A2-specific TCR and having a stem cell-like memory T cell (T_SCM_) phenotype^19^ were added. The T cell response was readout at day 6 or 7 after co-culture. (B, D, and F) CD8^+^ T cell proliferation to (B) MART-1_26–35_ short peptide (SP), (D) MART-1_15–40_ long peptide (LP), or (F) dead Mel526 cell debris based on CTV dilution. Upper: primary flow cytometric data. Lower: frequency (%) of MART-1_26–35_/HLA-A2-specific (tet^+^) cells within CTV^(−)^ CD8^+^ T cells, and their absolute, live cell number (#). (C, E, and G) CTL response to (C) MART-1_26–35_ SP, (E) MART-1_15–40_ LP, or (G) dead Mel526 cell debris based on intracellular granzyme B staining. Upper: primary flow cytometric data. Lower: frequency of granzyme B^+^ cells among CD8^+^ T cells, and their absolute, live cell number. Data are pooled from four (*n* = 4) independent experiments and are shown as means ± SEM. ∗*p* < 0.05, ∗∗*p* < 0.01, ∗∗∗*p* < 0.001, ∗∗∗∗*p* < 0.0001 (two-sided unpaired Student’s *t* test). See also Figure S5.

CD4^+^ T cell help endows cDC1s with the optimal capacity to cross-prime CTLs in response to tumor cell debris.^19^^,^^24^ We used our *in vitro* CTL-priming platform^19^ to investigate whether progenitor-derived DCs could relay CD4^+^ T cell help for CTL (cross)-priming. CD8^+^ T cells from PBMCs were transduced with a TCR specific for the MART-1_26–35_ peptide presented by HLA-A2. HLA-A2^+^ HPCs were used to generate progenitor-derived DCs, which were cultured with naive or activated CD4^+^ T cells, loaded with the MART-1_26–35_ short peptide (SP), MART-1_15–40_ long peptide (LP), or debris of MART-1-expressing Mel526 cells^19^ in the presence of PRR stimuli, and then co-cultured with CellTrace Violet (CTV)-labeled CD8^+^ T cells (Figure 4A). While SP can be loaded into MHC-I directly, LP and particularly cell debris require uptake and processing for cross-presentation. The CD8^+^ T cell response was read out by CTV dilution and granzyme B production (Figure S5A). In the SP setting, progenitor-derived DCs induced robust MART-1-specific CD8^+^ T cell proliferation (Figure 4B) and granzyme B production (Figure 4C), with a modest effect of activated CD4^+^ T cells (Figures 4B and 4C). In the LP setting, CTL responses were significantly higher when DCs were “helped” by activated CD4^+^ T cells (Figures 4D and 4E). In the Mel526 debris setting, progenitor-derived DCs phagocytosed GFP^+^ melanoma cell debris, as shown by GFP, CD45, and CD11c co-expression via imaging flow cytometry (Figures S5B–S5D). Only helped DCs induced MART-1_26–35_-specific CD8^+^ T cell proliferation (Figure 4F) and granzyme B production (Figure 4G), whereas “non-helped” DCs failed to do so (Figures 4F and 4G). Interestingly, proliferation was also observed in HLA-A2/MART-1_26–35_ tetramer-negative CD8^+^ T cells, indicating that progenitor-derived DCs can prime CTLs against tumor antigens beyond MART-1 (Figure 4F), as demonstrated in our previous study.^19^ Thus, similar to *ex vivo* cDC1s,^19^ progenitor-derived DCs responded to CD4^+^ T cell help by enhancing their CTL (cross)-priming capacity.

We also tested whether progenitor-derived DCs could prime naive CD8^+^ T cells that recognized alloantigen with their endogenous TCR. For this purpose, naive CD3^+^CD8^+^CD45RA^+^CCR7^+^ CD8^+^ T cells and CD3^+^CD4^+^CD45RA^+^CD25^−^ CD4^+^ T cells were isolated by flow cytometry from the same donors, while progenitor-derived DCs were derived from different, HLA-mismatched donors (Figure S6A). CD4^+^ T cells were activated with anti-CD3 mAb or not and co-cultured overnight, as in previous assays, with progenitor-derived DCs. Next, CTV-labeled naive CD8^+^ T cells were added and co-cultured with the HLA-mismatched progenitor-derived DCs for 6 days, after which CD8^+^ T cell proliferation was quantified based on CTV dilution (Figure S6B). Progenitor-derived DCs that had been co-cultured with naive CD4^+^ T cells could induce a response in naive CD8^+^ T cells as compared to the setting without DCs, but the response was greatly enhanced by activated CD4^+^ T cell help (Figures S6C and S6D). These data provide evidence that progenitor-derived DCs can prime naive CD8^+^ T cells that recognize alloantigen and that this capacity is improved upon their licensing by activated CD4^+^ T cells.

### Detection of tumor-specific CD8^+^ T cells in healthy donors with helped progenitor-derived DCs

**Figure 5:**
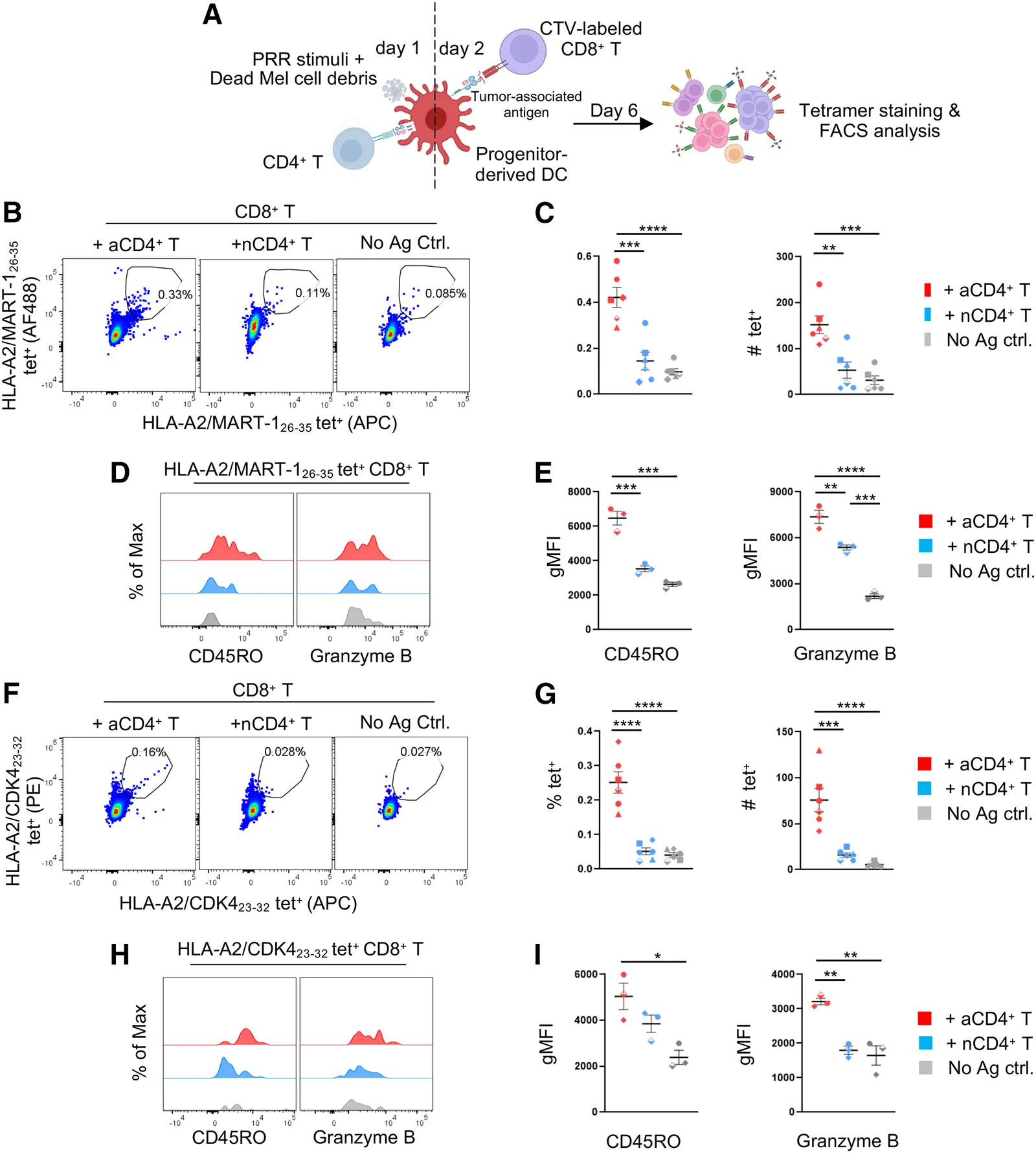
Detecting tumor antigen-specific CD8^+^ T cells in healthy donor blood with “helped” progenitor-derived DC Per assay, ∼50,000 CD8^+^ T cells sorted from PBMCs were co-cultured with ∼5,000 progenitor-derived DCs loaded with dead Mel526 cell debris in the presence of ∼5,000 activated (a)- or naive (n)CD4^+^ T cells. Tumor antigen-reactive CD8^+^ T cells were detected using peptide/MHC-I tetramers and T cell activation markers. (A) Experimental design. (B) Flow cytometric plots depicting HLA-A2/MART-1_26–35_ tetramer (tet) staining of CD8^+^ T cells after culture. (C) Frequency (%) of and total number (#) of MART-1_26–35_ tet^+^ CD8^+^ T cells within total CD8^+^ T cells. (D and E) Histograms (D) and gMFI quantifications (E) of CD45RO and granzyme B expression by MART-1_26–35_ tet^+^ CD8^+^ T cells under indicated conditions. (F) Flow cytometric plots depicting HLA-A2/CDK4_23–32_ tet staining of CD8^+^ T cells after culture. (G) Frequency and absolute number (#) of CDK4_23–32_ tet^+^ cells within total CD8^+^ T cells. (H and I) Histogram (H) and gMFI quantifications (I) of CD45RO and granzyme B expression by CDK4_23–32_ tet^+^ CD8^+^ T cells under indicated conditions. Data are pooled from six (*n* = 6 in C and G) and three (*n* = 3 in E and I) independent donors and are shown as means ± SEM. ∗*p* < 0.05, ∗∗*p* < 0.01, ∗∗∗*p* < 0.001, ∗∗∗∗*p* < 0.0001. Statistical analyses were performed using one-way ANOVA with Tukey’s multiple comparisons for (C), (E), (G), and (I). See also Figures S7A–S7D.

We next tested whether our CTL-priming platform with progenitor-derived DCs could be used to detect tumor antigen-specific CD8^+^ T cells in healthy donor PBMCs. As proof of principle, we tested reactivity against three HLA-A2-restricted melanoma antigens (MART-1/CDK4/MAGE3) (Figure S7A), using debris from Mel526 cells expressing these antigens as the source. DCs and T cells from the same HLA-A2^+^ donors were co-cultured for 6 days, after which tumor antigen-specific CD8^+^ T cells were detected with peptide/MHC-I tetramers, in combination with activation markers (Figure 5A; Figure S7A). Tetramers conjugated to two fluorochromes improved detection specificity (Figures 5B and 5F). In the help setting with activated CD4^+^ T cells, MART-1_26–53_-specific CD8^+^ T cells were significantly amplified to 0.42% ± 0.04% of total CD8^+^ T cells, versus 0.14% ± 0.03% in the “no help” setting with naive CD4^+^ T cells, which was similar to the frequency in the no antigen control (Figures 5B and 5C). Likewise, the frequency of HLA-A2/CDK4_23–32_ tet^+^CD8^+^ T cells was increased to 0.25% ± 0.03% in the help setting, versus 0.05% ± 0.01% in the no help setting (Figures 5F and 5G). Absolute numbers of tetramer^+^CD8^+^ T cells were also increased with CD4^+^ T cell help (Figures 5C and G). Moreover, MART-1_26–53_- and CDK4_23–32_-specific CD8^+^ T cells in the help setting showed enhanced effector differentiation with increased expression levels of CD45RO and granzyme B (Figures 5D, 5E, 5H, and 5I). In the help setting, CD8^+^ T cells specific for HLA-A2/MAGE3_271–279_ were likewise amplified and thereby more easily detected (Figures S7B–S7D). We conclude that our platform of *in vitro* help delivery to CD8^+^ T cells with progenitor-derived DCs amplifies tumor-specific CD8^+^ T cells and thereby facilitates their detection among T cells present in the blood of adult healthy donors.

**Figure 6:**
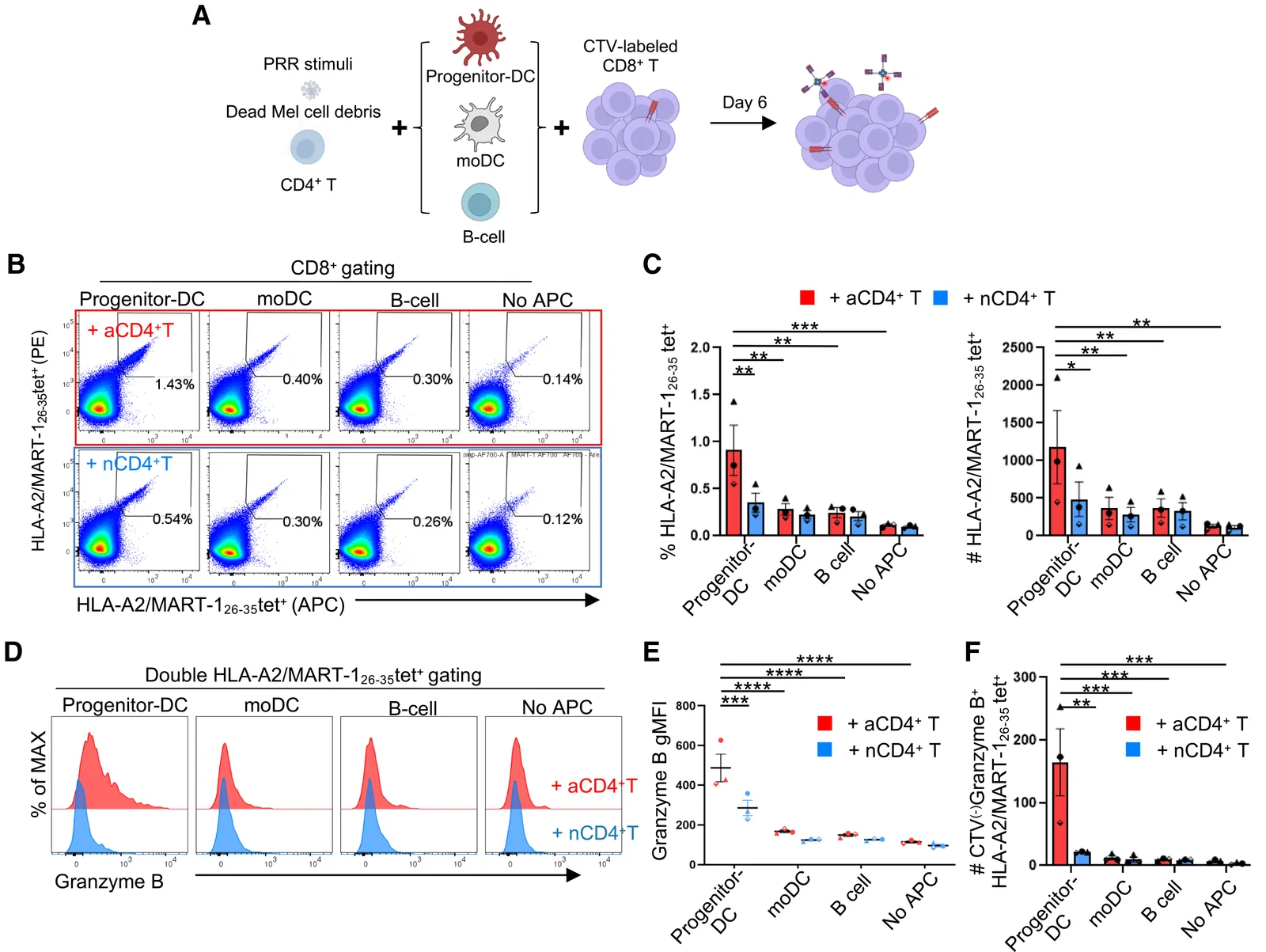
Helped progenitor-derived DCs efficiently detect tumor antigen-specific CD8^+^ T cells in blood and outperform moDCs and B cells Progenitor-derived DCs, moDCs, and B cells from matched donors were compared for their ability to detect and induce activation of endogenous MART_26–35_-specific CD8^+^ T cells from blood. (A) Schematic of the experimental design. On day 1, ∼100,000 total progenitor-derived DCs, moDCs, or B cells from HLA-A2^+^ healthy donors were incubated with equal numbers of activated (a) or naive (n) CD4^+^ T cells, Mel526 cell debris, and a mixture of PRR stimuli. On day 2, ∼1,000,000 polyclonal CD8^+^ T cells were added. The T cell response was readout at day 6 after co-culture. (B) Representative flow cytometric plots showing double staining with MART-1_26–35_/HLA-A2 tetramers for indicated conditions. (C) Frequency (%) of MART-1_26–35_/HLA-A2 tetramer-positive cells among CD8^+^ T cells, and their absolute number (#). (D) Representative histogram showing granzyme B expression CD8^+^ T cells under the indicated conditions. (E) Quantification of gMFI for granzyme B under the indicated conditions. (F) Absolute number (#) of CTV^(−)^granzyme B^+^ MART-1_26–35_/HLA-A2 tetramer^+^ CD8^+^ T cells under the indicated conditions. Data are pooled from three (*n* = 3) healthy donors in (D), (F), and (G) and are shown as means ± SEM. ∗*p* < 0.05, ∗∗*p* < 0.01, ∗∗∗*p* < 0.001, ∗∗∗∗*p* < 0.0001 by one-way ANOVA with Tukey’s multiple comparisons. See also Figures S7E and S7F.

We next compared side-by-side the ability of progenitor-derived DCs and two commonly used antigen-presenting cell (APC) types, namely moDCs^58^ and B cells,^59^ to detect tumor antigen-specific CD8^+^ T cells. For this purpose, we used matched APCs from the same donors (*n* = 3) in the same experiment, reading out enrichment of detected MART-1_26–35_-specific CD8^+^ T cells after stimulation with the different APCs that had been stimulated with activated or naive CD4^+^ T cells (Figure 6A; Figure S7E). Progenitor-derived DCs stimulated by activated CD4^+^ T cells induced a higher frequency and absolute number of MART-1_26–35_-specific CD8^+^ T cells compared with moDCs, B cells, or conditions without APCs (Figures 6B and 6C). In addition, the CD8^+^ T cells primed by progenitor-derived DCs differentiated more effectively into CTLs, as indicated by granzyme B expression, especially under CD4^+^ T cell help conditions (Figures 6D–6F; Figure S7F).

### Helped progenitor-derived DCs enhance priming of functional tumor-specific CTLs

**Figure 7:**
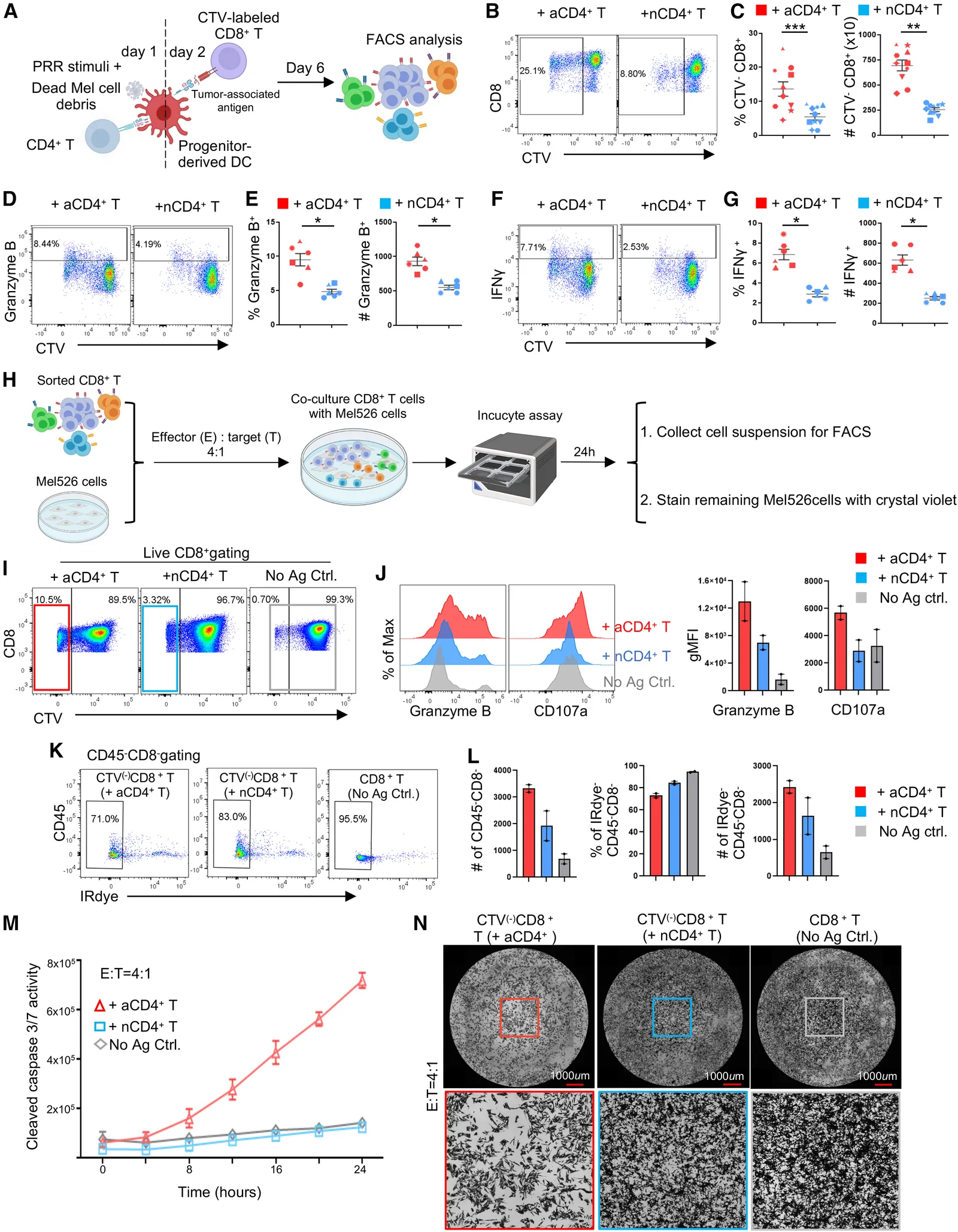
“Helped” progenitor-derived DCs enhance priming of functional tumor-specific CTLs (A–G) ∼50,000 CD8^+^ T cells were sorted from PBMCs and co-cultured with ∼5,000 progenitor-derived DCs loaded with dead Mel526 cell debris in the presence of ∼5,000 activated (a) or naive (n) CD4^+^ T cells for 6 days, after which the CD8^+^ T cell response was measured by flow cytometry. (A) Experimental design. (B) Flow cytometric plots depicting CD8^+^ T cell proliferation based on CTV dilution. (C) Frequency (%) and absolute number (#) of live CTV^(−)^ cells within total CD8^+^ T cells. (D and F) Flow cytometric plots depicting the CTL response based on intracellular granzyme B (D) and IFN-γ (F) staining. (E and G) Frequency (%) and absolute, live cell number (#) of granzyme B^+^ cells (E) and IFN-γ^+^ cells (G) within total CD8^+^ T cells. (H–N) Killing assay was performed using live proliferative (CTV negative) CD8^+^ T cells purified from the CTL priming system and Mel526 tumor cells at an effector/target (E/T) ratio of 4:1 and analyzed by IncuCyte during 24 h. At the end of the assay, the cell suspension was pooled and analyzed by flow cytometry. Remaining Mel526 cells in each well were fixed and stained with crystal violet. (H) Experimental design. (I) Gating strategy for isolating CTV^(−)^CD8^+^ T cells from the CTL priming system. Total live CD8^+^ T cells from no antigen setting were used as reference. (J) Histograms and gMFI quantifications for granzyme B and cell surface CD107α expression by CD8^+^ T cells after the killing assay. (K) Flow cytometric plots depicting total and detached Mel526 cells in the supernatant following the killing assay, as indicated by CD45^(−)^CD8^(−)^ and IRdye^(−)^CD45^(−)^CD8^(−)^ populations under indicated conditions. (L) Total number (#) of tumor cells, as well as frequency and number (#) of tumor cell loss measured in the supernatant. (M) Caspase-3/7 activity in tumor cells during the killing assay under the indicated conditions. (N) Representative CCD microscopic images depicting remaining Mel526 cells in the plate at 24 h post co-culture under indicated conditions. Data are pooled from ten (*n* = 10 in C) and six (*n* = 6 in E and G) independent donors and are shown as means ± SEM. ∗*p* < 0.05, ∗∗*p* < 0.01, ∗∗∗*p* < 0.001 (two-sided paired Student’s *t* test in C, E, and G). Data are pooled from two (*n* = 2 in J, L, and N) independent donors and are shown as means ± SD. See also Figures S8 and S9.

We next assessed whether we could use the same method to detect melanoma-specific CD8^+^ T cells in healthy donor blood without the use of MHC tetramers, i.e., T cells with undefined tumor specificity (Figure 7A). Based on CTV dilution, a much higher proportion of CD8^+^ T cells proliferated in response to Mel562 debris presented by helped as compared to non-helped DCs (Figures 7B and 7C; Figure S8A). The proliferating cells also displayed effector differentiation, defined by granzyme B and IFN-γ expression (Figures 7D–7G; Figure S8B). To assess killing capacity, DC-primed CD8^+^ cells were sorted and co-cultured with Mel526 cells for 24 h in the IncuCyte,^22^ monitoring live/dead cells in real time through captured images and measuring target cell killing based on caspase-3/7 activity (Figure 7H; Figure S8C). At the end of the assay, suspension cells were analyzed by flow cytometry, and adherent Mel526 cells were fixed and stained (Figure 7H). Proliferating CTV^(−)^CD8^+^ T cells were considered tumor reactive (Figure 7I) and showed higher granzyme B production and cell surface expression of the granule exocytosis marker CD107a after stimulation by helped versus non-helped DCs (Figure 7J). Accordingly, killing of Mel526 cells was shown by a loss of live tumor cell counts (Figures 7K and 7L), effector caspase-3/-7 activity (Figure 7M), and reduced Mel526 confluence (Figure 7N). In contrast, non-proliferating CD8^+^ T cells (CTV^+^) showed limited cytotoxicity regardless of help (Figure S9). Together, these results demonstrate that helped progenitor-derived DCs efficiently cross-prime tumor-specific CTLs from polyclonal CD8^+^ T cells in adult human blood, enabling effective tumor cell killing.

## Discussion

We have established a robust protocol to generate progeny that is enriched for cDC1-like cells from CD34^+^c-KIT^+^ progenitors, as present in non-mobilized peripheral blood of healthy adult donors. The progenitor-derived cDC1-like cell population generated consists of up to 60% of cDC1-like cells that resemble *ex vivo* cDC1s in their response to activated CD4^+^ T cells, in terms of upregulation of diverse molecules that are part of the cDC1 licensing program and involved in antigen (cross-)presentation and T cell priming. Importantly, when licensed by CD4^+^ T cells, progenitor-derived DCs potently cross-primed tumor-reactive CD8^+^ T cells from a polyclonal T cell repertoire as present in the blood of healthy donors and promote CTL effector differentiation. Our DC generation protocol and associated CTL-priming system therefore constitute a versatile platform to (1) enhance detection of tumor-specific CTLs in both circulation and tumor tissue, (2) qualitatively improve CD8^+^ T cell products for adoptive immunotherapy, and (3) broaden clinical access to cDC1-based cell vaccines.

cDC1s can home from peripheral tissues to LNs, cross-present particulate antigens to CD8^+^ T cells,^12^^,^^13^ and relay CD4^+^ T cell help for the CTL response.^19^ These combined properties make cDC1s the superior cell type to induce tumor-specific CTL response as compared to cDC2s, moDCs, and pDCs.^19^^,^^60^ While cDC1s are ideal candidates for cellular vaccines, their scarcity in peripheral blood severely limits their therapeutic use. In the field, efforts to generate human cDC1s from progenitors *in vitro* are ongoing and have already led to production of cells closely resembling *ex vivo* cDC1s, using mobilized adult PBMCs or cord blood as a progenitor cell source. We aimed to improve the protocol to enable use of non-mobilized adult blood, ideally not only from healthy donors but also from patients, to develop autologous systems. In a side-by-side comparison, our culture system, which adds MSCs to OP9-hDLL1 as feeder cells and uses an adapted cytokine cocktail, enabled the generation of cDC1-like cells from relatively low numbers of HPCs (∼1,000 cells) from non-mobilized adult blood, whereas a protocol described by Balan et al.^49^ did not. During finalization of our study, Balan et al. reported cDC1-like cell generation from non-mobilized blood using a serum-free, OP9 feeder cell-based culture system.^48^^,^^61^ Thus, we envision that an MSC-hDLL1 feeder system under serum-free conditions^61^ may provide a suitable platform for generating human cDC1s at sufficient yield in the future. Such integration could pave the way for defined large-scale production of cDC1s for clinical use. Future iterations may lead to replacement of feeder cells with defined cytokine cocktails. Developing feeder-free or fully defined culture conditions will be important to meet good manufacturing practice (GMP) standards and facilitate broader clinical applicability.

Our culture system also gives rise to SIRPα^+^ cDC2-like cells, while this study focused on bulk cDC1-like cells. As canonical markers for *ex vivo* DCs are less reliable for *in*-*vitro*-generated DCs, single-cell mRNA sequencing may help identify distinguishing new surface markers for *in vitro* cultures. Mouse models suggest that cDC2s are critical for priming CD4^+^ T cells,^21^ and DC subset crosstalk can enhance immune responses.^62^ Thus, incorporating both cDC1- and cDC2-like cells may offer synergistic benefits and better mimic physiological DC/T cell interactions in vaccine strategies.

Upon CD4^+^ T cell help, the progenitor-derived DC population enriched for cDC1-like cells expressed a transcriptional program that we previously defined in licensed/helped bona fide *ex vivo* human cDC1s.^19^ This program, as induced by CD4^+^ T cell help via CD40L and IFN-I, enhances cDC1 antigen cross-presentation, cross-priming, T cell recruitment, and costimulation.^21^^,^^22^^,^^63^ The cDC1 licensing program is conserved in mouse and humans.^19^^,^^20^^,^^21^^,^^22^^,^^24^^,^^25^ As a result, the cDC1 effectively induces CD8^+^ T cell expansion and CTL effector differentiation.^19^^,^^22^^,^^24^ We show here how progenitor-derived DCs enriched for cDC1-like cells can be used to identify and functionally program tumor antigen-specific CD8^+^ T cells. Using tumor debris as an antigen source and healthy donor blood as the T cell input, we detected rare tumor-specific CD8^+^ T cells, both by peptide/MHC tetramer staining and functional readouts (granzyme B and IFN-γ). Importantly, inclusion of CD4^+^ T cell help significantly increased detection rates, underscoring the impact of DC licensing on optimal CTL generation. Helped/licensed progenitor-derived DCs enriched for cDC1-like cells thus provide a sensitive tool for identifying tumor-specific CD8^+^ T cells in peripheral blood and tumor tissue, a long-standing challenge given their low precursor frequencies.^42^^,^^43^^,^^44^

Current strategies to detect and expand rare tumor-specific T cells predominantly rely on moDCs and B cells as APCs,^58^^,^^59^ largely due to their accessibility. However, as compared to cDC1s, moDCs^19^ and likely also B cells are less specialized in cross-presentation of cell-associated antigens and do not respond to CD4^+^ T cell help to upregulate cross-presentation and cross-priming abilities. Accordingly, in a side-by-side comparison with moDCs and B cells as APCs, our helped/licensed progenitor-derived DC platform showed higher sensitivity in detecting rare tumor antigen-specific CD8^+^ T cells, particularly in the presence of CD4^+^ T cell help.

Our methodology may benefit ACT by helping to identify circulating tumor-reactive CD8^+^ T cells that are increasingly leveraged for TCR-engineered therapies.^42^^,^^43^^,^^44^ Peripheral blood is currently being explored as an alternative to TILs.^64^^,^^65^ Notably, neoantigen-specific T cells from peripheral blood share phenotypic markers with TILs but are generally less terminally differentiated,^42^ potentially rendering them more expandable and therapeutically effective. Our platform could therefore facilitate the enrichment and isolation of such cells, or their TCRs, improving the quality of the input material for TCR- or TIL-based ACT. Moreover, CD8^+^ T cells primed by helped progenitor-derived DCs exhibited superior cytolytic activity. These functional gains are clinically meaningful, as ACT will benefit from the availability of optimally functional CTLs,^66^ and high-quality CTLs are more likely to persist and retain effector function within the TME, where chronic antigen exposure, cytokine-mediated suppression, and exhaustion pose significant barriers.^67^^,^^68^^,^^69^^,^^70^^,^^71^

In conclusion, generating human cDC1-like cells from progenitors and using them to deliver CD4^+^ T cell help enables sensitive detection of circulating tumor-reactive CD8^+^ T cells for TCR discovery or ACT and offers a strategy to enhance their functional quality. Future validation in cancer patients and incorporation of tumor-specific peptide/MHC multimers^72^ will further improve the application of this platform for personalized immunotherapy.

### Limitations of the study

The culture platform presented generates a DC population that is enriched for cDC1-like cells but is still heterogeneous and includes cDC2-like and monocytic cells. In addition, the cDC1-like cell culture relies on feeder cells, which may limit use of the method to generate GMP-compatible cell products. Furthermore, the broader clinical utility of this platform should be validated by the detection of diverse tumor (neo)antigen-specific CTL responses in blood and tumor material of cancer patients.

## Resource availability

### Lead contact

Requests for further information and resources should be directed to and will be fulfilled by the lead contact, Yanling Xiao (y.xiao@lumc.nl).

### Materials availability

This study did not generate new materials.

### Data and code availability

• All primary data are available from the lead contact upon request.
• This study does not report original code.
• Any additional information required to reanalyze the data reported in this paper is available from the lead contact upon request.

## Acknowledgments

This project was funded by an Antoni van Leeuwenhoek Foundation grant from the Bakker family to J.B. and Y.X. It was additionally funded by a grant from Oncode to J.B., 10.13039/501100004622Dutch Cancer Society (KWF) grant 11079 to J.B. and Y.X., the China Scholarship Consil (CSC)-Leiden University joint scholarship programme to Y.W.. We thank all personnel of the flow cytometry facilities at the Netherlands Cancer Institute and Leiden University Medical Center for their excellent technical support. We thank E. de Vries and L. Brocks for assistance. We thank S.J. van der Burg and J.W. Drijfhout for advice. Images and clipart used in the figures are derived from BioRender.com.

## Author contributions

Y.W. and X.L. designed and performed the experiments, analyzed the data, and wrote the manuscript. T.d.W., E.S., and K.v.L. assisted with cell sorting and co-culture experiments. J.v.d.B. and M.d.B. contributed to sample collection and performed the experiments. M.J.P.W., S.d.K., and N.F.C.C.d.M. provided scientific insight, with M.J.P.W. also assisting with Nanostring experiments. T.N.S. provided scientific insight and the MART-1-specific TCR construct. C.S. contributed to the experiments and provided scientific insight. J.B. interpreted the data and revised the manuscript. Y.X. designed and supervised the experiments, analyzed/interpreted the data, and wrote/revised the manuscript. Y.X. and J.B. designed the study and procured its funding. All authors reviewed the manuscript.

## Declaration of interests

The authors declare that the research was conducted in the absence of any commercial or financial relationships that could be construed as a potential conflict of interest.

## STAR★Methods

### Key resources table

**Table:** 

REAGENT or RESOURCE	SOURCE	IDENTIFIER
**Antibodies**		

Anti-Human CD1a Alexa Fluor 700	BioLegend	Cat#300120; clone HI149; RRID: AB_528764
Anti-Human CD1c BUV564	BD Biosciences	Cat# 750181; clone F10/21A3; RRID: AB_2874384
Anti-Human CD1c PE/Cy7	eBioscience	Cat#331515; Clone L161; RRID:AB_1953227
Anti-Human CD2 BV605	BioLegend	Cat# 300223; clone RPA-2.10; RRID:AB_2687242
Anti-Human CD3 BV650	BioLegend	Cat#317324; clone OKT3; RRID: AB_2563352
Anti-Human CD3 BV480	BD Biosciences	Cat#566166; clone UCHT1; RRID:AB_2739563
Anti-Human CD3 FITC	eBioscience	Cat# 509366; clone OKT3; RRID:AB_2016669
Anti-Human CD4 BV785	BioLegend	Cat#317408; clone OKT4; RRID: AB_571951
Anti-Human CD4 PE-Cy7	BioLegend	Cat#317442; clone OKT4; RRID:AB_2563242
Anti-Human CD5 BUV737	BD Biosciences	Cat# 748493; clone L17F12; RRID: AB_2872904
Anti-Human CD8 PerCP-Cy5.5	BioLegend	Cat#344710; clone SK1; RRID:AB_2044010
Anti-Human CD11c BUV737	BD Biosciences	Cat#568328; clone B-ly6; RRID: AB_3684184
Anti-Human CD11c Alexa Fluro 700	BioLegend	Cat#337220; clone Bu15; RRID:AB_2561503
Anti-Human CD11c APC-Fire750	BioLegend	Cat#301646; clone 3.9; RRID: AB_2814122
Anti-Human CD11c APC	BD Biosciences	Cat# 560895; clone B-LY6; RRID: AB_398680
Anti-Human CD14 FITC	BioLegend	Cat#367116; clone 63D3; RRID:AB_2571929
Anti-Human CD14 SparkUV 387	BioLegend	Cat#399216; clone S18004B; RRID:AB_2922626
Anti-Human CD19 BV650	BioLegend	Cat# 302238; clone HIB19; RRID: AB_2562097
Anti-Human CD19 FITC	eBioscience	Cat#: 11019182; clone MB19-1; RRID: AB_464965
Anti-Human CD25 PE	BioLegend	Cat# 302606; clone BC96; RRID:AB_314276
Anti-Human CD34 Pacific Blue	BioLegend	Cat#343512; clone 581; RRID: AB_1877197
Anti-Human CD40 BV711	BioLegend	Cat#334334; clone 5C3; RRID: AB_2564212
Anti-Human CD45 PE-CF594	BD Biosciences	Cat#562279; clone HI30; RRID: AB_11154577
Anti-Human CD45RA BV510	BioLegend	Cat# 304142; clone HI100; RRID:AB_2561947
Anti-Human CD45RA BV650	BioLegend	Cat# 304136; clone HI100; RRID: AB_2563653
Anti-Human CD45RO PE-CF 594	BD Biosciences	Cat#562299; clone UCHL1; RRID:AB_11154398
Anti-Human CD70 PerCP-Cy5.5	BioLegend	Cat#355108; clone 113-16; RRID:AB_2562479
Anti-Human CD80 BV785	BioLegend	Cat#305238; clone 2D10; RRID:AB_2734272
Anti-Human CD83 BV421	BioLegend	Cat#305324; clone HB15e; RRID:AB_2561829
Anti-Human CD86 BV510	BioLegend	Cat#305432; clone IT2.2; RRID:AB_2562064
Anti-Human CD88 BV711	BD Biosciences	Cat# 742319; clone D53-1473; RRID: AB_2874389
Anti-Human CD107α PerCP-Cy5.5	BioLegend	Cat#328638; clone H4A3; RRID:AB_2565838
Anti-Human CD117 (C-Kit) PE-Cy7	eBioscience	Cat# 25-1178-42; clone 104D2; RRID: AB_10718535
Anti-Human CD123 PerCP	BioLegend	Cat# 306050; clone 6H6; RRID:AB_2894455
Anti-Human CD141 RB613	BD Biosciences	Cat# 759328; clone 1A4; RRID: AB_3691416
Anti-Human CD141 PE-Fire810	BioLegend	Cat# 344133; clone M80; RRID:AB_2927907
Anti-Human CD163 PE-Dazzle594	BioLegend	Cat# 333623; clone GHI/61; RRID: AB_2564200
Anti-Human CD172α (SIRPα) APC	BioLegend	Cat# 372105; clone 15-414; RRID: AB_2650862
Anti-Human CD197(CCR7) PerCP-Cy5.5	BioLegend	Cat#353220; clone G043H7; RRID:AB_10916121
Anti-Human CD206 PE-Fire700	BioLegend	Cat# 21770; clone 15-2; RRID:AB_2910389
Anti-Human CD274 (PD-L1) BV711	BioLegend	Cat# 329722; clone 29E.2A3; RRID: AB_2565764
Anti-Human CD303 BV786	BD Biosciences	Cat# 748000; clone V24-785; RRID: AB_2872461
Anti-Human CD370 (CLEC9A) BV421	BD Bioscience	Cat# 564266; clone 3A4; RRID: AB_2738716
Anti-Human CD370 (CLEC9A) PE	BioLegend	Cat# 353803; clone 8F9; RRID: AB_10959651
Anti-Human AXL BV421	BD Biosciences	Cat# 747864; clone 108724; RRID: AB_2872326
Anti-Human β2m PE	BD Biosciences	Cat# 551337; clone TÜ99; RRID: AB_394152
Anti-Human CXCL9 PE	BioLegend	Cat#357904; clone J1015E10; RRID:AB_2562009
Anti-Human CXCL10 PE	BioLegend	Cat#519503; clone J034D6; RRID: AB_2561408
Anti-Human DLL1 PE	BioLegend	Cat# 346403; clone MHD1-314; RRID: AB_2092809
Anti-Human Granzyme B PE-Cy7	BioLegend	Cat#372208; clone QA16A02; RRID:AB_2687032
Anti-Human HLA-A2 FITC	BD Biosciences	Cat# 551285; clone BB7.2; RRID: AB_394130
Anti-Human HLA-ABC FITC	BioLegend	Cat#311404; clone W6/32; RRID:AB_314873
Anti-Human HLA-DR Spark Violet 538	BioLegend	Cat# 20388; clone L243; RRID: AB_2561913
Anti-Human HLA-DR BV605	BD Biosciences	Cat#562845; clone G46-6; RRID: AB_2744478
Anti-Human HLA-DR RB744	BD Biosciences	Cat#758140; clone L243; RRID: AB_3690294
Anti-Human HLA-DR APC-Cy7	BioLegend	Cat#307618; clone L243; RRID:AB_493586
Anti-Human IFNγ FITC	BioLegend	Cat# 506504; clone B27; RRID: AB_315437
Anti-Human IRF8 PE-Cy7	eBioscience	Cat# 25-9852-82; clone V3GYWCH; RRID: AB_2784675
Anti-Human FLT3 RY703	BD Biosciences	Cat# 772019; clone 4G8; RRID: AB_3693838
Anti-Human Siglec-6 BV786	BD Biosciences	Cat# 747909; clone 767329; RRID: AB_2872371
Anti-Human XCR1 PE	BioLegend	Cat# 372603; clone S15046E; RRID: AB_2632710
Anti-Human XCR1 BV421	BioLegend	Cat#372610; clone S15046E; RRID:AB_2687373
goat anti-mouse IgG(H+L) Alexa Fluor 647	Invitrogen	Cat#A-21235; RRID: AB_2535804
goat anti-rabbit IgG(H+L) Alexa Fluor 488	Invitrogen	Cat#A-11008; RRID: AB_143165
TAP1 polyclonal antibody	Bioss Antibodies	Cat#bs-2789R; RRID: AB_10857457
TAP2 polyclonal antibody	Bioss Antibodies	Cat#bs-2374R; RRID: AB_10857127
Anti-Human CD3	Sanquin	Cat#M1654 clone CLB-T3/4.E
Anti-Human CD28	Sanquin	Cat#M1650 clone CLB-CD28/1

**Biological samples**		

Human peripheral blood samples	Sanquin	N/A

**Chemicals, peptides, and recombinant proteins**		

Recombinant human IL-2	Miltenyi Biotec	Cat#130-097-743
Recombinant human IL-4	ImmunoTools	Cat# 11340043
Recombinant human IL-7	Miltenyi Biotec	Cat#130-093-937
Recombinant human IL-15	Miltenyi Biotec	Cat#130-095-760
Recombinant human IL-21	Miltenyi Biotec	Cat# 130-095-767
Recombinant human Flt3-Ligand	Miltenyi Biotec	Cat#130-093-854
Recombinant human M-CSF	Miltenyi Biotec	Cat#130-093-963
Recombinant human TPO	Miltenyi Biotec	Cat#130-094-013
Recombinant human SCF	Miltenyi Biotec	Cat#130-096-692
Recombinant human GM-CSF	Miltenyi Biotec	Cat#130-095-372
AF488 or APC-conjugated HLA-A2/MART-1_26-35_ tetramers	Peptide facility in LUMC	N/A
APC or PE-conjugated HLA-A2/CDK4_23-32_ tetramers	Peptide facility in LUMC	N/A
PE-conjugated HLA-A2/MAGE3 tetramers	Peptide facility in LUMC	N/A
MART-1_26-35_ short peptide	Peptide facility in LUMC	N/A
MART-1_15-40_ long peptide	Peptide facility in LUMC	N/A
IMDM	Gibco	Cat#12440053
RPMI-1640	Gibco	Cat#11875093
Fetal calf serum (FCS)	Serana	Cat#S-FBS-SA-010
Trypsin	Gibco	Cat#11560626
β-mercaptoethanol	Sigma-Aldrich	Cat#M6250
Penicillin/streptomycin	Gibco	Cat#15140122
Dimethyl sulfoxide (DMSO)	Dimethyl sulfoxide	Cat#472301
CellTrance Violet(CTV)	Invitrogen	Cat#C34557
7-amino-actinomycin D (7AAD)	eBioscience	Cat#00-6993-50
Zombie Red Fixable Viability Kit	BioLegend	Cat#423109
Zombie UV Fixable Viability Kit	BioLegend	Cat#423107
Near-IR Dead Cell Stain Kit	Invitrogen	Cat#L10119
UltraComp eBeads™ Compensation Beads	Invitrogen	Cat#01-2222-42
CD14 magnetic MicroBeads	Miltenyi Biotec	Cat#130-097-052
CD19 magnetic MicroBeads	Miltenyi Biotec	Cat#130-050-301
eBioscience™ Foxp3 / Transcription Factor Staining Buffer Set	Invitrogen	Cat#00-5523-00
Protein Transport Inhibitor (BD GolgiPlug)	BD Biosciences	Cat#555029
BD Cytofix/Cytoperm kit	BD Biosciences	Cat#554714
Buffer RLT	QIAGEN	Cat#79216
TRAIL	Sigma	Cat#T9701
FASL	AdipoGen	Cat#AG-40B-0001-C010
Phorbol 12-myristate 13-acetate (PMA)	Sigma	Cat#79346
Ionomycin	InvivoGen	Cat#inh-ion
LPS	InvivoGen	Cat#tlrl-peklps
poly(I:C) HMW	InvivoGen	Cat#tlrl-pic
R848	InvivoGen	Cat#tlrl-r848
Caspase-3/7 Green Apoptosis Reagent	Satorius	Cat#4440
4% PFA	Sigma	Cat#1.00496
Red blood cell lysis buffer	Santa Cruz Biotechnology	Cat#sc-296258
Ficoll-paque™ Plus	Cytiva	Cat#17-1440-02
nCounter host response panel	Nanostring	N/A

**Experimental models: Cell lines**		

Melanoma526	ATCC	RRID:CVCL_8051

**Software and algorithms**		

GraphPad Prism (v 10.2.3)	GraphPad software	https://www.graphpad.com
FlowJo™ software (v 10.10.0)	BD Biosciences	https://www.flowjo.com/solutions/flowjo/downloads
OMIQ flow cytometry software	Dotmatics	https://www.omiq.ai/
nSolver advanced analysis module (v 1.1.4)	Nanostring	https://nanostring.com/products/analysis-solutions/ncounter-analysis-solutions/nsolver-data-analysis-support/
Incucyte S3 software (v2018B)	Sartorius	https://www.sartorius.com/
limma R packages (v 3.58.1)	Ritchie ME et al. Nucleic Acids Res. 2015	https://bioconductor.org/packages/limma
fgsea R packages (v 1.28.0)	Korotkevich et al., bioRxiv, 2019	https://bioconductor.org/packages/fgsea
ggplot2 R packages (v 3.5.1)	ggplot2: Elegant Graphics for Data Analysis, 2016	https://ggplot2.tidyverse.org
pheatmap T package (v1.0.12)	CRAN	https://cran.r-project.org/package=pheatmap
R (v 4.1.2)	CRAN	https://www.r-project.org/

### Experimental model and study details

#### Human peripheral blood samples

Buffy coats were obtained from adult healthy donors after written informed consent at Sanquin, in accordance with the Declaration of Helsinki and Dutch regulations for the use of human materials, and were anonymized before release to the researchers. PBMC were isolated from buffy coats using Ficoll-Paque Plus (GE Healthcare). HPC, monocytes and B cells were isolated from HLA-A2^+^ donors, while CD4^+^ and CD8^+^ T cells were used regardless of HLA type and donor origin. Donor matching varied by assay and is specified in the relevant method sections.

#### Feeder cells

OP9-hDLL1 cells were generated by retroviral transduction of OP9 cells with hDLL1 construct. MSC were isolated from healthy donors’ bone marrow.^52^ CD40L-L cells were generated by transduction of mouse L cell fibroblasts with CD40L construct.

### Method details

#### *In vitro* generation of blood progenitor-derived DC

Human MSC, OP9, and OP9-hDLL1 were harvested using 0.05% trypsin. MSC were irradiated at 16 Gy, and OP9/OP9-hDLL1 at 30 Gy, prior to plating as feeder cells. OP9 and OP9-hDLL1 cells were mixed at a 3:1 ratio for culture method 1 and 2 (Figure S1A**)**. MSC and OP9-hDLL1 were mixed at a 3:1 ratio for culture method 3. Feeder cells were then seeded at 3000 cells/well in round-bottom 96-well plates in Iscove’s Modified Dulbecco’s Medium (IMDM) supplemented with 10% fetal calf serum (FCS), 100 U/mL penicillin, 100 μg/mL streptomycin, and 50 μM β-mercaptoethanol. After 24 h, the feeder cell layer was gently washed with the same medium to remove non-adherent cells, and CD3^-^CD19^-^CD34^+^c-KIT^+^ HPC (progenitors, 1000 cells/well), sorted from PBMC, were seeded on top of the feeder cells in the presence of the indicated cytokine cocktails. For culture method 1, freshly sorted progenitors were initially seeded at 1000 cells/well without feeders, with cytokines, and whole well transferred to the OP9/OP9-hDLL1 feeder layer after 7 days. DC differentiation cytokines were used at final concentrations of 100 ng/mL FLT3L, 5 ng/mL each of TPO, SCF and GM-CSF, 1 ng/mL of IL-7 and 0.5 ng/mL M-CSF. For experiments comparing all different culture methods, cytokines were present throughout the culture period as indicated in Figure S1A. Half of the medium was refreshed every 6-7 days with fresh cytokine-containing medium. Cultures were maintained at 37°C with 5% CO_2_ for 14–21 days before analysis. Generated progenitor-derived DCs were used for phenotypic, functional, and co-culture assays as indicated.

#### Flow cytometry staining and cell sorting

For sorting HPC, CD4^+^ T cells and CD8^+^ T cells, CD19^+^ cells were depleted from PBMC using CD19 MicroBeads (Miltenyi Biotec). Cells were then incubated in BD staining buffer with fluorochrome-conjugated monoclonal antibodies (mAb) at 4°C for 45 min and sorted on a BD FACS Aria III. HPC were defined as CD3^-^CD19^-^CD34^+^c-KIT^+^ cells. Naïve CD4^+^ T cells as CD3^+^HLA-DR^-^CD4^+^CD25^-/low^ CD45RA^+^. Naïve CD8^+^ T cells as CD3^+^HLA-DR^-^CD8^+^CD25^-/low^ CD45RA^+^ and total CD8^+^ T cells as CD3^+^HLA-DR^-^CD8^+^ cells.

For flow cytometry analysis, cells were stained with mAbs against surface and intracellular markers, or tetramers. Distinct antibody panels (Table S5) and related reagents (key resources table) were used to characterize DC phenotype, DC activation, and T-cell priming. For intracellular staining, DC were incubated with GolgiPlug (1:1000, BD Biosciences) ), whereas CD8^+^ T cells were stimulated for 3 h with 50 ng/ml PMA, 1 μg/ml Ionomycin, and GolgiPlug (1:1000, BD Biosciences). Cells were subsequently fixed and permeabilized using the Cytofix/Cytoperm kit (BD Biosciences). TAP1/2 detection required secondary antibody staining, validated by fluorescence minus one (FMO) controls. Dead cells were excluded using viability dye. Compensation beads, unstained cells, single stained cells and FMO were included for compensation and gating. Data acquisition was performed on BD LSR Fortessa™, Cytek Aurora spectral flow cytometers or SONY ID7000™ Spectral Cell Analyzer and analyzed using FlowJo™ or OMIQ software.

#### NanoString nCounter gene expression analysis

Progenitor-derived DC were cocultured with CD4^+^ T cells for 12 h, then sorted using HLA-DR, CD3, and 7-AAD markers**.** Cells were lysed in 5-6 μl of 1/3 diluted RLT buffer (QIAGEN) at 2000 cells/μl and then hybridized with probes according to NanoString nCounter’s user manual for whole cell lysates. Samples were analyzed with the NanoString nCounter MAX platform using the Human Host Response Panel (773 genes, Table S1). RNA counts were normalized with nSolver (v1.1.4) and log2-transformed data were analyzed in R (v4.1.2). DEGs (Table S2**)** were identified using ‘limma’ (p < 0.05, log2FC > 1 or < –1). Volcano plots and heatmaps were generated in R (v4.1.2) using ‘ggplot2’ and ‘pheatmap’. The cDC1 “help” signature from a prior study^19^ was used (Tables S3 and S4). Reactome pathway enrichment was performed with ‘fgsea’ (v1.28.0).

#### Tumor antigen-specific CTL priming platform

This method was adapted from our previous publication^19^, CD8^+^ T cells were either retrovirally transduced to express the MART-1_26-35_/HLA-A2-specific TCR, or used as polyclonal population. CD8^+^ T cells were cultured in RPMI-1640 with 10% FBS and human IL-2/IL-7/IL-15 (each at 10 ng/ml) before use. Naïve CD4^+^ T cells were cultured in the presence of agonistic mAbs against CD3 (0.1 μg/ml) and CD28 (0.2 μg/ml) for 24-36 h to induce CD4^+^ T-cell activation. MART-1_26-35_ short peptide (SP, ELAGIGILTV, 500 ng/ml), MART-1_15-40_ long peptide (LP, KGHGHSYTTAEELAGIGILTV, 20 μg/ml) or debris of MART-1 expressing Mel526 cells was used as antigen source. Mel526 cell debris was prepared as previously described.^19^

HLA-A2^+^ progenitor-derived DC were co-cultured with either activated or naïve CD4^+^ T cells at a 1:1 ratio for 2 h IMDM supplemented in 1% FBS, followed by adding tumor antigen along with PRR stimuli consisting of 50 ng/ml LPS (InvivoGen), 20 μg/ml poly I:C (InvivoGen) and 3 μg/ml R848 (InvivoGen). To trace proliferation, CD8^+^ T cells were labeled with CTV before being addition to the co-culture platform. After 12-16 h, culture supernatants were removed and CTV-labeled CD8^+^ T cells were added at a 1:10 DC: T-cell ratio. Cultures were maintained for 6-7 days in RPMI-1640 supplemented with 10% FCS, 2 mM L-glutamine and IL-7/IL-15 (each 0.2 ng/ml). Cells were then harvested, stained, and analyzed by flow cytometry. All reagents are listed in key resources table.

#### Antigen presenting cell comparison for tumor specific CTL detection and expansion

B cells were isolated from HLA-A2^+^ PBMC by positive selection using CD19 MicroBeads (Miltenyi Biotec), followed by isolation of monocytes from the remaining fraction using CD14 MicroBeads (Miltenyi Biotec), according to the manufacturer’s instructions. Purified CD19^+^ B cells (2x10^6^ per well) were cultured for 7 days on irradiated (55 Gy) CD40L-expressing fibroblasts^60^ (5x10^5^ per well) in 6-well plates in the presence of IL-21 (25 ng/mL). Purified CD14^+^ monocytes (5x10^6^ per well) were cultured for 7 days in 6-well plates in the presence of GM-CSF (100 ng/mL) and IL-4 (50 ng/mL) to generate moDC.^19^ Cells were cultured in complete IMDM supplemented with 10% FCS, 100 U/mL penicillin, 100 μg/mL streptomycin and 50 μM β-mercaptoethanol at 37°C and 5% CO_2_, and cytokine-containing medium was refreshed on day 4. Presence of activated B cells and moDC in the cultures was assessed by flow cytometry on day 7.

To compare APC function, progenitor-derived DC, activated B cells and moDC obtained from the same donors (n=3) were plated at 100,000 cells per well in 48-well plates and co-cultured with either activated or naïve CD4^+^ T cells at a 1:1 ratio for 2 h in IMDM supplemented with 1% FBS. Subsequently, Mel526 cell debris and PRR stimuli were added as described in the previous section. After 12-16 h, culture supernatants were removed, and CTV-labeled polyclonal CD8^+^ T cells were added at an APC:T cell ratio of 1:10. Cultures were maintained for 6 days in RPMI-1640 supplemented with 10% FCS, 2 mM L-glutamine, and IL-7 and IL-15 (0.2 ng/mL each). MART1-specific CD8^+^ T-cell responses were assessed by flow cytometry using MART-1/HLA-A2 tetramer staining, in combination with Granzyme B staining and CTV dilution.

#### Allogeneic mixed lymphocyte reaction

Progenitor-derived DC from donors X1-X3 were co-cultured with allogeneic activated or naïve CD4^+^ T cells from donors Y1-Y3 at a 1:1 ratio for 2 h in complete IMDM containing 1% FBS. Subsequently, CTV-labeled allogeneic naïve CD8^+^ T cells from the same donors (Y1–Y3) were added at a DC:T cell ratio of 1:10. Cultures were maintained for 6 days in RPMI-1640 supplemented with 10% FCS, 2 mM L-glutamine, and low-dose IL-7 and IL-15 (0.2 ng/mL each) to support T cell survival. Naïve CD8^+^ T-cell priming was assessed by flow cytometry based on CTV dilution.

#### IncuCyte T-cell killing assay

Mel526 cells were seeded at 5000 single cells/well in 96-well plates. The next day, medium was removed and sorted CD8^+^ T cells were added at a 4:1 effector: target ratio and IncuCyte Caspase-3/7 Green Apoptosis Reagent (20 μM, Sartorius) in culture medium. Plates were incubated for 30 min at room temperature and next imaged in the IncuCyte ZOOM® (Sartorius) every 2 h for 24 h. Afterwards, suspend cells were collected for flow cytometry; remaining Mel526 cells were fixed with 4% PFA (Sigma), stained with crystal violet, and imaged by CCD microscopy (Zeiss Axio Imager Z1). Data analysis was done using IncuCyte S3 Software (v2018B).

### Quantification and statistical analysis

Data were analyzed with GraphPad Prism 9.0 using Student’s t-test (paired or unpaired), one-way ANOVA or two-way ANOVA, followed by Tukey’s multiple comparisons test for group comparisons. All data are presented as means ± SEM or SD; ∗*p* < 0.05; ∗∗*p* < 0.01; ∗∗∗*p* < 0.001; ∗∗∗∗*p* < 0.0001, *p* > 0.05 not shown.

## Supplemental information

Document S1. Figures S1–S9

Table S1. NanoString nCounter Host Response Panel, related to Figure 3

Table S2. Differentially expressed genes in progenitor-derived DCs co-cultured with activated versus naive CD4^+^ T cells, related to Figure 3

Table S3. Gene list of the *ex vivo* cDC1 “help” signature represented in the NanoString nCounter panel, related to Figure 3

Table S4. Full gene list of the *ex vivo* cDC1 “help” signature, related to Figure 3

Table S5. Antibody panels used to characterize DC phenotype, DC activation, and T cell priming, related to STAR Methods

Document S2. Article plus supplemental information
