# Supplementary material for: Efficient generation of human cDC1-like cells to detect and enhance tumor-reactive T cells via CD4+ T cell help
Source: Cell Rep Methods. 2026 Jul 22;6(9):101533. doi: 10.1016/j.crmeth.2026.101533 (PMC13615486; doi:10.1016/j.crmeth.2026.101533)
Supplement: Supplementary file 1 [file mmc1.pdf]

Cell Reports Methods, Volume *6*

Supplemental information

Efficient generation of human cDC1-like cells to detect and enhance tumor-reactive T cells via $CD4^+$ T cell help

Yizhi Wang (汪逸之), Xin Lei (雷馨), Tom de Wit, Ellen Schrama, Marij J.P. Welters, Karel van Leeuwen, Sander de Kivit, Miriam de Boer, Jitske van den Bulk, Noel F.C. C. de Miranda, Ton N. Schumacher, Chong Sun (孙崇), Jannie Borst, and Yanling Xiao (肖艳灵)

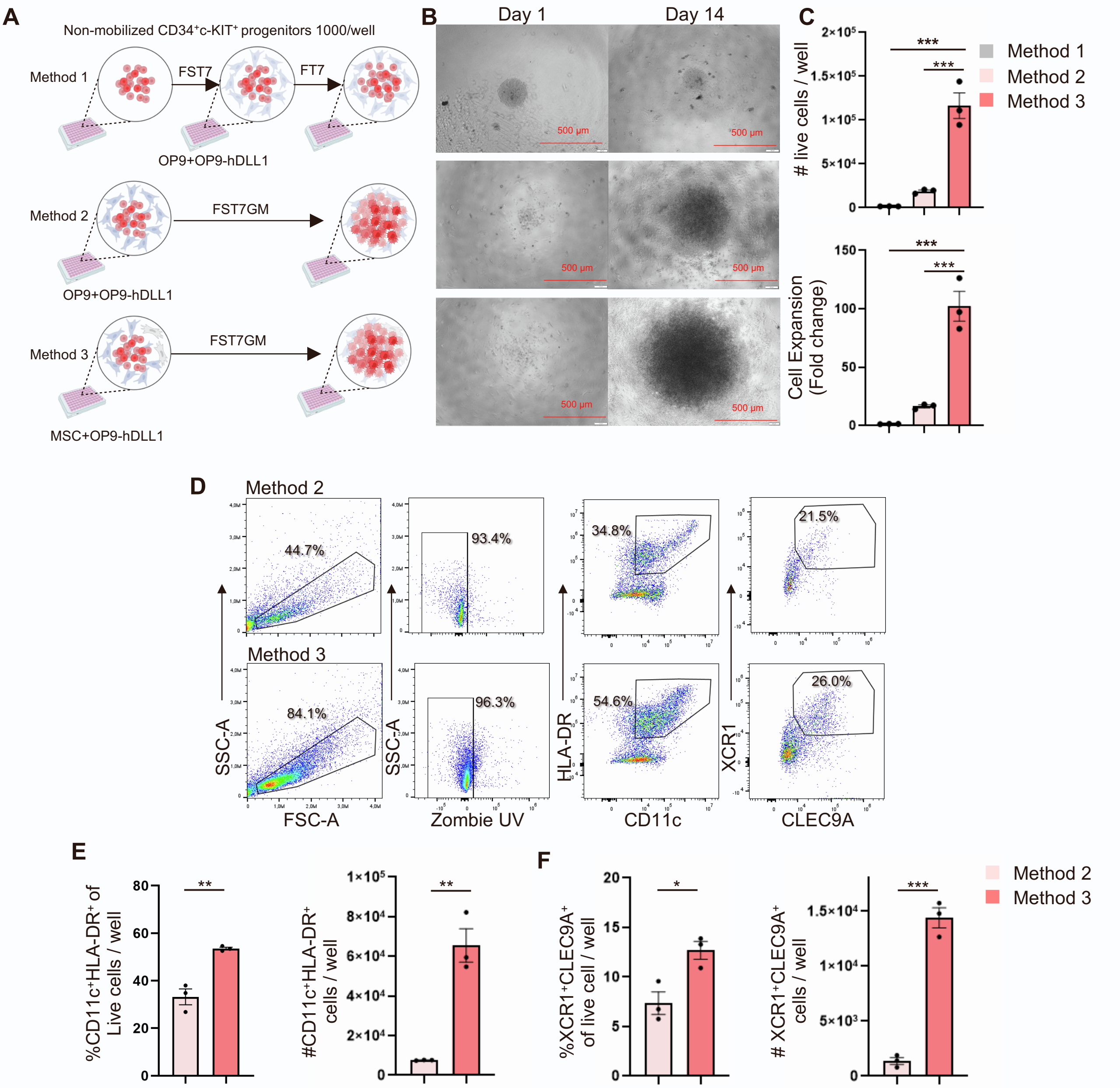

Figure S1. Optimizing DC generation from non-mobilized PBMC progenitors, related to Figure 1.
DC generation from non-mobilized PBMC-derived CD34$^+$c-KIT$^+$ progenitors were compared under various conditions.
(**A**) Schematic representation of three co-culture strategies for *in vitro* DC differentiation. Method 1 follows protocol established by Balan *et al.*[1], using OP9+OP9-hDLL1 feeder layers and sequential cytokine cocktails. Method 2 uses the same feeder composition but uses a single cytokine mix throughout the procedure. Method 3 incorporates a modified feeder composition (human MSC+OP9-hDLL1) along with a single cytokine mix. For clarity, the cytokines are as follows: F (Flt3L), S (SCF), T (TPO), 7 (IL-7), G (GM-CSF), M (M-CSF).
(**B**) Microscopic pictures depicting the growth of progenitor-derived DC formed on Day 1 and Day 14 in each culture condition (scale bar: 500 μm).
(**C**) Quantification by counting of total live cells per well (up) and fold expansion of total cells (below) after 14 days of culture.
(**D**) Representative flow cytometry plots showing gating strategy for method 2 and method 3. Note: method-1 did not generate sufficient cells for flow cytometry analysis.
(**E**) % Frequency among live cells (left) and # absolute number (right) of CD11c$^+$HLA-DR$^+$ cells per well.
(**F**) % Frequency among live cells (left) and # absolute number (right) of XCR1$^+$CLEC9A$^+$ cells per well.
Data are shown in C-F (n=3) as mean ± SEM, with each dot representing one donor. *$p<0.05$, **$p<0.01$, ***$p<0.001$ by one-way ANOVA with Tukey's multiple comparisons in C, two-sided unpaired Student's t-test in E,F.

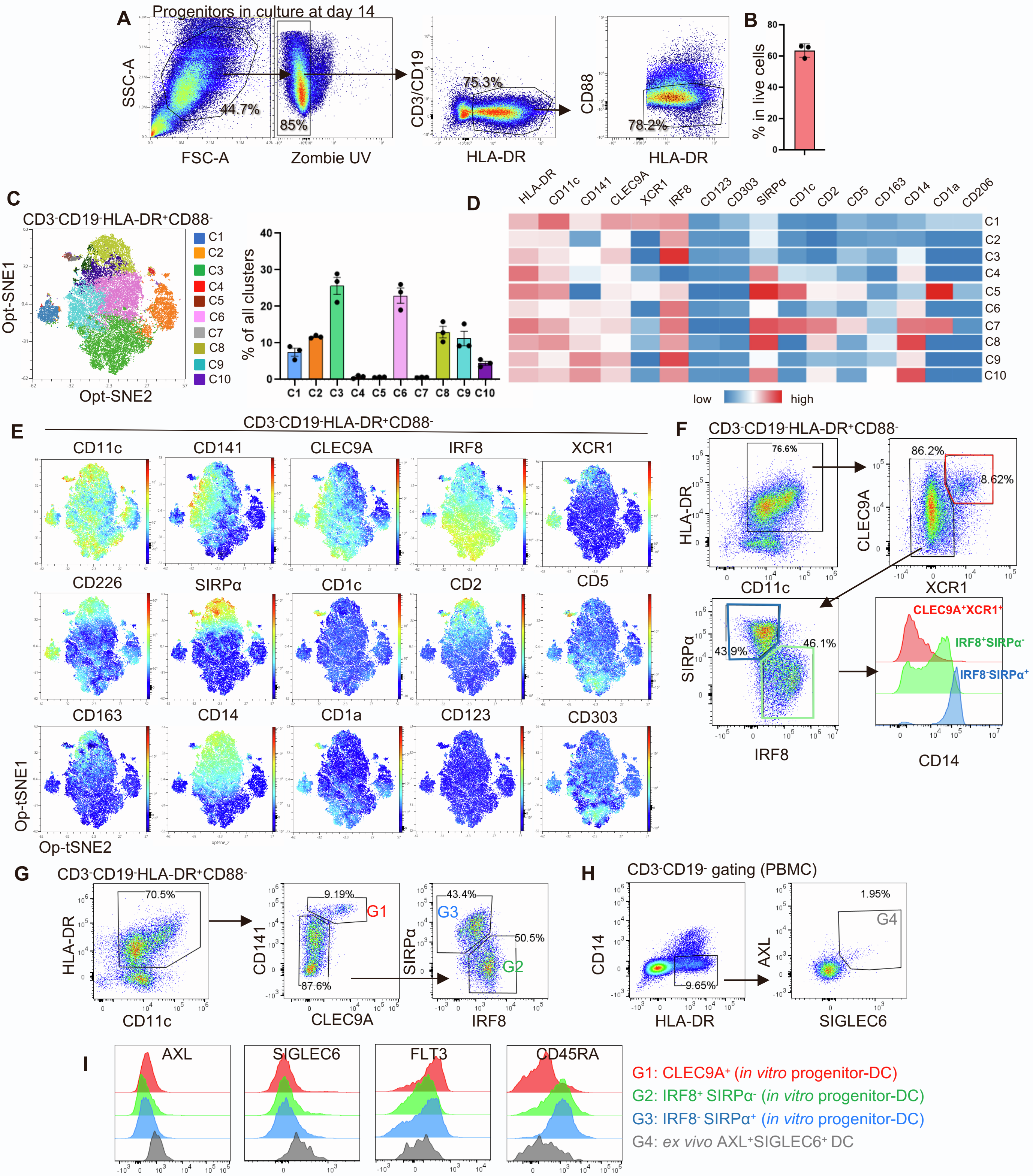

Figure S2. Generating human cDC1-like cells from non-mobilized blood-derived progenitors *in vitro,* related to Figure 1.

Phenotypic analysis was performed to assess heterogeneity within the CD3$^-$CD19$^-$HLA-DR$^+$CD88$^-$ DC compartment.

(**A**) Gating strategy for progenitor-derived DC phenotype analysis.

(**B**) Quantification of frequencies (%) of CD3$^-$CD19$^-$HLA-DR$^+$CD88$^-$ DC progeny in live cells.

(**C**) Opt-SNE plot (left) of 10 clusters identified within CD3$^-$CD19$^-$HLA-DR$^+$CD88$^-$ progeny cells and associated frequencies (%, right) of 10 clusters in HLA-DR$^+$CD88$^-$ DC progeny.

(**D**) Heatmap of median expression values of key phenotypic markers identifying cDC1-like and other DC types.

(**E**) Opt-SNE plots depicting expression of indicated markers on CD3$^-$CD19$^-$HLA-DR$^+$CD88$^-$ population.

(**F**) Gating strategy used to define populations corresponding to clusters C1, C3, C6 and C9 identified by Opt-SNE plots, and representative histogram showing CD14 expression for CLEC9A$^+$XCR1$^+$, SIRPα$^-$IRF8$^+$ and SIRPα$^-$IRF8$^+$ subpopulations.

(**G**) Subpopulation gating strategy of progenitor-derived DC to assess the expression of pre-DC associated markers.

(**H**) Gating strategy for identifying AXL$^+$SIGLEC6$^+$ pre-DC populations in *ex vivo* blood samples.

(**I**) Representative histogram showing expression of AXL, SIGLEC6, FLT3 and CD45RA in progenitor-derived DC.

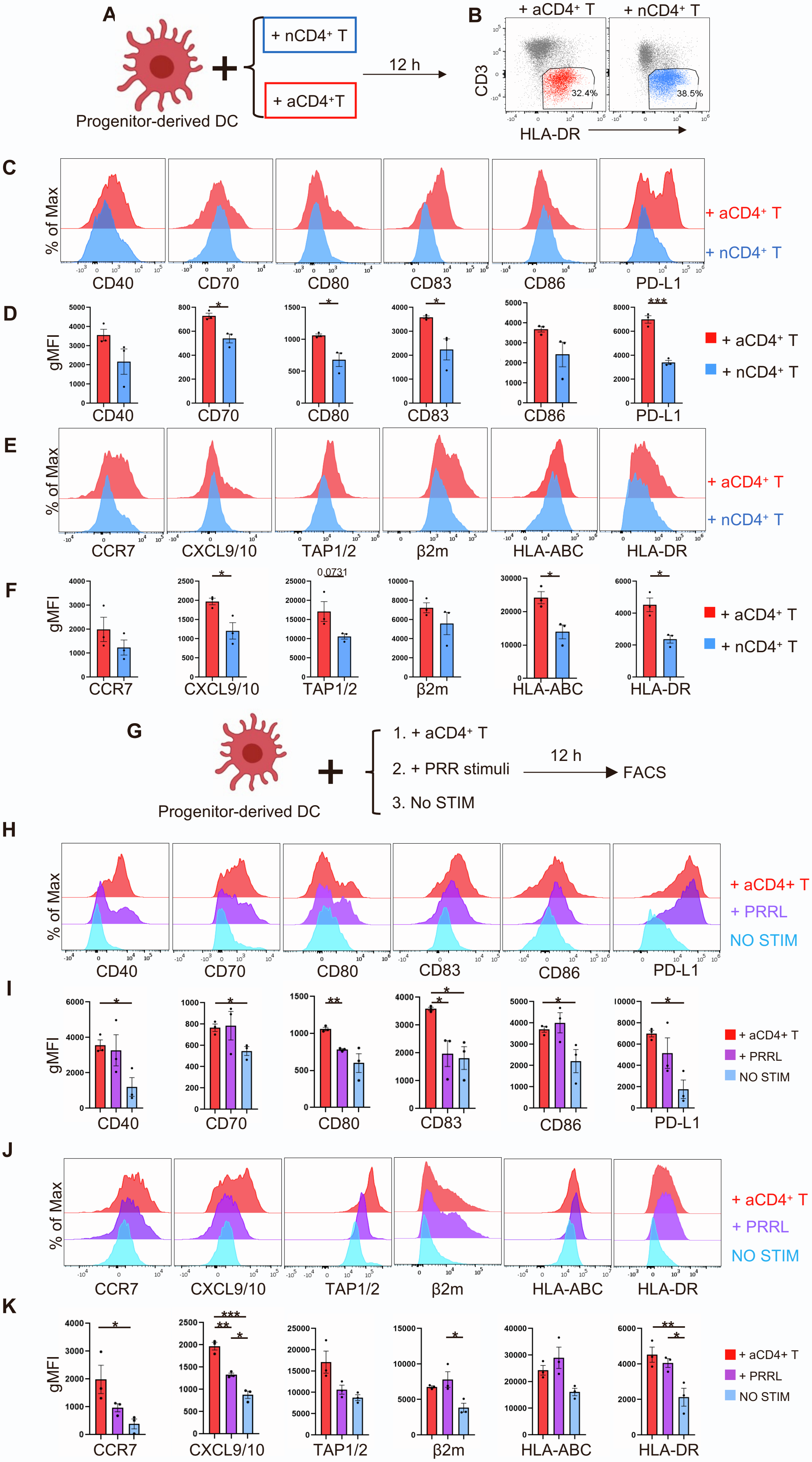

A
Progenitor-derived DC
+ nCD4⁺ T
+ aCD4⁺T
12 h
B
+ aCD4⁺ T
+ nCD4⁺ T
CD3
HLA-DR
32.4%
38.5%
C
% of Max
CD40
CD70
CD80
CD83
CD86
PD-L1
+ aCD4⁺ T
+ nCD4⁺ T
D
gMFI
E
CCR7
CXCL9/10
TAP1/2
β2m
HLA-ABC
HLA-DR
F
0.0731
G
Progenitor-derived DC
1. + aCD4⁺ T
2. + PRR stimuli
3. No STIM
12 h
FACS
H
+ aCD4+ T
+ PRRL
NO STIM
I
+ PRRL
NO STIM
J
K

Figure S3. Response of progenitor-derived cDC1-like cells to activated CD4⁺ T cells, related to Figure 2.
Progenitor-derived DCs were co-cultured overnight either with activated (a) or naïve (n) $CD4^+$ T cells, or under the indicated stimulation conditions including activated $CD4^+$ T cells or PRR stimuli. Next, key molecules of the cDC1 "help" signature[2] were analyzed by flow cytometry.
(**A**) Experimental design for **C**-**F**.
(**B**) Gating strategy for flow cytometric analysis of the $CD3^-HLA\text{-}DR^+$ progenitor-derived DC population after co-culture.
(**C-F**) Histograms (**C**, **E**) and geometric mean fluorescence intensity (gMFI) quantifications (**D**, **F**) for indicated markers expressed by progenitor-derived DC under indicated conditions.
(**G**) Experimental design for **H**-**K**.
(**H**-**K**) Histograms (**H** and **J**) and gMFI quantifications (**I** and **K**) for indicated markers expressed by progenitor-derived DC under indicated conditions.
Data are pooled from three (n=3) independent donors (D, F, I, K). Data are shown as means ± standard error of mean (SEM). *p<0.05, **p<0.01, ***p<0.001. Statistics analyses were performed using a two-sided unpaired Student's t-test for panels (D) and (F), one-way ANOVA with Tukey's multiple comparisons for panels (I) and (K).

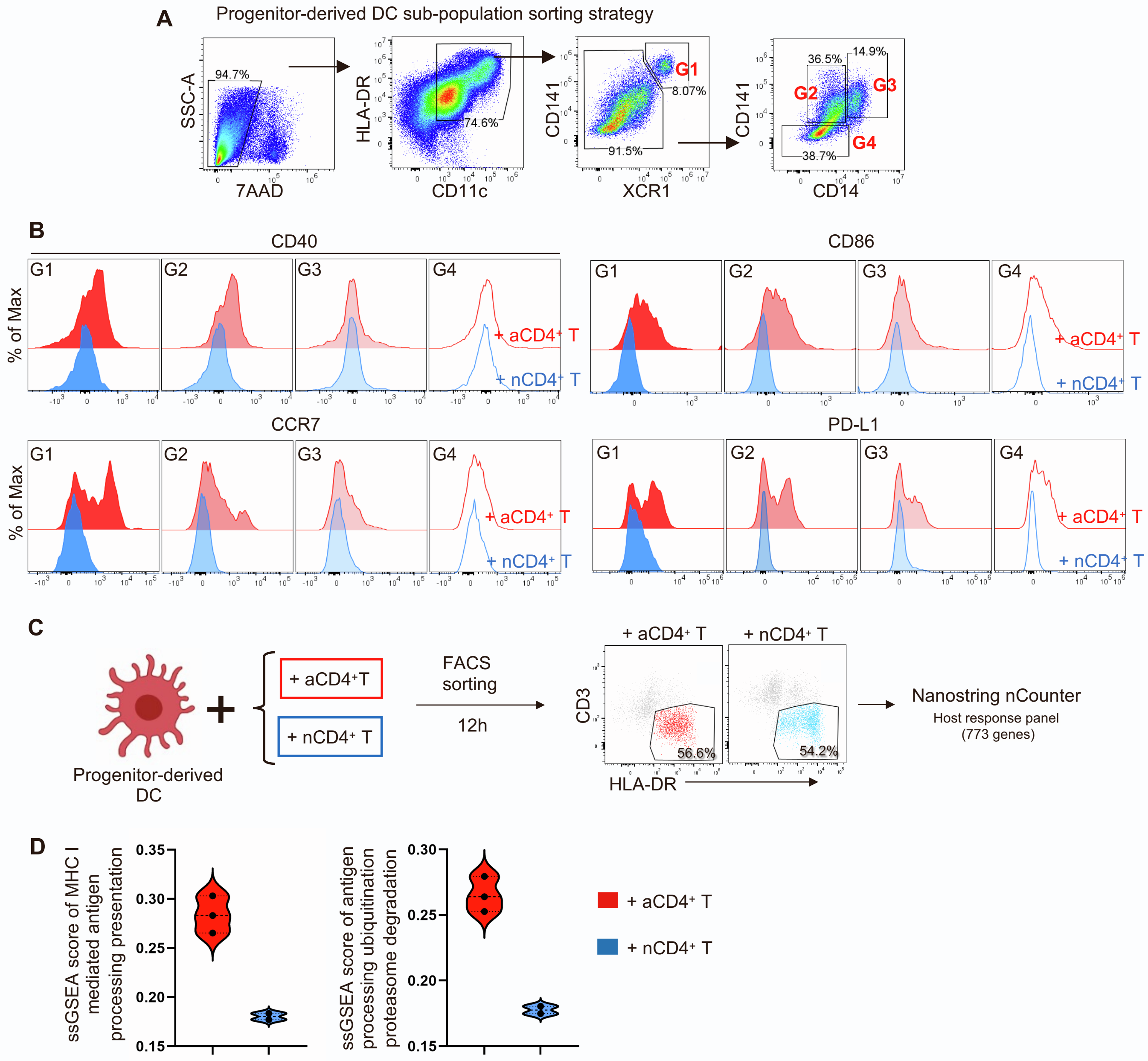

Figure S4. Responses of sorted progenitor-derived DC to activated CD4⁺ T cells, related to Figure 2 and 3. Sorted progenitor-derived DC populations were examined for their response to activated CD4⁺ T-cell help. A and B are associated to Figure 2, C and D are associated to Figure 3.

(**A**) Gating strategy for sorting the four progenitor-derived DC populations.

(**B**) Representative histograms of CD40, CD86, CCR7 and PD-L1 on the corresponding sorted progenitor-derived DC populations under the indicated conditions.

(**C**) Gating strategy for sorting progenitor-derived DC used in the NanoString nCounter analysis

(**D**) Single-sample gene set enrichment analysis (ssGSEA) was used to assess pathway activity. Violin plots show the distribution of ssGSEA scores for the class I MHC antigen presentation pathway and the ubiquitination/proteasome degradation pathway in progenitor-derived DC co-cultured with activated (a)CD4⁺ and naïve (n)CD4⁺ T cells. Each dot represents one sample.

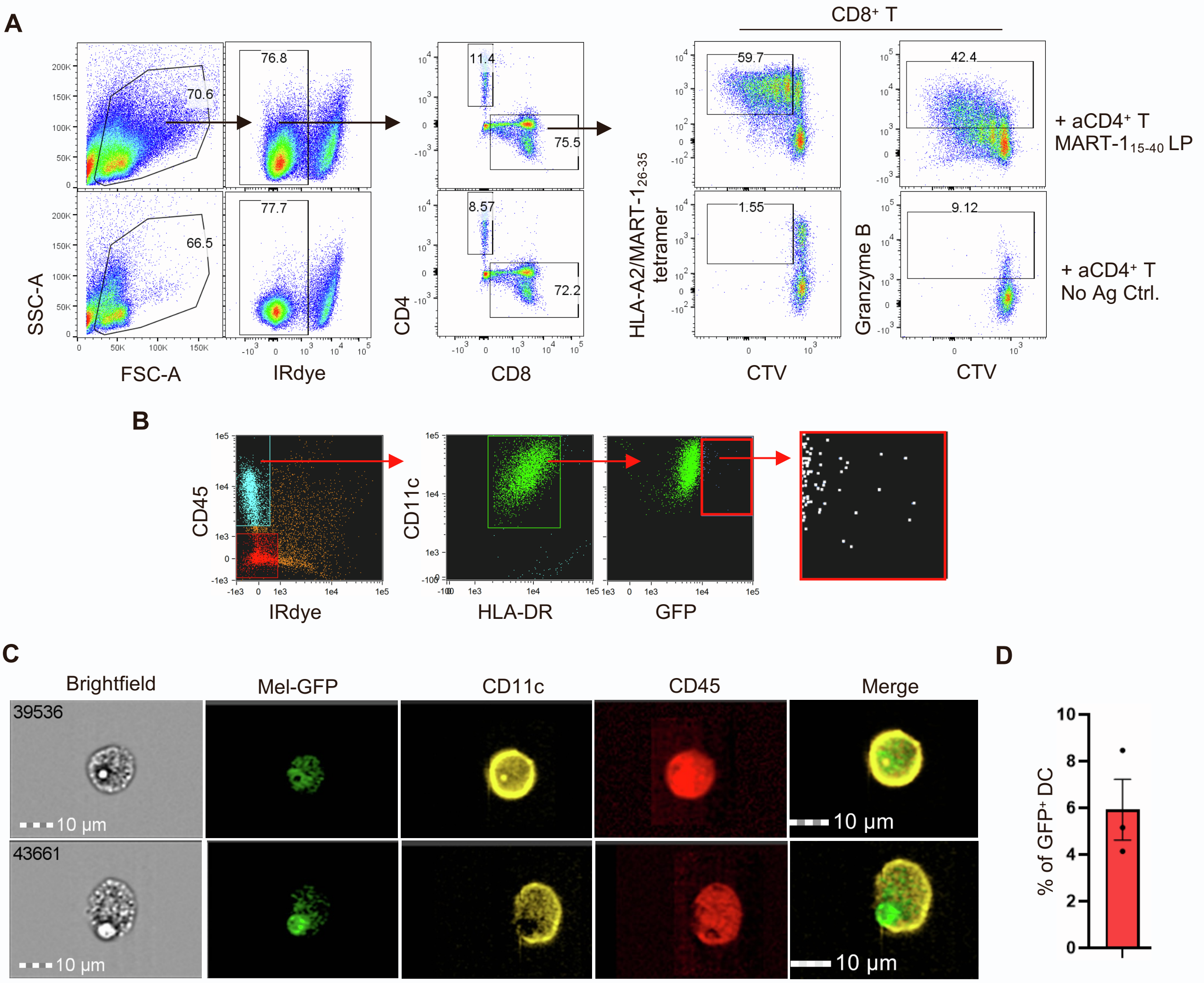

Figure S5. Progenitor-derived DC have the ability to relay CD4⁺ T-cell help for CTL responses, **related to Figure 4.**
(**A**) Gating strategies for detecting CD8⁺ T-cell responses in our *in vitro* cytotoxic T-cell priming system based on CTV dilution and intracellular Granzyme B staining. CD8⁺ T cells had been transduced to express the HLA-A2/MART-$1_{26\text{-}35}$-specific TCR before being added to priming system.
(**B-D**) Imaging flow cytometry was performed to analyze the phagocytotic capacity of progenitor-derived DC.
(**B**) Gating strategy for analyzing phagocytotic capacity of progenitor-derived DC response.
(**C**) Representative brightfield and fluorescent images of CD11c⁺CD45⁺ progenitor-derived DC phagocytosing GFP⁺ melanoma cell debris.
(**D**) Quantification of % of GFP⁺ cells in total live DC. Data are pooled from three (n=3) independent donors in D, and are shown as means ± SEM.

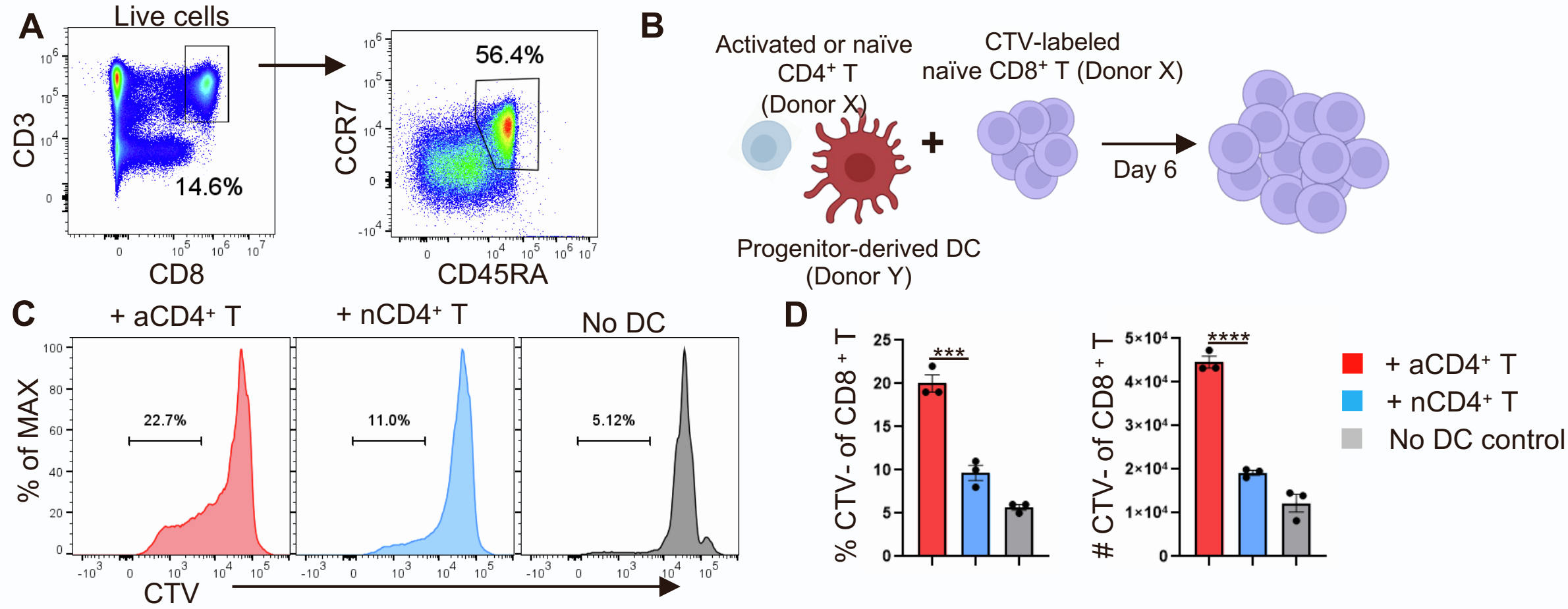

Figure S6. Progenitor-derived DC prime naïve CD8⁺ T cells in an allogeneic MLR, related to Figure 5.
The capacity of progenitor-derived DC to prime naïve CD8⁺ T cells was assessed using an allogeneic mixed lymphocyte reaction. Approximately 10,000 progenitor-derived DC from donors Y1-Y3 (n=3) were co-cultured with an equal number of allogeneic activated (a) or naïve (n) CD4⁺ T cells from donors X1-X3 (n=3), together with ~100,000 naïve CD8⁺ T cells from the same donors (X1-X3), to evaluate the effect of CD4⁺ T cell help on naïve CD8⁺ T-cell priming.
(**A**) Gating strategy for sorting naïve CD8⁺ T cells.
(**B**) Schematic illustration of the MLR experimental design.
(**C**) Representative CTV histograms showing proliferation of naïve CD8⁺ T cells under indicated conditions.
(**D**) Frequency (%) and absolute number (#) of CTV⁻ CD8⁺ T cells among total CD8⁺ T cells. Data are pooled from three (n=3) independent donors in **D** and shown as means ± SEM. ***p<0.001, ****p<0.0001(One-way ANOVA with Tukey's multiple comparisons).

A

Peptide ID	*Peptide sequence*	*HLA allele*	*Fluorochrome*
MART-1$_{25\text{-}36}$	ELAGYGILTV	HLA-A0201	AF488 & APC
CDK4$_{23\text{-}32}$	ALDPHSGHFV	HLA-A0201	APC & PE
MAGE3	FLWGPRALV	HLA-A0201	PE

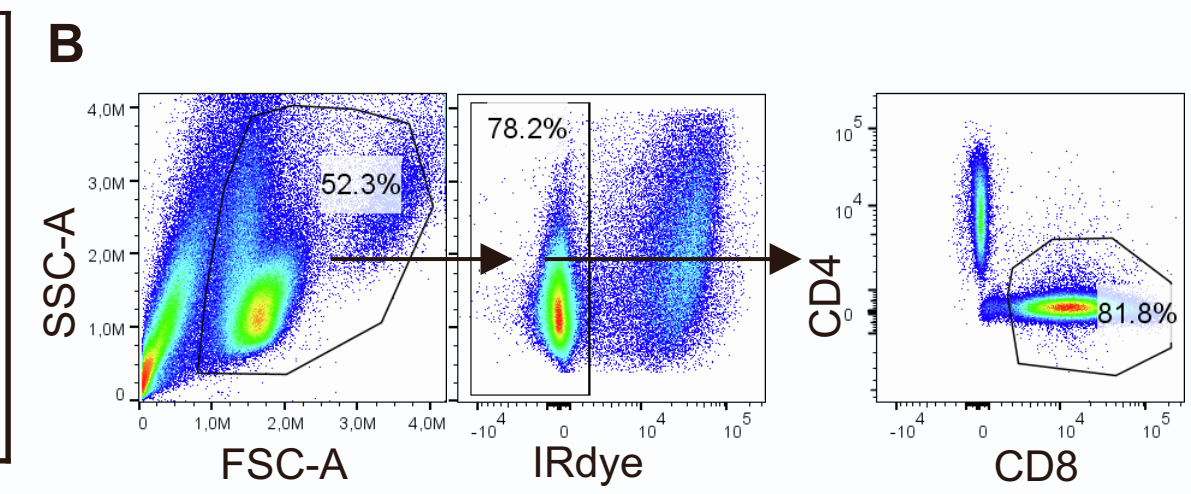

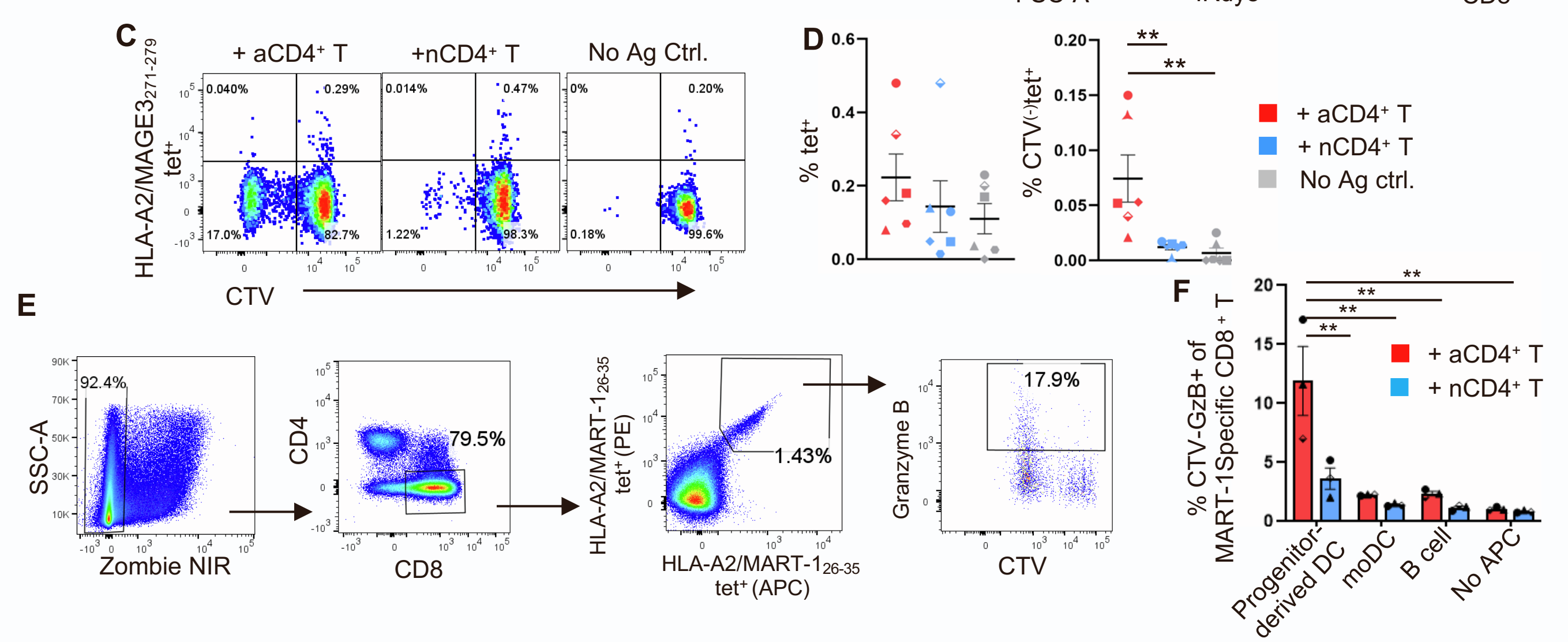

Figure S7. Helped progenitor-derived DC efficiently detect tumor antigen-specific CD8⁺ T cells in blood and outperform moDC and B cells, related to Figure 5 and 6.
(**A**) Overview of the tetramers used in the experiment(s).
(**B**) Gating strategy for detecting tumor antigen-specific CD8⁺ T cells using tetramers.
(**C**) Flow cytometric plots depicting CD8⁺ T cells specific for HLA-A2/MAGE3$_{271\text{-}279}$.
(**D**) Quantification % of MAGE3$_{271\text{-}279}$-specific population within CD8⁺ T cells (left), and # of MAGE3$_{271\text{-}279}$-specific CD8⁺ T cells (right).
(E-F) Progenitor-derived DC loaded with dead Mel526 cell debris were used to detect endogenous MAGE3$_{271\text{-}279}$-specific CD8⁺ T cells from blood in the presence of activated (a) or naïve (n) CD4⁺ T cells. Next, progenitor-derived DC, moDC, or B cells from same donor were compared for their ability to detect MART$_{26\text{-}35}$-specific CD8⁺ T cells in blood samples.
(**E**) Gating strategies for detecting CD8⁺ T-cell responses in the co-culture system based on MART-1$_{26\text{-}35}$/HLA-A2 tetramers staining and intracellular Granzyme B staining.
(**F**) Frequency (%) of CTV⁽⁻⁾Granzyme B⁺ MART-1$_{26\text{-}35}$/HLA-A2 tetramer⁺ CD8⁺ T cells under the indicated conditions.
Data are pooled from six (n=6) healthy donors in D and pooled from three (n=3) independent donors in F. Data are shown as means ± SEM. *p<0.05, **p<0.01 by one-way ANOVA with Tukey's multiple comparisons.

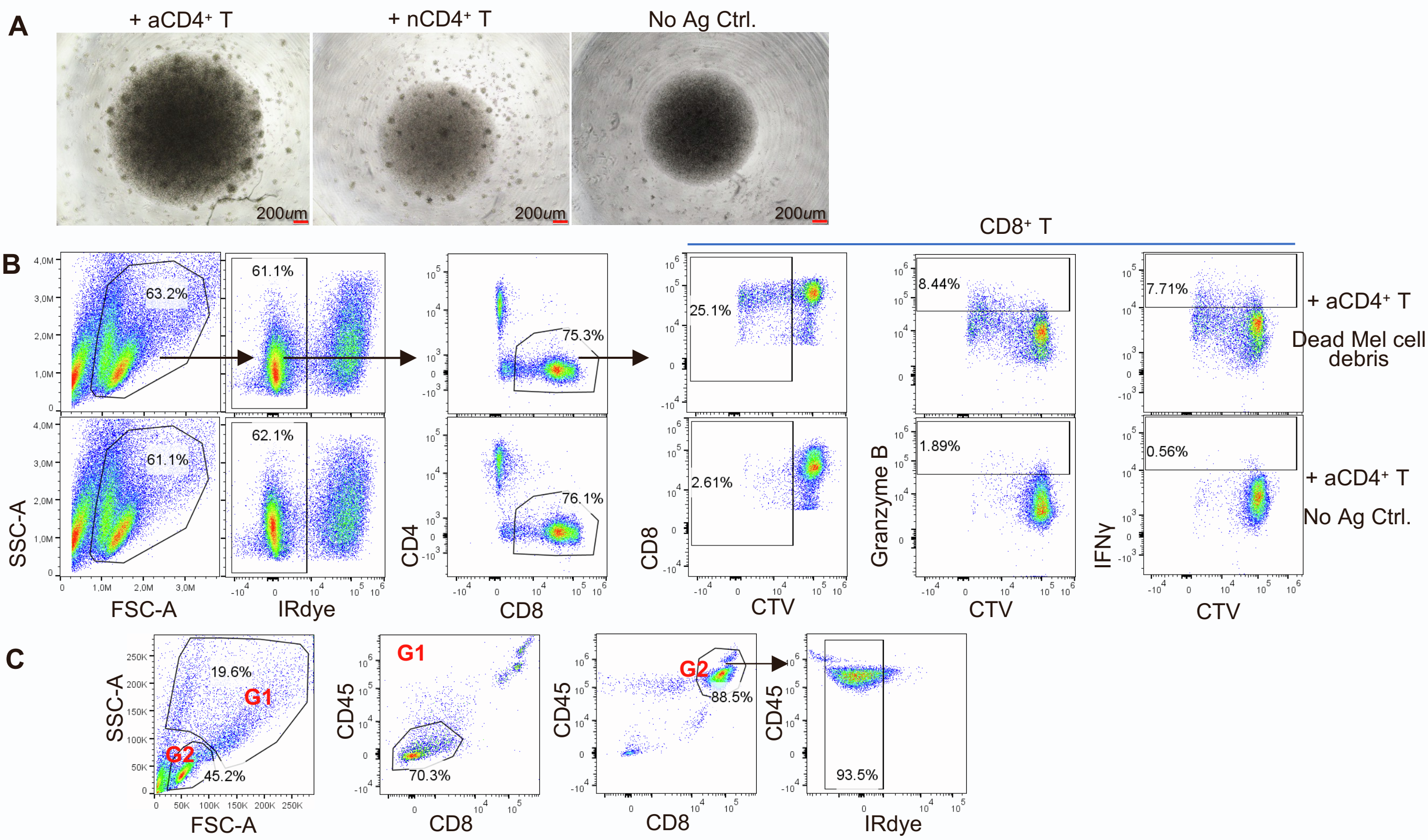

Figure S8. "Licensed/helped" progenitor-derived DC prime CTL that exhibit enhanced anti-tumor function, related to Figure 7.
CD8⁺ T cells were sorted from PBMC and co-cultured with progenitor-derived DC loaded with dead Mel526 cell debris in presence of activated (a)- or naïve (n)CD4⁺ T cells. Then CD8⁺ T-cell response was measured at day 6 after co-culture by flow cytometry or subjected to killing assay.
(**A**) Representative microscopic images depicting CD8⁺ T cells 6 days after co-culture.
(**B**) Gating strategies for detecting CD8⁺ T-cell responses in our *in vitro* cytotoxic T-cell priming system based on CTV dilution, and intracellular Granzyme B and IFNγ staining.
(**C**) Gating strategies for cells in suspension collected at the end of killing assay.

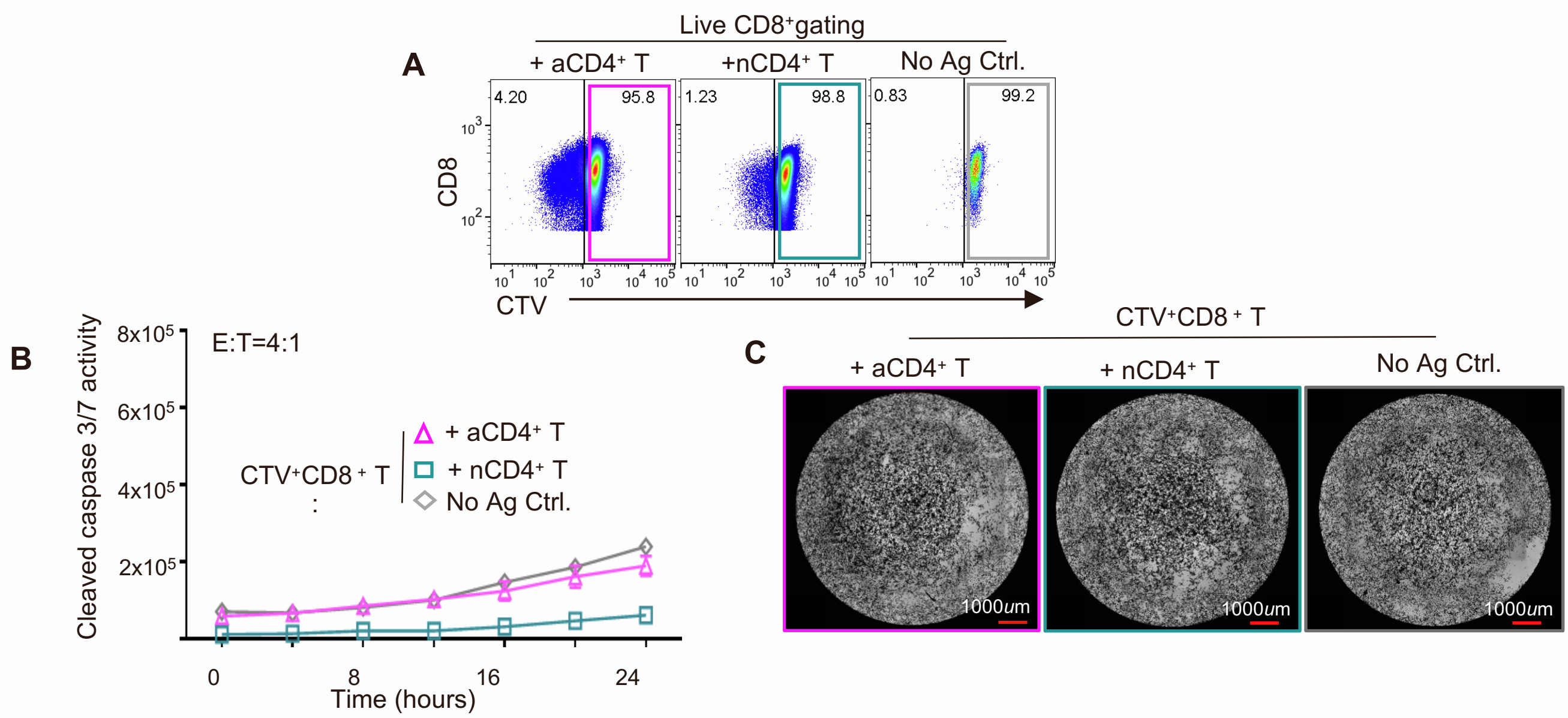

Figure S9. Non-proliferative CTV⁺CD8⁺ T cells have limited tumor cell killing capacity, related to Figure 7.
CD8⁺ T cells were sorted from PBMC and co-cultured with progenitor-derived DC loaded with dead Mel526 cell debris in presence of activated (a)- or naïve (n)CD4⁺ T cells. Then, killing assay was performed using non-proliferative (CTV⁺) CD8⁺ T cells purified from the priming system and Mel526 tumor cells at effector/target (E/T) ratio of 4/1, and analyzed by IncuCyte during 24h. At the end of the assay, remaining Mel526 cells in each well from all conditions were fixed and stained with crystal violet.
(**A**) Gating strategy for isolating CTV⁺CD8⁺ T cells from the CTL priming system. CTV⁺CD8⁺ T cells without encountering antigen were used as control. Purple, teal and grey boxes highlight isolated T-cell population.
(**B**) Cleaved caspase 3/7 activity in tumor cells during killing assay under indicated conditions.
(**C**) Representative CCD microscopic images depicting remaining Mel526 cells in the plate at 24h post co-culture under indicated conditions. Data are pooled from two (n=2) independent donors, are shown as means ± SD.

SUPPLEMENTAL REFERENCES

1. Balan, S., Arnold-Schrauf, C., Abbas, A., Couespel, N., Savoret, J., Imperatore, F., Villani, A.C., Manh, T.P.V., Bhardwaj, N., and Dalod, M. (2018). Large-Scale Human Dendritic Cell Differentiation Revealing Notch-Dependent Lineage Bifurcation and Heterogeneity. Cell Reports 24, 1902-1915.e6. https://doi.org/10.1016/j.celrep.2018.07.033.

2. Lei, X., Khatri, I., Wit, T. de, Rink, I. de, Nieuwland, M., Kerkhoven, R., Eenennaam, H. van, Sun, C., Garg, A.D., Borst, J., et al. (2023). CD4+ helper T cells endow cDC1 with cancer-impeding functions in the human tumor micro-environment. Nature Communications 14. https://doi.org/10.1038/s41467-022-35615-5.
