# Supplementary material for: Efficient generation of human cDC1-like cells to detect and enhance tumor-reactive T cells via CD4+ T cell help
Source: Cell Rep Methods. 2026 Jul 22;6(9):101533. doi: 10.1016/j.crmeth.2026.101533 (PMC13615486; doi:10.1016/j.crmeth.2026.101533)
Supplement: Supplementary file 2 [file mmc7.pdf]

Cell Reports Methods

Article

# Efficient generation of human cDC1-like cells to detect and enhance tumor-reactive T cells via CD4$^+$ T cell help

## Graphical abstract

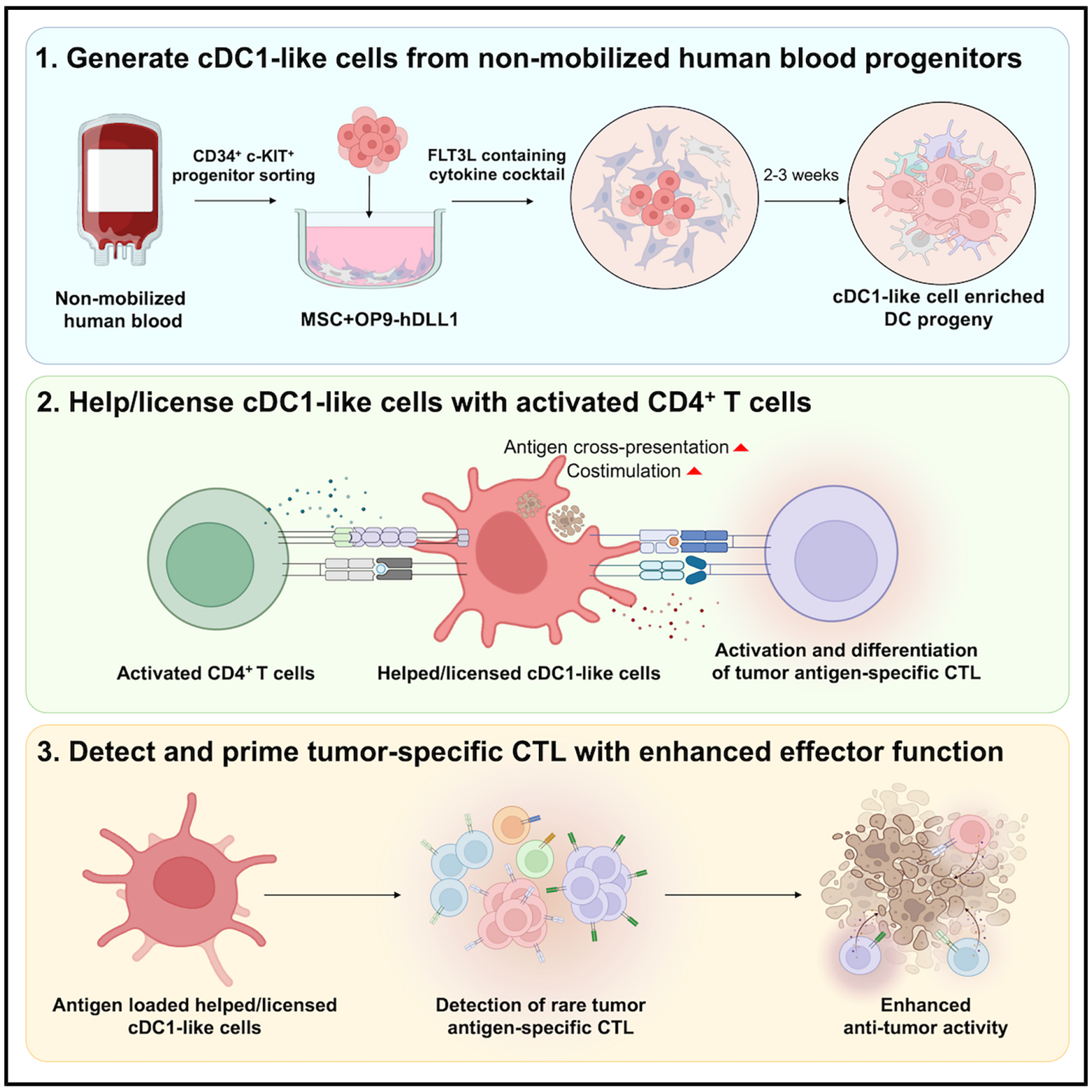


## Highlights

- Generation of cDC1-like cells from progenitors in non-mobilized human blood
- CD4$^+$ T cell-mediated licensing of cDC1-like cells improves CTL priming
- Antigen-loaded, helped/licensed cDC1-like cells detect rare tumor-specific CD8$^+$ T cells
- Antigen-loaded, helped/licensed cDC1-like cells improve effective CTL differentiation


## Authors

Yizhi Wang (汪逸之), Xin Lei (雷馨), Tom de Wit, ..., Chong Sun (孙崇), Jannie Borst, Yanling Xiao (肖艳灵)

## Correspondence

y.xiao@lumc.nl



## In brief

Wang et al. describe a methodology to generate cDC1-like cells from hematopoietic progenitors in non-mobilized human blood. These cDC1-like cells can be licensed by activated CD4$^+$ T cells to detect rare tumor-specific CD8$^+$ T cells in blood and prime functional tumor-reactive cytotoxic T cells. This platform supports immune monitoring and cell therapies in cancer.


Wang et al., 2026, Cell Reports Methods *6*, 101533
September 21, 2026 © 2026 The Author(s). Published by Elsevier Inc.
https://doi.org/10.1016/j.crmeth.2026.101533

CellPress

Cell Reports Methods

CellPress OPEN ACCESS

Article

# Efficient generation of human cDC1-like cells to detect and enhance tumor-reactive T cells via CD4$^+$ T cell help

Yizhi Wang (汪逸之),[1] Xin Lei (雷馨),[1,6] Tom de Wit,[1] Ellen Schrama,[1] Marij J.P. Welters,[2,6] Karel van Leeuwen,[1] Sander de Kivit,[1,6] Miriam de Boer,[1] Jitske van den Bulk,[3] Noel F.C. C. de Miranda,[3] Ton N. Schumacher,[4,6] Chong Sun (孙崇),[5] Jannie Borst,[1,6] and Yanling Xiao (肖艳灵)[1,7,*]

[1]Department of Immunology, Leiden University Medical Center, Leiden, the Netherlands
[2]Department of Medical Oncology, Leiden University Medical Center, Leiden, the Netherlands
[3]Department of Pathology, Leiden University Medical Center, Leiden, the Netherlands
[4]Department of Molecular Oncology and Immunology, the Netherlands Cancer Institute, Amsterdam, the Netherlands
[5]Immune Regulation in Cancer, German Cancer Research Center, Heidelberg, Germany
[6]Oncode Institute, Utrecht, the Netherlands
[7]Lead contact
*Correspondence: y.xiao@lumc.nl
https://doi.org/10.1016/j.crmeth.2026.101533

**MOTIVATION** Classical type 1 dendritic cells (cDC1s) are specialized in cross-presenting tumor antigens and relaying CD4$^+$ T cell help to prime CD8$^+$ T cells, yet their scarcity in peripheral blood limits their practical use. We have developed a method to generate cDC1-like cells from hematopoietic progenitors as present in non-mobilized adult human blood. Once helped/licensed by activated CD4$^+$ T cells, tumor antigen-loaded helped cDC1-like cells can identify and expand rare tumor antigen-specific CD8$^+$ T cells from blood and induce their differentiation into cytotoxic T lymphocytes. This cDC1/T cell co-culture platform thereby improves detection and isolation of tumor-specific CD8$^+$ T cells to serve immunomonitoring and adoptive T cell therapies in cancer.

## SUMMARY

Classical type 1 dendritic cells (cDC1s) are crucial to anti-tumor immunity by cross-presenting tumor antigens and priming cytotoxic CD8$^+$ T lymphocytes (CTLs). Licensing via CD4$^+$ T cell help endows cDC1s with enhanced cross-presentation and CTL-priming capacity, making them a promising tool to improve adoptive T cell therapies. However, the scarcity of primary human cDC1s limits their in-depth translational investigation and application. Here, we describe a method to efficiently generate DCs *in vitro* from CD34$^+$c-KIT$^+$ progenitors in non-mobilized peripheral blood. This DC population is enriched for cDC1-like cells that respond to CD4$^+$ T cell help by upregulation of key molecules involved in antigen cross-presentation and T cell costimulation. Upon CD4$^+$ T cell-mediated licensing, the progenitor-derived DC promotes tumor-specific CTL priming and facilitates detection of rare tumor antigen-specific CD8$^+$ T cells in blood and tumor tissues. This DC culture platform incorporating CD4$^+$ T cell help provides a scalable system for immunomonitoring and optimizing adoptive T cell therapies in cancer.

## INTRODUCTION

Dendritic cells (DCs) are antigen-presenting cells that act as immune sentinels.[1] They patrol tissues, phagocytose debris, and detect danger signals via pattern recognition receptors (PRRs).[2] They migrate to lymph nodes (LNs) to induce either T cell tolerance or immunity. The DC lineage includes plasmacytoid DC (pDC) and classical DC (cDC) subsets,[3,4] with cDCs further divided into cDC1 and cDC2[5] that differ from monocyte-derived DCs (moDCs), which arise under inflammatory conditions.[3,6,7] cDCs develop under homeostatic conditions in the bone marrow (BM) from hematopoietic progenitor cells (HPCs), driven by FLT3 receptor signaling.[8] Subset development is regulated by transcription factor networks, ZEB2 for pDCs, IRF8 and BATF3 for cDC1s, and IRF4 for cDC2s and moDCs.[4,9,10] DC subsets are also distinguished by key markers: CD123 and CD303 for pDCs, CD141 and XCR1 for cDC1s, CD1c and SIRPα for cDC2s, and CD14 and CD1a for moDCs.[4,11]


© 2026 The Author(s). Published by Elsevier Inc.
This is an open access article under the CC BY license (http://creativecommons.org/licenses/by/4.0/).

Among DC lineages, cDCs uniquely transport antigens from peripheral tissues to draining (d)LNs to initiate T cell activation.[12,13] Specifically, cDC1s excel in cross-presenting cell-associated antigens on major histocompatibility complex (MHC) class I to activate $CD8^+$ T cells.[14] In tumors, cDC1s can maintain a reservoir of primed, stem-like $CD8^+$ T cells in the tumor draining (td)LN[15] and shape T cell states in the tumor microenvironment (TME).[16–19] A key feature of cDC1s is their ability to interact with and respond to activated $CD4^+$ T cells, a process known as DC licensing, which enhances their survival, maturation, migration, and antigen (cross)-presentation abilities.[19,20] $CD4^+$ T cell help promotes human cDC1 maturation and endows cDC1s with optimal cross-presentation ability, leading to superior priming of cytotoxic T lymphocytes (CTLs) against tumor-associated antigens as compared to cDC1s matured by PRR stimuli. The response to activated $CD4^+$ T cells is specific for cDC1s and not displayed by primary cDC2s, pDCs, or moDCs.[19] Activated $CD4^+$ T cells license cDC1s via both CD40 ligand (L) and IFN-I,[20–22] with the latter specifically promoting antigen cross-presentation features. cDC1 licensing by $CD4^+$ T cells is required for optimal primary CTL responses to cancer, as shown by intervention studies in mouse models[20,23] and by correlative evidence in humans.[19,22] Also in infection, where PRR stimuli are abundant, cDC1 licensing by $CD4^+$ T cells is required for CTL-based immunity.[24] $CD4^+$ T cell help promotes clonal expansion and effector and effector-memory differentiation of $CD8^+$ T cells, making them highly functional CTLs.[25,26] Through these mechanisms, cDC1s play a pivotal role in CTL-mediated anti-tumor immunity[27–29] and are attractive targets or tools in cancer immunotherapy.

While immune checkpoint blockade, primarily programmed cell death protein 1 (PD-1) or programmed death-ligand 1 (PD-L1) inhibition, has achieved clinical success, its efficacy is largely limited to tumors already infiltrated by tumor-specific CTLs.[30–33] For immunologically "cold" or non-immunogenic tumors, adoptive transfer therapy (adoptive cell therapy [ACT]) offers promise, via tumor-infiltrating lymphocytes (TILs)[34] or genetically engineered T cells expressing tumor-specific receptors.[35] Tumor-specific $CD8^+$ T cells can now be identified using MHC class I multimers labeled with fluorophores[36] or metals,[37] phenotypic markers such as PD-1[38] and CD39,[37] functional assays measuring secretion of cytokines like IFN-$\gamma$,[39] or through screening of TCRs.[40,41] Although TILs are a rich source of tumor-reactive T cells, they are not readily available for all patients, and expanding them to clinically relevant numbers remains laborious and is not always successful. Circulating tumor-specific T cells offer an alternative, but their low frequency poses a major challenge.[42–44] Additionally, resistance to ACT may result from impairment of T cell function or antigen recognition.[45–47] This highlights the need for improved methods to detect tumor-specific T cells in blood and to enhance the functionality of therapeutic T cell products. In this context, cDC1s hold strong potential, both as tools to identify rare tumor-specific T cells and to increase their number and improve their function *in vivo*.

However, clinical application of cDC1s is limited by their scarcity, constituting only ~0.02% of peripheral blood cells[19] and showing substantial donor variability. A promising alternative is to generate cDC1-like cells *in vitro* from HPCs, which in current protocols is achieved by combining FTL3 ligand with delta-like ligand 1 (DLL1) that induces Notch signaling and improves murine[48] and human[49] cDC1 differentiation from progenitors. As HPC sources, cord blood[49] and mobilized peripheral blood[50] are used, which may be impractical for routine clinical application.

Here, we present an optimized method to generate cDC1-like cells in high yield from $CD34^+$c-$KIT^+$ HPCs, as isolated from non-mobilized adult blood. The *in-vitro*-generated progenitor-derived DC population is highly enriched for cDC1-like cells that respond to $CD4^+$ T cell help as do primary cDC1s. The helped/licensed progenitor-derived DC population can improve effector responses of tumor-specific CTLs and enable sensitive detection of rare tumor antigen-specific $CD8^+$ T cells in healthy human blood. We hereby offer a new methodology to improve personalized CTL-based immunotherapy.

## RESULTS

### *In vitro* generation of human cDC1-like cells from adult blood-derived progenitors

To facilitate implementation, we aimed to use non-mobilized adult blood as a source of $CD34^+cKIT^+$ HPCs to generate cDC1s. Flow cytometric sorting (Figure 1A) yielded ~15,000 progenitors per $10^8$ peripheral blood mononuclear cells (PBMCs) (Figure 1B). The progenitors failed to expand during the cytokine-driven phase when we used the protocol of Kirkling et al.[47] for DC generation (method 1; Figures S1A and S1B). Directly culturing $CD34^+$c-$KIT^+$ HPCs on different feeder layers (Figures S1A and S1B) with a cytokine cocktail of FLT3L, SCF, TPO, IL-7, GM-CSF, and M-CSF (FST7GM), based on Balan et al.[49] and our previous work,[51–53] yielded significantly more live cell progeny (methods 2 and 3; Figure S1C). As feeders, we first used OP9 cells of which 30% expressed human DLL1 (hDLL1) to engage Notch signaling and support cDC1 differentiation[49] (method 2). We next used OP9-hDLL1 cells in combination with human BM-derived mesenchymal stromal cells (MSCs) to support HPC growth[51–55] (method 3). This led to a 10-fold increase in live cell yield (Figure S1C) and a significantly higher frequency and absolute number of cells with general DC markers ($CD11c^+HLA\text{-}DR^+$) and cDC1 markers (XCR1 and CLEC9A) (Figures S1D–S1F). Using method 3, progenitors expanded ~100-fold (Figures 1C–1E), yielding ~60% DCs with a $CD3^-CD19^-HLA\text{-}DR^+CD88^-$ phenotype, wherein CD88 is a marker associated with moDCs[56] (Figures S2A and S2B).

To assess the lineage composition of the progenitor-derived DC culture, we analyzed it by spectral flow cytometry for many relevant markers. The culture was clearly heterogeneous and resolved into ten clusters with discernible phenotypes (Figure 1F; Figure S2C). Both CD123 and CD303 were not detected, indicating that pDCs were absent among the progenitor-derived DCs (Figure S2D). Quantitative analysis of all markers by OMIQ identified clusters C1, C3, C6, and C9 as cDC1-like cells, on the basis of expression of CLEC9A and IRF8, as well as the lack of SIRP$\alpha$ (Figure 1G; Figure S2D). Notably, CD141, a classical marker of *ex vivo* cDC1,[4,11] was highly expressed on cDC1-like cells in cluster 9 but also on the $CD14^+$ monocytic cluster 10 (Figure 1G). Up to 60% of the population consisted of cDC1-like cells (Figure 1H; Figure S2C). The other clusters

CellPress
OPEN ACCESS

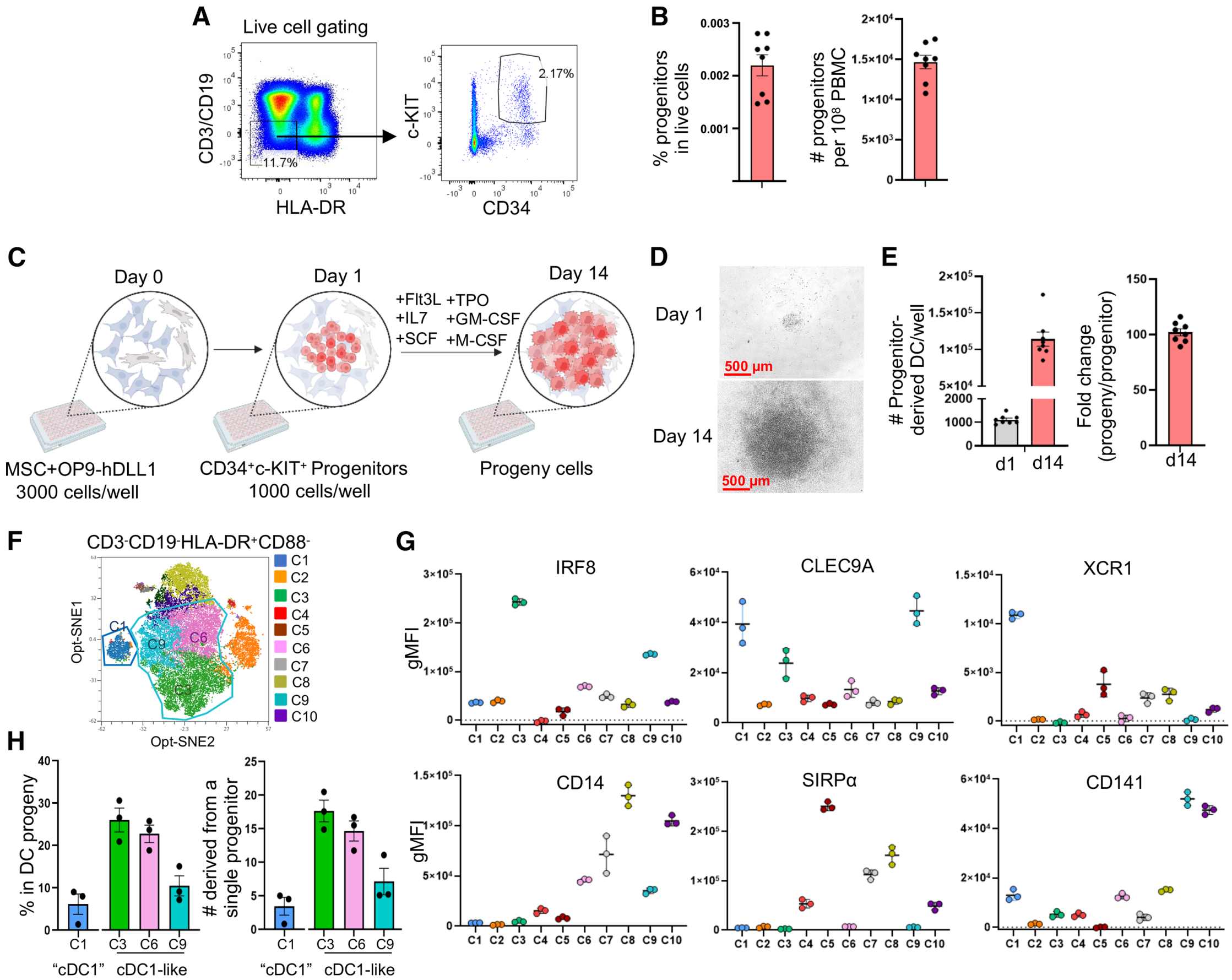


**Figure 1. Generating human cDC1-like cells *in vitro* from progenitors in non-mobilized blood of healthy adult donors**

CD34+c-KIT+ progenitors were flow cytometrically sorted from PBMCs and co-cultured for 14–21 days at ∼1,000 cells per well with −3,000 feeder cells (70% hMSC + 30% hDLL1+OP9 cells) in the presence of FLT3L (100 ng/mL), TPO/SCF/GM-CSF (each 5 ng/mL), IL-7 (1 ng/mL), and M-CSF (0.5 ng/mL).

(A) Gating strategy for progenitor cell isolation from PBMCs.

(B) Frequency (%) of progenitors among live PBMCs and absolute numbers (#) of progenitors isolated from 100 × $10^6$ PBMCs.

(C) Illustration of the culture system for *in vitro* DC differentiation from progenitors.

(D) Microscopic pictures depicting outgrowth of cells from progenitors.

(E) Absolute numbers (#) of progenitors on day 1 and progeny cells on day 14, and the fold change of progeny versus progenitor cells.

(F) Opt-SNE plot of 10 clusters identified within CD3−CD19−HLA-DR+CD88− progeny cells. Cluster 1 (C1) outlined in blue denotes cells with cDC1 phenotype, based on XCR1, CD141, CLEC9A, and IRF8. Combined C3, C6, and C9 outlined in light blue denote cells with cDC1-like phenotype.

(G) gMFI quantifications for IRF8, CLEC9A, XCR1, CD14, SIRPα, and CD141 across the 10 progenitor-derived DC clusters.

(H) Frequencies (%) of C1, C3, C6, and C9 in HLA-DR+CD88− DC progeny and absolute numbers (#) of C1, C3, C6, and C9 calculated per input progenitor.

Data are pooled from eight (*n* = 8) independent donors in (B) and (E); data are pooled from three independent donors (*n* = 3) in (G) and (H). Data are shown as means ± standard error of mean (SEM). See also Figure S2.

contained cells with cDC2 features and monocytic features (Figure 1G; Figures S2D and S2E). Manual gating also discerned CD14−CLEC9A+XCR1+ cDC1-like cells, CD14−/+IRF8+SIRPα− cDC1-like cells, and CD14+IRF8−SIRPα+ cDC2-like/monocytic cells (Figure S2F). To assess the maturity of the cDC-like populations in our culture, we compared them to pre-DC phenotype cells in peripheral blood that express AXL, SIGLEG6, and FLT3 and low levels of CD45RA.[7,57] Unlike *ex vivo* AXL+SIGLEC6+ cells in peripheral blood, progenitor-derived CLEC9A+ and IRF8+SIRPα− cDC1-like cells as well as IRF8−SIRPα+ cDC2-like/monocytic cells lacked AXL and SIGLEC6 expression, while retaining FLT3 and exhibiting variable levels of CD45RA (Figures S2G–S2I). These data argue that the DC population obtained from the progenitors was of variable maturity, differentiated beyond the pre-DC state. Based on the total input of progenitors and final cDC1-like cell counts (C1, C3, C6, and C9), this corresponds to an average yield of approximately 45 cDC1-like cells per input progenitor (Figure 1H).

## Response of progenitor-derived cDC1-like cells to activated CD4+ T cells

We previously demonstrated that *ex vivo* human cDC1s are unique as compared to cDC2s, pDCs, and moDCs in responding to activated CD4+ T cells by upregulating multiple pathways important for antigen cross-presentation and CTL priming.[19] In that *in vitro* setup, we mimicked cDC1 licensing by CD4+

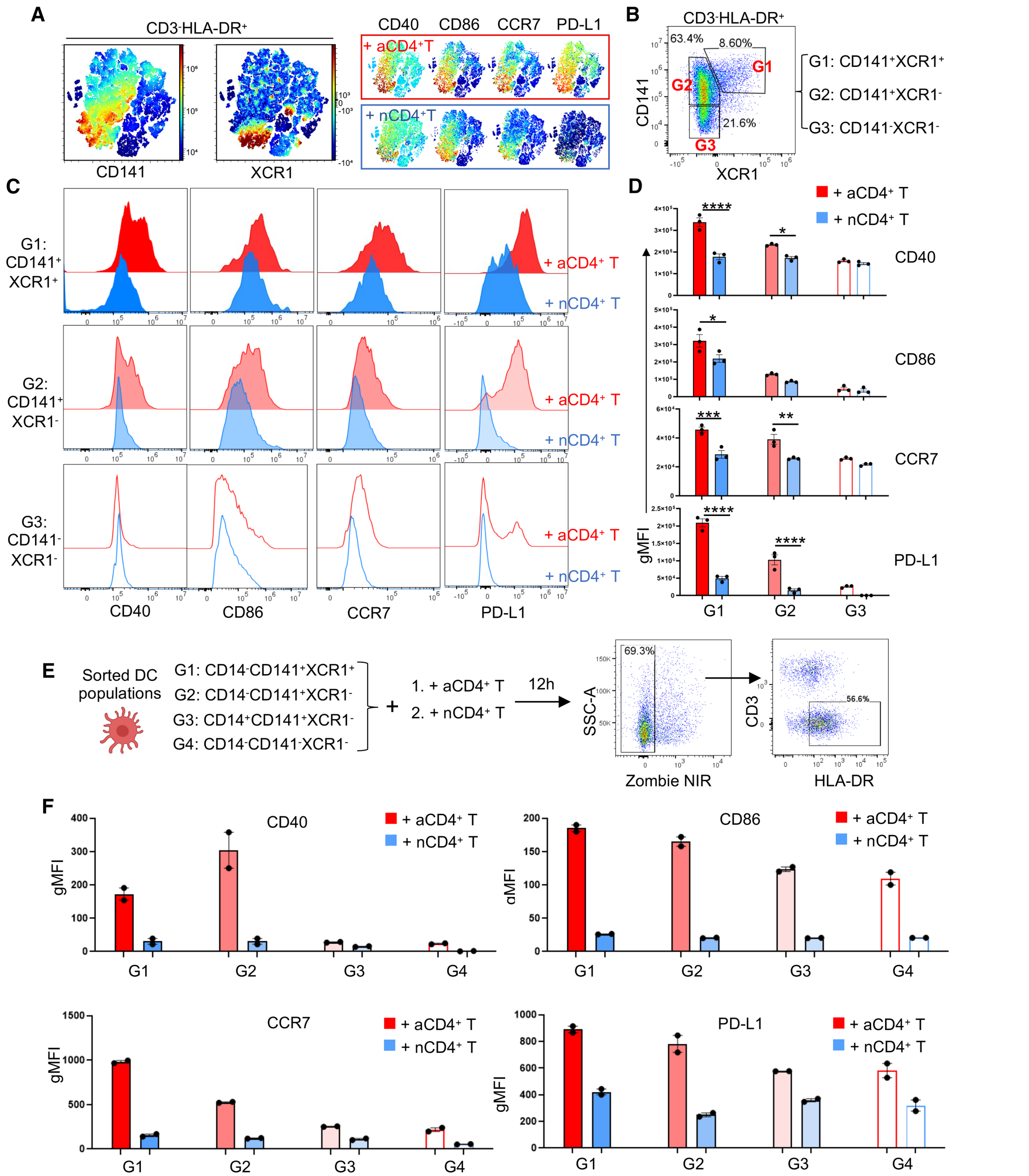

**Figure 2. Response of progenitor-derived cDC1-like cells to activated CD4$^+$ T cells**

Progenitor-derived DCs were co-cultured with activated (a) or naive (n) CD4$^+$ T cells overnight. Next, key molecules of the cDC1 "help" signature[19] were analyzed by flow cytometry.

(A) Opt-SNE plots (left) showing clusters of progenitor-derived DCs incorporating both culture conditions (+nCD4$^+$ T and + aCD4$^+$ T), colored by CD141 or XCR1 expression. Opt-SNE plots (right) showing CD40, CD86, CCR7, and PD-L1 expression on progenitor-derived DCs after co-culture with either aCD4$^+$ or nCD4$^+$ T cells.

(B) Gating strategy for defining 3 subpopulations within CD3$^-$HLA-DR$^+$ progenitor-derived DC population after co-culture with either aCD4$^+$ or nCD4$^+$ T cells, based on XCR1 and CD141 expression.

(C) Representative histogram showing expression of the indicated markers in the 3 progenitor-derived DC subpopulations under indicated conditions.

(D) Quantification of gMFI comparing expression levels of the indicated markers across the 3 progenitor-derived DC subpopulations after co-culture with aCD4$^+$ or nCD4$^+$ T cells.

(legend continued on next page)

T cells by activating $CD4^+$ T cells with agonistic anti-CD3 monoclonal antibodies (mAb) instead of letting them recognize specific antigen, as occurs physiologically. We here tested, in the same way, whether the progenitor-derived DC population responded to activated $CD4^+$ T cells by reading out the same costimulatory molecules, chemokines, and antigen-presenting molecules. Despite the heterogeneity of the gated $CD3^-HLA\text{-}DR^+$ population, various DC licensing markers[19] were significantly upregulated in response to activated, but not naive, $CD4^+$ T cells, including CD70, CD80, CD83, PD-L1, chemokines CXCL9/10, as well as MHC class I and II (Figures S3A–S3F). Comparison of the response of the progenitor-derived-DCs to activated $CD4^+$ T cells versus PRR stimulation showed that stimulation with activated $CD4^+$ T cells led to significantly higher expression of various costimulatory molecules and of CXCL9/10 (Figures S3H–S3K).

To assess whether cDC1-like cells were the responders to the activated $CD4^+$ T cells, we further zoomed in on the $CD3^-HLA\text{-}DR^+$ population tested in Figures S3A–S3F, analyzing separately the response of gated $CD141^+XCR1^+$, $CD141^+XCR1^-$, and $CD141^-XCR1^-$ cells (Figures 2A and 2B). The most pronounced upregulation of cDC1 licensing markers CD40, CD86, CCR7, and PD-L1 in response to stimulation by activated versus naive $CD4^+$ T cells was observed in the $CD141^+XCR1^+$ population, followed to a lesser extent by the $CD141^+XCR1^-$ population, whereas the $CD141^-XCR1^-$ population showed minimal responses (Figures 2C and 2D). These results indicate that the response can primarily be attributed to the cDC1-like compartment within the heterogeneous DC population. To further substantiate these findings, we examined the response of flow cytometrically sorted subsets of the progenitor-derived DC population. Four test populations were sorted from $CD11c^+HLA\text{-}DR^+$ cells: $CD14^-CD141^+XCR1^+$ (cDC1-like), $CD14^-CD141^+XCR1^-$ (cDC1-like), $CD14^+CD141^+XCR1^-$ (cDC2-like and moDC-like), and $CD14^-CD141^-XCR1^-$ (undefined), which were stimulated with activated or naive $CD4^+$ T cells (Figure 2E; Figure S4A). Consistent with the gating-based analysis, the cDC1-like fractions showed the most pronounced and consistent upregulation of cDC1 licensing markers CD40, CD86, CCR7, and PD-L1 (Figure 2F; Figure S4B). These results indicate that cDC1-like cells are the dominant responders to $CD4^+$ T cell help, while other myeloid populations may contribute, but to a lesser extent.

Analysis of gene expression in the $CD3^-HLA\text{-}DR^+$ progenitor-derived DC population using the nCounter Host Immune Response Panel (Figure S4C; Table S1) revealed 70 differentially expressed genes (DEGs) between the two conditions (Figure S4C; Figure 3A; Table S2), including characteristic upregulation of CD70, CXCL9, and CXCL10[19] in response to activated $CD4^+$ T cells (Figure 3B). In fact, after culture with activated $CD4^+$ T cells, progenitor-derived DCs showed enrichment of the "help" gene signature we previously defined in primary *ex vivo* cDC1s[19] (Figure 3C; Tables S3 and S4), along with upregulation of IFN-$\alpha/\beta/\gamma$ and IL-12 cytokine signaling, as well as of the antigen presentation pathways (Figure 3D; Figure S4D). Collectively, these findings indicate that progenitor-derived DCs respond to activated $CD4^+$ T cells, with the response primarily driven by the cDC1-like population.

## Progenitor-derived DCs can relay $CD4^+$ T cell help for priming of (tumor) antigen-specific CTL

$CD4^+$ T cell help endows cDC1s with the optimal capacity to cross-prime CTLs in response to tumor cell debris.[19,24] We used our *in vitro* CTL-priming platform[19] to investigate whether progenitor-derived DCs could relay $CD4^+$ T cell help for CTL (cross)-priming. $CD8^+$ T cells from PBMCs were transduced with a TCR specific for the MART-$1_{26–35}$ peptide presented by HLA-A2. HLA-$A2^+$ HPCs were used to generate progenitor-derived DCs, which were cultured with naive or activated $CD4^+$ T cells, loaded with the MART-$1_{26–35}$ short peptide (SP), MART-$1_{15–40}$ long peptide (LP), or debris of MART-1-expressing Mel526 cells[19] in the presence of PRR stimuli, and then co-cultured with CellTrace Violet (CTV)-labeled $CD8^+$ T cells (Figure 4A). While SP can be loaded into MHC-I directly, LP and particularly cell debris require uptake and processing for cross-presentation. The $CD8^+$ T cell response was read out by CTV dilution and granzyme B production (Figure S5A). In the SP setting, progenitor-derived DCs induced robust MART-1-specific $CD8^+$ T cell proliferation (Figure 4B) and granzyme B production (Figure 4C), with a modest effect of activated $CD4^+$ T cells (Figures 4B and 4C). In the LP setting, CTL responses were significantly higher when DCs were "helped" by activated $CD4^+$ T cells (Figures 4D and 4E). In the Mel526 debris setting, progenitor-derived DCs phagocytosed $GFP^+$ melanoma cell debris, as shown by GFP, CD45, and CD11c co-expression via imaging flow cytometry (Figures S5B–S5D). Only helped DCs induced MART-$1_{26–35}$-specific $CD8^+$ T cell proliferation (Figure 4F) and granzyme B production (Figure 4G), whereas "non-helped" DCs failed to do so (Figures 4F and 4G). Interestingly, proliferation was also observed in HLA-A2/MART-$1_{26–35}$ tetramer-negative $CD8^+$ T cells, indicating that progenitor-derived DCs can prime CTLs against tumor antigens beyond MART-1 (Figure 4F), as demonstrated in our previous study.[19] Thus, similar to *ex vivo* cDC1s,[19] progenitor-derived DCs responded to $CD4^+$ T cell help by enhancing their CTL (cross)-priming capacity.

We also tested whether progenitor-derived DCs could prime naive $CD8^+$ T cells that recognized alloantigen with their endogenous TCR. For this purpose, naive $CD3^+CD8^+CD45RA^+CCR7^+$ $CD8^+$ T cells and $CD3^+CD4^+CD45RA^+CD25^-$ $CD4^+$ T cells were

(E and F) Responses of sorted progenitor-derived DCs to activated upon $CD4^+$ T cell.

(E) Schematic illustration of the experimental design. The four sorted populations were cultured either alone or together with activated $CD4^+$ T cells.

(F) Quantification of gMFI of the indicated markers on the corresponding sorted progenitor-derived DC populations under the indicated conditions.

Data in (D) are pooled from three independent donors ($n = 3$), and data in (F) are pooled from two independent donors ($n = 2$). Data are shown as means ± SEM in (D) and means ± standard deviation (SD) in (F). *$p < 0.05$, **$p < 0.01$, ***$p < 0.001$, ****$p < 0.0001$. Statistical analyses were performed using a two-way ANOVA with Tukey's multiple comparisons for (D). See also Figures S4A and S4B.

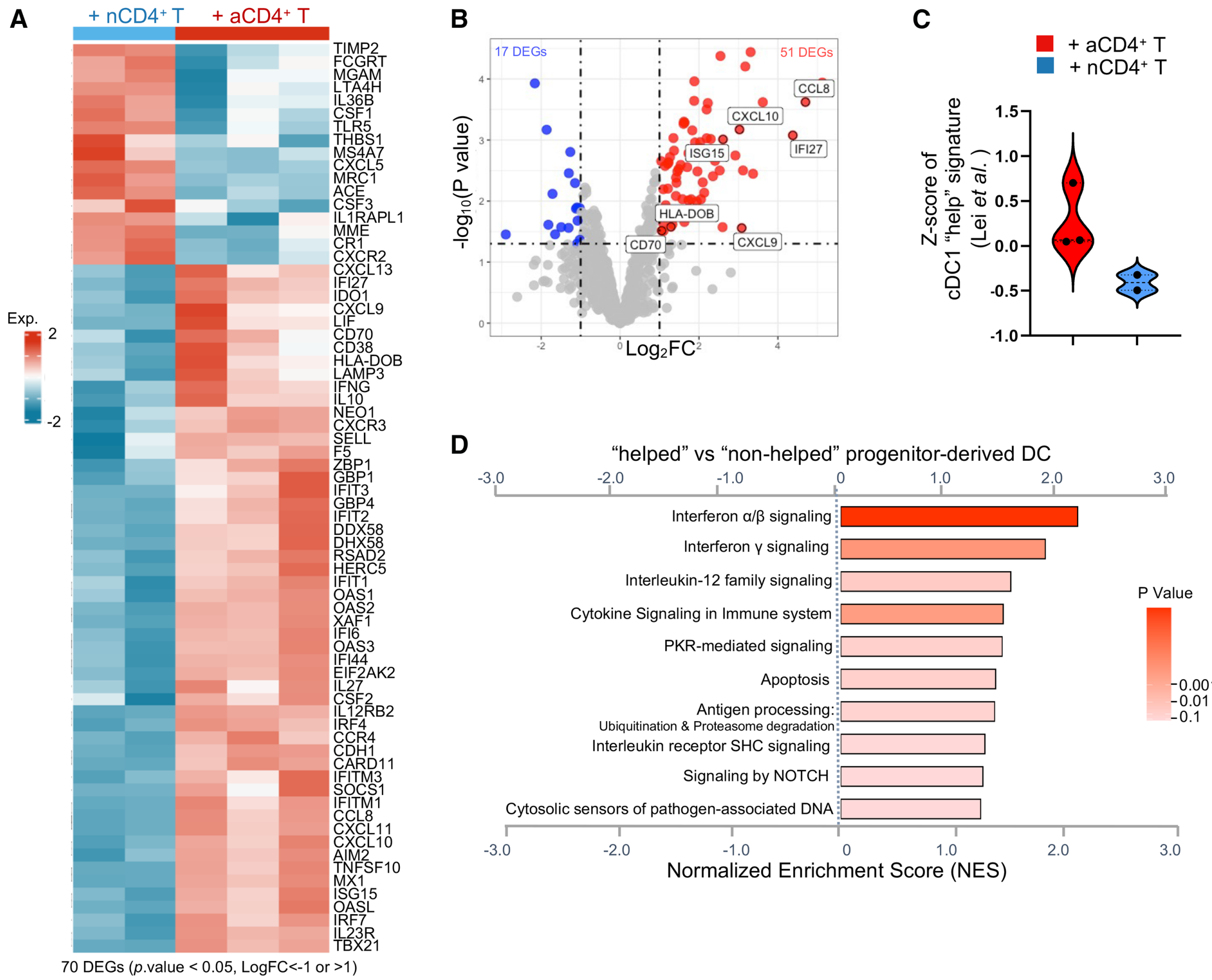


**Figure 3. Gene profiling by NanoString reveals defined licensing signature in progenitor-derived DCs**

Bulk progenitor-derived DCs were co-cultured with either activated (a) or naive (n) CD4+ T cells. Subsequently, HLA-DR+CD3− cells were sorted, lysed, and subjected to NanoString nCounter analysis.

(A) Heatmap displaying normalized and $log_2$-transformed transcript levels of differentially expressed genes (DEGs) ($p < 0.05$) across progenitor-derived DC samples co-cultured with either aCD4+ T cells ($n = 3$) or nCD4+ T cells ($n = 2$).

(B) Volcano plot depicting DEGs between progenitor-derived DCs co-cultured with aCD4+ or nCD4+ T cells. Genes shown in red or blue have $p < 0.05$ and $log_2$ fold change ($log_2$FC) > 1 in the respective conditions.

(C) $Z$ scores were calculated per sample by aggregating expression values of genes from a previously reported cDC1 "help" signature,[19] using the average of scaled (z-transformed) expression values. Violin plots depict the distribution of $Z$ scores between progenitor-derived DCs co-cultured with aCD4+ or nCD4+ T cells. Each dot represents one sample.

(D) Bar chart showing the top ten enriched Reactome pathways among genes upregulated in "helped" progenitor-derived DCs.

Pathways are ranked by normalized enrichment score (NES), and only positively enriched pathways (NES >0) are shown. See also Figures S4C and S4D.

isolated by flow cytometry from the same donors, while progenitor-derived DCs were derived from different, HLA-mismatched donors (Figure S6A). CD4+ T cells were activated with anti-CD3 mAb or not and co-cultured overnight, as in previous assays, with progenitor-derived DCs. Next, CTV-labeled naive CD8+ T cells were added and co-cultured with the HLA-mismatched progenitor-derived DCs for 6 days, after which CD8+ T cell proliferation was quantified based on CTV dilution (Figure S6B). Progenitor-derived DCs that had been co-cultured with naive CD4+ T cells could induce a response in naive CD8+ T cells as compared to the setting without DCs, but the response was greatly enhanced by activated CD4+ T cell help (Figures S6C and S6D). These data provide evidence that progenitor-derived DCs can prime naive CD8+ T cells that recognize alloantigen and that this capacity is improved upon their licensing by activated CD4+ T cells.

## Detection of tumor-specific CD8+ T cells in healthy donors with helped progenitor-derived DCs

We next tested whether our CTL-priming platform with progenitor-derived DCs could be used to detect tumor antigen-specific CD8+ T cells in healthy donor PBMCs. As proof of principle, we tested reactivity against three HLA-A2-restricted melanoma antigens (MART-1/CDK4/MAGE3) (Figure S7A), using debris from Mel526 cells expressing these antigens as the source. DCs and T cells from the same HLA-A2+ donors were co-cultured for 6 days, after which tumor antigen-specific CD8+ T cells were detected with peptide/MHC-I tetramers, in combination with

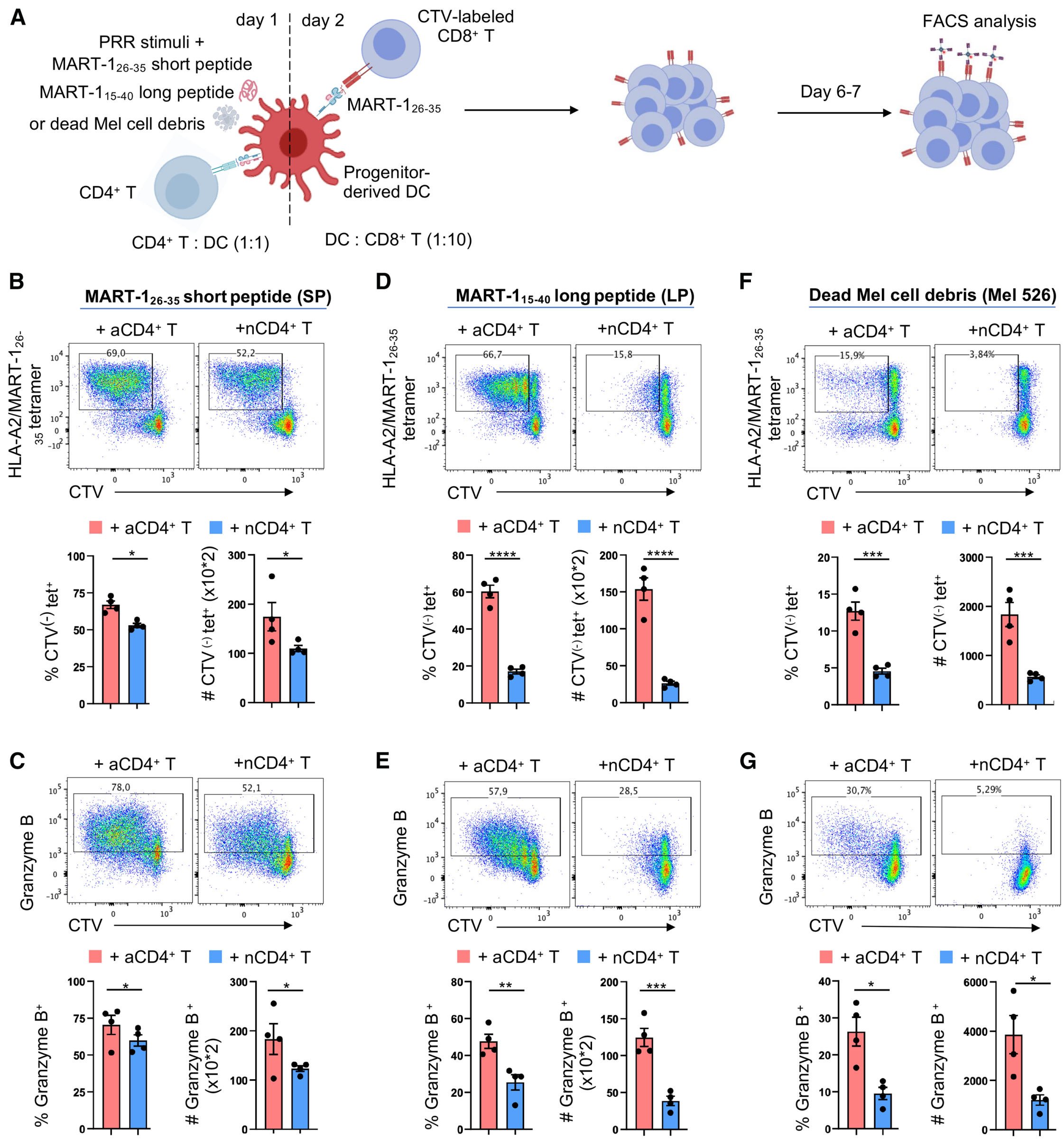


**Figure 4. Progenitor-derived DCs have the ability to relay CD4$^+$ T cell help for CTL responses**

(A) Scheme of the tumor antigen-specific CTL priming system. On day 1, ~5,000 total progenitor-derived DCs from HLA-A2$^+$ healthy donors were incubated with equal numbers of activated (a) or naive (n) CD4$^+$ T cells, MART-1 peptide or MART-1$^+$ melanoma cell debris, and a mixture of PRR stimuli. On day 2, ~50,000 CD8$^+$ T cells transduced to express the MART-1$_{26–35}$/HLA-A2-specific TCR and having a stem cell-like memory T cell (T$_{SCM}$) phenotype[19] were added. The T cell response was readout at day 6 or 7 after co-culture.

(B, D, and F) CD8$^+$ T cell proliferation to (B) MART-1$_{26–35}$ short peptide (SP), (D) MART-1$_{15–40}$ long peptide (LP), or (F) dead Mel526 cell debris based on CTV dilution. Upper: primary flow cytometric data. Lower: frequency (%) of MART-1$_{26–35}$/HLA-A2-specific (tet$^+$) cells within CTV$^{(-)}$ CD8$^+$ T cells, and their absolute, live cell number (#).

(C, E, and G) CTL response to (C) MART-1$_{26–35}$ SP, (E) MART-1$_{15–40}$ LP, or (G) dead Mel526 cell debris based on intracellular granzyme B staining. Upper: primary flow cytometric data. Lower: frequency of granzyme B$^+$ cells among CD8$^+$ T cells, and their absolute, live cell number.

Data are pooled from four ($n$ = 4) independent experiments and are shown as means ± SEM. *$p < 0.05$, **$p < 0.01$, ***$p < 0.001$, ****$p < 0.0001$ (two-sided unpaired Student's $t$ test). See also Figure S5.

activation markers (Figure 5A; Figure S7A). Tetramers conjugated to two fluorochromes improved detection specificity (Figures 5B and 5F). In the help setting with activated CD4$^+$ T cells, MART-1$_{26–53}$-specific CD8$^+$ T cells were significantly amplified to 0.42% ± 0.04% of total CD8$^+$ T cells, versus 0.14% ± 0.03% in the "no help" setting with naive CD4$^+$ T cells, which was similar to the frequency in the no antigen control (Figures 5B and 5C). Likewise, the frequency of HLA-A2/CDK4$_{23–32}$ tet$^+$CD8$^+$ T cells

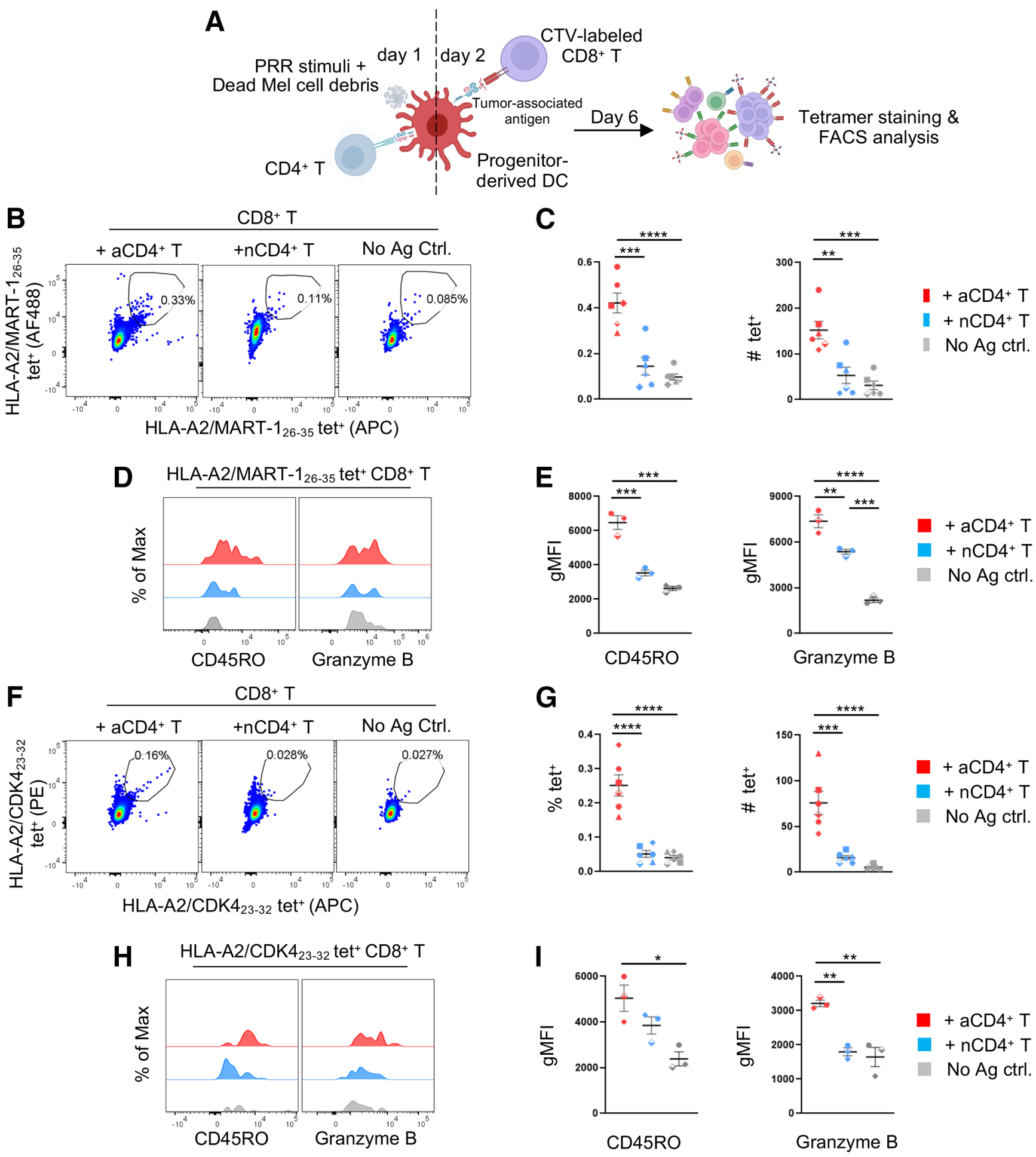


**Figure 5. Detecting tumor antigen-specific CD8$^+$ T cells in healthy donor blood with "helped" progenitor-derived DC**

Per assay, ∼50,000 CD8$^+$ T cells sorted from PBMCs were co-cultured with ∼5,000 progenitor-derived DCs loaded with dead Mel526 cell debris in the presence of ∼5,000 activated (a)- or naive (n)CD4$^+$ T cells.

Tumor antigen-reactive CD8$^+$ T cells were detected using peptide/MHC-I tetramers and T cell activation markers.

(A) Experimental design.

(B) Flow cytometric plots depicting HLA-A2/MART-$1_{26–35}$ tetramer (tet) staining of CD8$^+$ T cells after culture.

(C) Frequency (%) of and total number (#) of MART-$1_{26–35}$ tet$^+$ CD8$^+$ T cells within total CD8$^+$ T cells.

(D and E) Histograms (D) and gMFI quantifications (E) of CD45RO and granzyme B expression by MART-$1_{26–35}$ tet$^+$ CD8$^+$ T cells under indicated conditions.

(F) Flow cytometric plots depicting HLA-A2/CDK$4_{23–32}$ tet staining of CD8$^+$ T cells after culture.

(G) Frequency and absolute number (#) of CDK$4_{23–32}$ tet$^+$ cells within total CD8$^+$ T cells.

(H and I) Histogram (H) and gMFI quantifications (I) of CD45RO and granzyme B expression by CDK$4_{23–32}$ tet$^+$ CD8$^+$ T cells under indicated conditions.

Data are pooled from six ($n$ = 6 in C and G) and three ($n$ = 3 in E and I) independent donors and are shown as means ± SEM. *$p$ < 0.05, **$p$ < 0.01, ***$p$ < 0.001, ****$p$ < 0.0001. Statistical analyses were performed using one-way ANOVA with Tukey's multiple comparisons for (C), (E), (G), and (I). See also Figures S7A–S7D.

was increased to 0.25% ± 0.03% in the help setting, versus 0.05% ± 0.01% in the no help setting (Figures 5F and 5G). Absolute numbers of tetramer$^+$CD8$^+$ T cells were also increased with CD4$^+$ T cell help (Figures 5C and G). Moreover, MART-$1_{26–53}$- and CDK$4_{23–32}$-specific CD8$^+$ T cells in the help setting showed enhanced effector differentiation with increased expression levels of CD45RO and granzyme B (Figures 5D, 5E, 5H, and 5I). In the help setting, CD8$^+$ T cells specific for HLA-A2/MAGE$3_{271–279}$ were likewise amplified and thereby more easily detected (Figures S7B–S7D). We conclude that our platform of *in vitro* help delivery to CD8$^+$ T cells with progenitor-derived DCs amplifies tumor-specific CD8$^+$ T cells and thereby facilitates their detection among T cells present in the blood of adult healthy donors.

We next compared side-by-side the ability of progenitor-derived DCs and two commonly used antigen-presenting cell (APC) types, namely moDCs[58] and B cells,[59] to detect tumor antigen-specific CD8$^+$ T cells. For this purpose, we used matched APCs from the same donors ($n$ = 3) in the same experiment, reading out enrichment of detected MART-$1_{26–35}$-specific CD8$^+$ T cells after stimulation with the different APCs that had been stimulated with activated or naive CD4$^+$ T cells (Figure 6A; Figure S7E). Progenitor-derived DCs stimulated by activated CD4$^+$ T cells induced a higher frequency and absolute number of MART-$1_{26–35}$-specific CD8$^+$ T cells compared with moDCs, B cells, or conditions without APCs (Figures 6B and 6C). In addition, the CD8$^+$ T cells primed by progenitor-derived DCs differentiated more effectively into CTLs, as indicated by granzyme B expression, especially under CD4$^+$ T cell help conditions (Figures 6D–6F; Figure S7F).

## Helped progenitor-derived DCs enhance priming of functional tumor-specific CTLs

We next assessed whether we could use the same method to detect melanoma-specific CD8$^+$ T cells in healthy donor blood without the use of MHC tetramers, i.e., T cells with undefined tumor specificity (Figure 7A). Based on CTV dilution, a much higher proportion of CD8$^+$ T cells proliferated in response to Mel562 debris presented by helped as compared to non-helped DCs (Figures 7B and 7C; Figure S8A). The proliferating cells also displayed effector differentiation, defined by granzyme B and IFN-γ expression (Figures 7D–7G; Figure S8B). To assess killing capacity, DC-primed CD8$^+$ cells were sorted and co-cultured with Mel526 cells for 24 h in the IncuCyte,[22] monitoring live/dead cells in real time through captured images and measuring target cell killing based on caspase-3/7 activity (Figure 7H;

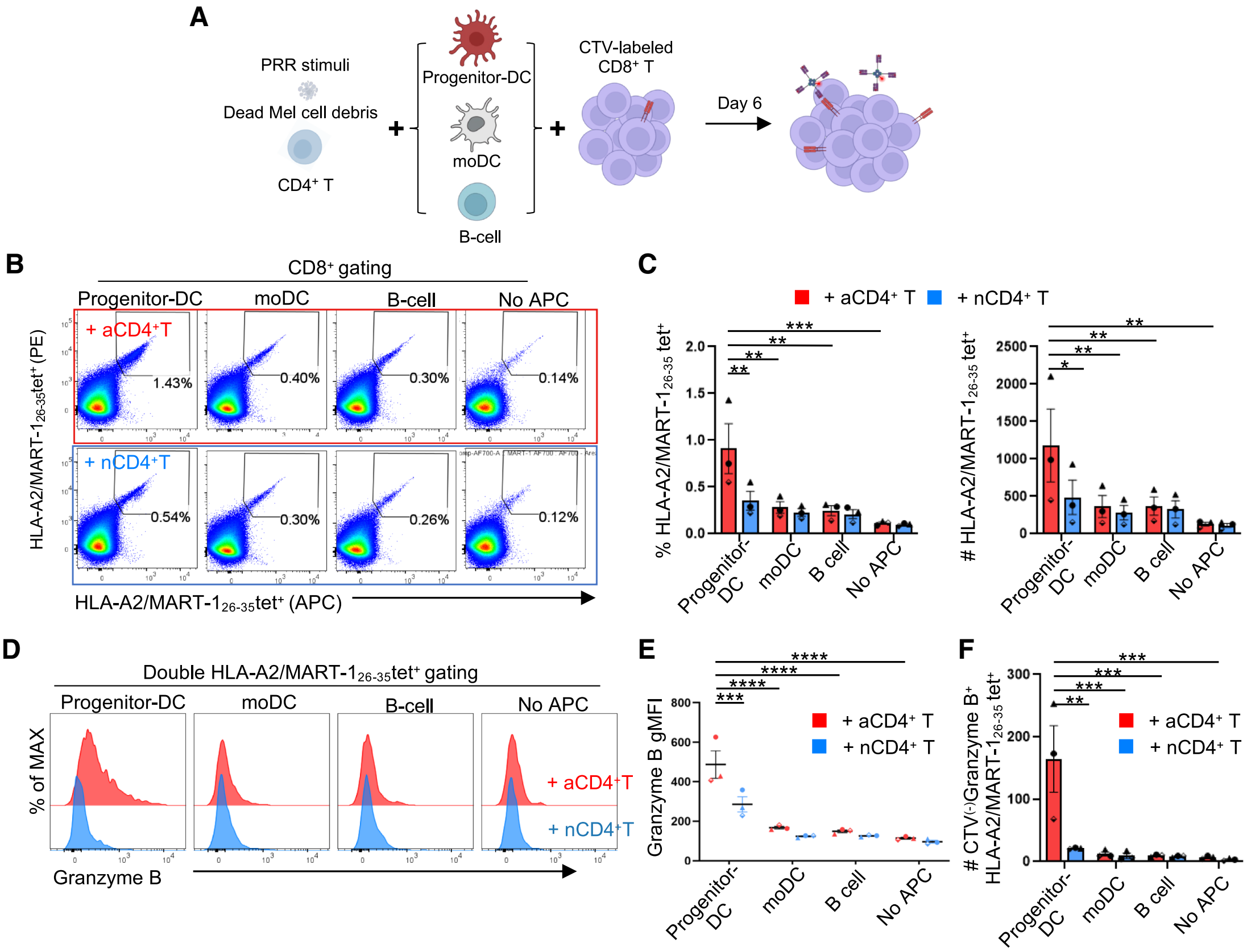


**Figure 6. Helped progenitor-derived DCs efficiently detect tumor antigen-specific CD8$^+$ T cells in blood and outperform moDCs and B cells**

Progenitor-derived DCs, moDCs, and B cells from matched donors were compared for their ability to detect and induce activation of endogenous MART$_{26–35}$-specific CD8$^+$ T cells from blood.

(A) Schematic of the experimental design. On day 1, ~100,000 total progenitor-derived DCs, moDCs, or B cells from HLA-A2$^+$ healthy donors were incubated with equal numbers of activated (a) or naive (n) CD4$^+$ T cells, Mel526 cell debris, and a mixture of PRR stimuli. On day 2, ~1,000,000 polyclonal CD8$^+$ T cells were added. The T cell response was readout at day 6 after co-culture.

(B) Representative flow cytometric plots showing double staining with MART-1$_{26–35}$/HLA-A2 tetramers for indicated conditions.

(C) Frequency (%) of MART-1$_{26–35}$/HLA-A2 tetramer-positive cells among CD8$^+$ T cells, and their absolute number (#).

(D) Representative histogram showing granzyme B expression CD8$^+$ T cells under the indicated conditions.

(E) Quantification of gMFI for granzyme B under the indicated conditions.

(F) Absolute number (#) of CTV$^{(-)}$granzyme B$^+$ MART-1$_{26–35}$/HLA-A2 tetramer$^+$ CD8$^+$ T cells under the indicated conditions.

Data are pooled from three ($n = 3$) healthy donors in (D), (F), and (G) and are shown as means ± SEM. *$p < 0.05$, **$p < 0.01$, ***$p < 0.001$, ****$p < 0.0001$ by one-way ANOVA with Tukey's multiple comparisons. See also Figures S7E and S7F.

Figure S8C). At the end of the assay, suspension cells were analyzed by flow cytometry, and adherent Mel526 cells were fixed and stained (Figure 7H). Proliferating CTV$^{(-)}$CD8$^+$ T cells were considered tumor reactive (Figure 7I) and showed higher granzyme B production and cell surface expression of the granule exocytosis marker CD107a after stimulation by helped versus non-helped DCs (Figure 7J). Accordingly, killing of Mel526 cells was shown by a loss of live tumor cell counts (Figures 7K and 7L), effector caspase-3/-7 activity (Figure 7M), and reduced Mel526 confluence (Figure 7N). In contrast, non-proliferating CD8$^+$ T cells (CTV$^+$) showed limited cytotoxicity regardless of help (Figure S9). Together, these results demonstrate that helped progenitor-derived DCs efficiently cross-prime tumor-specific CTLs from polyclonal CD8$^+$ T cells in adult human blood, enabling effective tumor cell killing.

## DISCUSSION

We have established a robust protocol to generate progeny that is enriched for cDC1-like cells from CD34$^+$c-KIT$^+$ progenitors, as present in non-mobilized peripheral blood of healthy adult donors. The progenitor-derived cDC1-like cell population generated consists of up to 60% of cDC1-like cells that resemble *ex vivo* cDC1s in their response to activated CD4$^+$ T cells, in terms of upregulation of diverse molecules that are part of the cDC1 licensing program and involved in antigen (cross-)presentation and T cell priming. Importantly, when licensed by CD4$^+$ T cells, progenitor-derived DCs potently cross-primed tumor-reactive CD8$^+$ T cells from a polyclonal T cell repertoire as present in the blood of healthy donors and promote CTL effector differentiation. Our DC generation protocol and associated

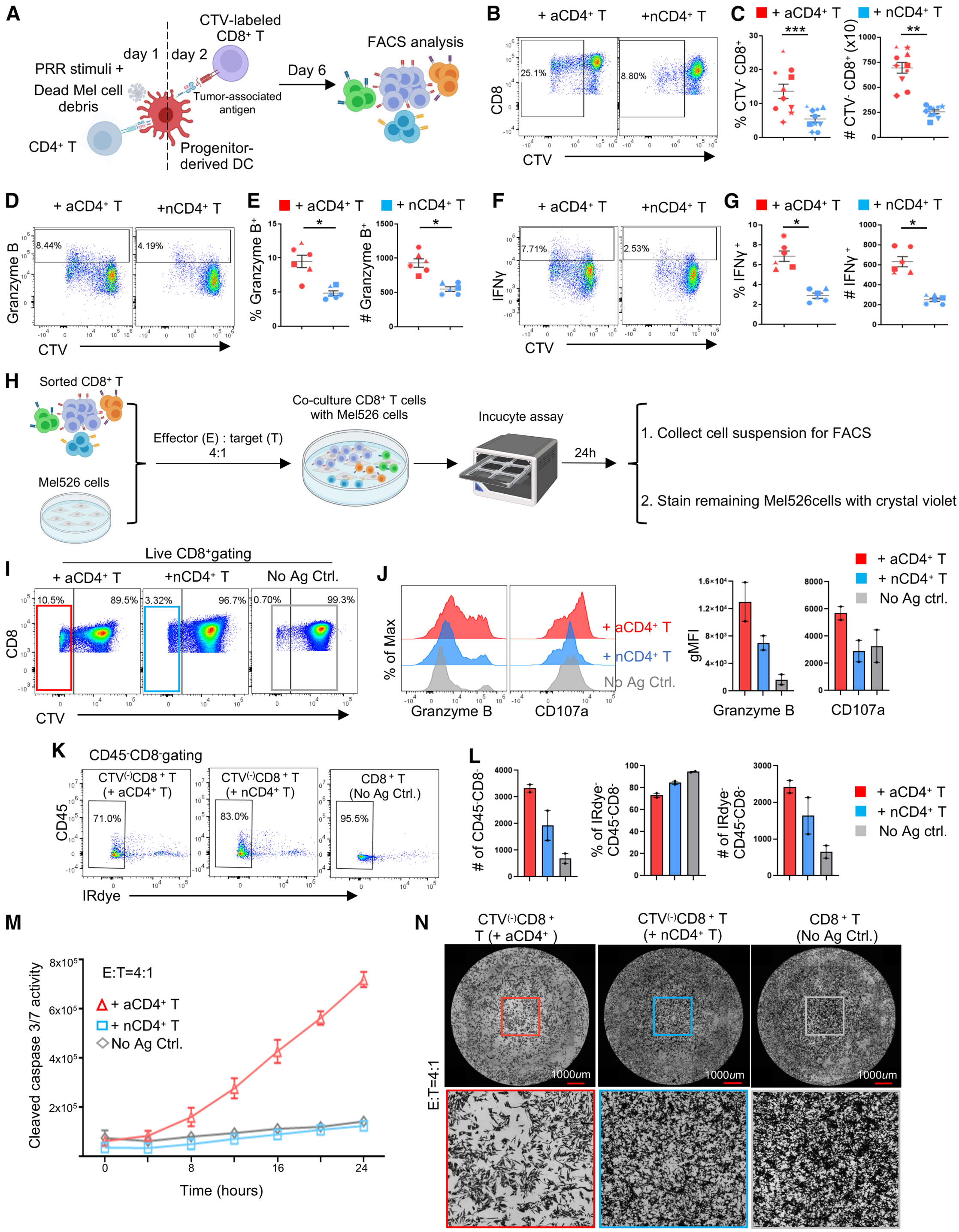


**Figure 7. "Helped" progenitor-derived DCs enhance priming of functional tumor-specific CTLs**

(A–G) ~50,000 $CD8^+$ T cells were sorted from PBMCs and co-cultured with ~5,000 progenitor-derived DCs loaded with dead Mel526 cell debris in the presence of ~5,000 activated (a) or naive (n) $CD4^+$ T cells for 6 days, after which the $CD8^+$ T cell response was measured by flow cytometry.

(A) Experimental design.

(B) Flow cytometric plots depicting $CD8^+$ T cell proliferation based on CTV dilution.

(C) Frequency (%) and absolute number (#) of live $CTV^{(-)}$ cells within total $CD8^+$ T cells.

(D and F) Flow cytometric plots depicting the CTL response based on intracellular granzyme B (D) and IFN-γ (F) staining.

(E and G) Frequency (%) and absolute, live cell number (#) of granzyme $B^+$ cells (E) and IFN-$\gamma^+$ cells (G) within total $CD8^+$ T cells.

(legend continued on next page)

CTL-priming system therefore constitute a versatile platform to (1) enhance detection of tumor-specific CTLs in both circulation and tumor tissue, (2) qualitatively improve $CD8^+$ T cell products for adoptive immunotherapy, and (3) broaden clinical access to cDC1-based cell vaccines.

cDC1s can home from peripheral tissues to LNs, cross-present particulate antigens to $CD8^+$ T cells,[12,13] and relay $CD4^+$ T cell help for the CTL response.[19] These combined properties make cDC1s the superior cell type to induce tumor-specific CTL response as compared to cDC2s, moDCs, and pDCs.[19,60] While cDC1s are ideal candidates for cellular vaccines, their scarcity in peripheral blood severely limits their therapeutic use. In the field, efforts to generate human cDC1s from progenitors *in vitro* are ongoing and have already led to production of cells closely resembling *ex vivo* cDC1s, using mobilized adult PBMCs or cord blood as a progenitor cell source. We aimed to improve the protocol to enable use of non-mobilized adult blood, ideally not only from healthy donors but also from patients, to develop autologous systems. In a side-by-side comparison, our culture system, which adds MSCs to OP9-hDLL1 as feeder cells and uses an adapted cytokine cocktail, enabled the generation of cDC1-like cells from relatively low numbers of HPCs (~1,000 cells) from non-mobilized adult blood, whereas a protocol described by Balan et al.[49] did not. During finalization of our study, Balan et al. reported cDC1-like cell generation from non-mobilized blood using a serum-free, OP9 feeder cell-based culture system.[48,61] Thus, we envision that an MSC-hDLL1 feeder system under serum-free conditions[61] may provide a suitable platform for generating human cDC1s at sufficient yield in the future. Such integration could pave the way for defined large-scale production of cDC1s for clinical use. Future iterations may lead to replacement of feeder cells with defined cytokine cocktails. Developing feeder-free or fully defined culture conditions will be important to meet good manufacturing practice (GMP) standards and facilitate broader clinical applicability.

Our culture system also gives rise to $SIRP\alpha^+$ cDC2-like cells, while this study focused on bulk cDC1-like cells. As canonical markers for *ex vivo* DCs are less reliable for *in-vitro*-generated DCs, single-cell mRNA sequencing may help identify distinguishing new surface markers for *in vitro* cultures. Mouse models suggest that cDC2s are critical for priming $CD4^+$ T cells,[21] and DC subset crosstalk can enhance immune responses.[62] Thus, incorporating both cDC1- and cDC2-like cells may offer synergistic benefits and better mimic physiological DC/T cell interactions in vaccine strategies.

Upon $CD4^+$ T cell help, the progenitor-derived DC population enriched for cDC1-like cells expressed a transcriptional program that we previously defined in licensed/helped bona fide *ex vivo* human cDC1s.[19] This program, as induced by $CD4^+$ T cell help via CD40L and IFN-I, enhances cDC1 antigen cross-presentation, cross-priming, T cell recruitment, and costimulation.[21,22,63] The cDC1 licensing program is conserved in mouse and humans.[19–22,24,25] As a result, the cDC1 effectively induces $CD8^+$ T cell expansion and CTL effector differentiation.[19,22,24] We show here how progenitor-derived DCs enriched for cDC1-like cells can be used to identify and functionally program tumor antigen-specific $CD8^+$ T cells. Using tumor debris as an antigen source and healthy donor blood as the T cell input, we detected rare tumor-specific $CD8^+$ T cells, both by peptide/MHC tetramer staining and functional readouts (granzyme B and IFN-$\gamma$). Importantly, inclusion of $CD4^+$ T cell help significantly increased detection rates, underscoring the impact of DC licensing on optimal CTL generation. Helped/licensed progenitor-derived DCs enriched for cDC1-like cells thus provide a sensitive tool for identifying tumor-specific $CD8^+$ T cells in peripheral blood and tumor tissue, a long-standing challenge given their low precursor frequencies.[42–44]

Current strategies to detect and expand rare tumor-specific T cells predominantly rely on moDCs and B cells as APCs,[58,59] largely due to their accessibility. However, as compared to cDC1s, moDCs[19] and likely also B cells are less specialized in cross-presentation of cell-associated antigens and do not respond to $CD4^+$ T cell help to upregulate cross-presentation and cross-priming abilities. Accordingly, in a side-by-side comparison with moDCs and B cells as APCs, our helped/licensed progenitor-derived DC platform showed higher sensitivity in detecting rare tumor antigen-specific $CD8^+$ T cells, particularly in the presence of $CD4^+$ T cell help.

Our methodology may benefit ACT by helping to identify circulating tumor-reactive $CD8^+$ T cells that are increasingly leveraged for TCR-engineered therapies.[42–44] Peripheral blood is currently being explored as an alternative to TILs.[64,65] Notably, neoantigen-specific T cells from peripheral blood share phenotypic markers with TILs but are generally less terminally differentiated,[42] potentially rendering them more expandable and therapeutically effective. Our platform could therefore facilitate the enrichment and isolation of such cells, or their TCRs, improving the quality of the input material for TCR- or TIL-based ACT. Moreover, $CD8^+$ T cells primed by helped

(H–N) Killing assay was performed using live proliferative (CTV negative) $CD8^+$ T cells purified from the CTL priming system and Mel526 tumor cells at an effector/target (E/T) ratio of 4:1 and analyzed by IncuCyte during 24 h. At the end of the assay, the cell suspension was pooled and analyzed by flow cytometry. Remaining Mel526 cells in each well were fixed and stained with crystal violet.

(H) Experimental design.

(I) Gating strategy for isolating $CTV^{(-)}CD8^+$ T cells from the CTL priming system. Total live $CD8^+$ T cells from no antigen setting were used as reference.

(J) Histograms and gMFI quantifications for granzyme B and cell surface CD107$\alpha$ expression by $CD8^+$ T cells after the killing assay.

(K) Flow cytometric plots depicting total and detached Mel526 cells in the supernatant following the killing assay, as indicated by $CD45^{(-)}CD8^{(-)}$ and $IRdye^{(-)}CD45^{(-)}CD8^{(-)}$ populations under indicated conditions.

(L) Total number (#) of tumor cells, as well as frequency and number (#) of tumor cell loss measured in the supernatant.

(M) Caspase-3/7 activity in tumor cells during the killing assay under the indicated conditions.

(N) Representative CCD microscopic images depicting remaining Mel526 cells in the plate at 24 h post co-culture under indicated conditions.

Data are pooled from ten ($n = 10$ in C) and six ($n = 6$ in E and G) independent donors and are shown as means ± SEM. *$p < 0.05$, **$p < 0.01$, ***$p < 0.001$ (two-sided paired Student's *t* test in C, E, and G). Data are pooled from two ($n = 2$ in J, L, and N) independent donors and are shown as means ± SD. See also Figures S8 and S9.

progenitor-derived DCs exhibited superior cytolytic activity. These functional gains are clinically meaningful, as ACT will benefit from the availability of optimally functional CTLs,[66] and high-quality CTLs are more likely to persist and retain effector function within the TME, where chronic antigen exposure, cytokine-mediated suppression, and exhaustion pose significant barriers.[67–71]

In conclusion, generating human cDC1-like cells from progenitors and using them to deliver $CD4^+$ T cell help enables sensitive detection of circulating tumor-reactive $CD8^+$ T cells for TCR discovery or ACT and offers a strategy to enhance their functional quality. Future validation in cancer patients and incorporation of tumor-specific peptide/MHC multimers[72] will further improve the application of this platform for personalized immunotherapy.

### Limitations of the study

The culture platform presented generates a DC population that is enriched for cDC1-like cells but is still heterogeneous and includes cDC2-like and monocytic cells. In addition, the cDC1-like cell culture relies on feeder cells, which may limit use of the method to generate GMP-compatible cell products. Furthermore, the broader clinical utility of this platform should be validated by the detection of diverse tumor (neo)antigen-specific CTL responses in blood and tumor material of cancer patients.

## RESOURCE AVAILABILITY

### Lead contact

Requests for further information and resources should be directed to and will be fulfilled by the lead contact, Yanling Xiao (y.xiao@lumc.nl).

### Materials availability

This study did not generate new materials.

### Data and code availability

- All primary data are available from the lead contact upon request.
- This study does not report original code.
- Any additional information required to reanalyze the data reported in this paper is available from the lead contact upon request.

## ACKNOWLEDGMENTS

This project was funded by an Antoni van Leeuwenhoek Foundation grant from the Bakker family to J.B. and Y.X. It was additionally funded by a grant from Oncode to J.B., Dutch Cancer Society (KWF) grant 11079 to J.B. and Y.X., the China Scholarship Consil (CSC)-Leiden University joint scholarship programme to Y.W.. We thank all personnel of the flow cytometry facilities at the Netherlands Cancer Institute and Leiden University Medical Center for their excellent technical support. We thank E. de Vries and L. Brocks for assistance. We thank S.J. van der Burg and J.W. Drijfhout for advice. Images and clipart used in the figures are derived from BioRender.com.

## AUTHOR CONTRIBUTIONS

Y.W. and X.L. designed and performed the experiments, analyzed the data, and wrote the manuscript. T.d.W., E.S., and K.v.L. assisted with cell sorting and co-culture experiments. J.v.d.B. and M.d.B. contributed to sample collection and performed the experiments. M.J.P.W., S.d.K., and N.F.C.C.d.M. provided scientific insight, with M.J.P.W. also assisting with Nanostring experiments. T.N.S. provided scientific insight and the MART-1-specific TCR construct. C.S. contributed to the experiments and provided scientific insight. J.B. interpreted the data and revised the manuscript. Y.X. designed and supervised the experiments, analyzed/interpreted the data, and wrote/revised the manuscript. Y.X. and J.B. designed the study and procured its funding. All authors reviewed the manuscript.

## DECLARATION OF INTERESTS

The authors declare that the research was conducted in the absence of any commercial or financial relationships that could be construed as a potential conflict of interest.

## STAR★METHODS

Detailed methods are provided in the online version of this paper and include the following:

- KEY RESOURCES TABLE
- EXPERIMENTAL MODEL AND STUDY DETAILS
  - Human peripheral blood samples
  - Feeder cells
- METHOD DETAILS
  - *In vitro* generation of blood progenitor-derived DC
  - Flow cytometry staining and cell sorting
  - NanoString nCounter gene expression analysis
  - Tumor antigen-specific CTL priming platform
  - Antigen presenting cell comparison for tumor specific CTL detection and expansion
  - Allogeneic mixed lymphocyte reaction
  - IncuCyte T-cell killing assay
- QUANTIFICATION AND STATISTICAL ANALYSIS

## SUPPLEMENTAL INFORMATION

Supplementary data related to this article can be found online at https://doi.org/10.1016/j.crmeth.2026.101533.

Received: November 13, 2025
Revised: May 4, 2026
Accepted: June 25, 2026
Published: July 22, 2026

## REFERENCES

1. Reis e Sousa, C. (2006). Dendritic cells in a mature age. Nat. Rev. Immunol. *6*, 476–483. https://doi.org/10.1038/nri1845.
2. Pulendran, B. (2015). The Varieties of Immunological Experience: Of Pathogens, Stress, and Dendritic Cells. Annu. Rev. Immunol. *33*, 563–606. https://doi.org/10.1146/annurev-immunol-020711-075049.
3. Merad, M., Sathe, P., Helft, J., Miller, J., and Mortha, A. (2013). The Dendritic Cell Lineage: Ontogeny and Function of Dendritic Cells and Their Subsets in the Steady State and the Inflamed Setting. Annu. Rev. Immunol. *31*, 563–604. https://doi.org/10.1146/annurev-immunol-020711-074950.
4. Segura, E. (2022). Human dendritic cell subsets: An updated view of their ontogeny and functional specialization. Eur. J. Immunol. *52*, 1759–1767. https://doi.org/10.1002/eji.202149632.
5. Collin, M., and Bigley, V. (2018). Human dendritic cell subsets: an update. Immunology *154*, 3–20. https://doi.org/10.1111/imm.12888.
6. Dutertre, C.-A., Becht, E., Irac, S.E., Khalilnezhad, A., Narang, V., Khalilnezhad, S., Ng, P.Y., van den Hoogen, L.L., Leong, J.Y., Lee, B., et al. (2019). Single-Cell Analysis of Human Mononuclear Phagocytes Reveals Subset-Defining Markers and Identifies Circulating Inflammatory Dendritic Cells. Immunity *51*, 573–589.e8. https://doi.org/10.1016/j.immuni.2019.08.008.
7. Villani, A.-C., Satija, R., Reynolds, G., Sarkizova, S., Shekhar, K., Fletcher, J., Griesbeck, M., Butler, A., Zheng, S., Lazo, S., et al. (2017). Single-cell RNA-seq reveals new types of human blood dendritic cells, monocytes

and progenitors. Science *356*, eaah4573. https://doi.org/10.1126/science.aah4573.

8. Karsunky, H., Merad, M., Cozzio, A., Weissman, I.L., and Manz, M.G. (2003). Flt3 Ligand Regulates Dendritic Cell Development from Flt3+ Lymphoid and Myeloid-committed Progenitors to Flt3+ Dendritic Cells In Vivo. J. Exp. Med. *198*, 305–313. https://doi.org/10.1084/jem.20030323.
9. Murphy, T.L., Grajales-Reyes, G.E., Wu, X., Tussiwand, R., Briseño, C.G., Iwata, A., Kretzer, N.M., Durai, V., and Murphy, K.M. (2016). Transcriptional Control of Dendritic Cell Development. Annu. Rev. Immunol. *34*, 93–119. https://doi.org/10.1146/annurev-immunol-032713-120204.
10. Cytlak, U., Resteu, A., Pagan, S., Green, K., Milne, P., Maisuria, S., McDonald, D., Hulme, G., Filby, A., Carpenter, B., et al. (2020). Differential IRF8 Transcription Factor Requirement Defines Two Pathways of Dendritic Cell Development in Humans. Immunity *53*, 353–370.e8. https://doi.org/10.1016/j.immuni.2020.07.003.
11. Durand, M., and Segura, E. (2015). The known unknowns of the human dendritic cell network. Front. Immunol. *6*, 129. https://doi.org/10.3389/fimmu.2015.00129.
12. Broz, M.L., and Krummel, M.F. (2015). The Emerging Understanding of Myeloid Cells as Partners and Targets in Tumor Rejection. Cancer Immunol. Res. *3*, 313–319. https://doi.org/10.1158/2326-6066.CIR-15-0041.
13. Roberts, E.W., Broz, M.L., Binnewies, M., Headley, M.B., Nelson, A.E., Wolf, D.M., Kaisho, T., Bogunovic, D., Bhardwaj, N., and Krummel, M.F. (2016). Critical Role for CD103 +/CD141 + Dendritic Cells Bearing CCR7 for Tumor Antigen Trafficking and Priming of T Cell Immunity in Melanoma. Cancer Cell *30*, 324–336. https://doi.org/10.1016/j.ccell.2016.06.003.
14. den Haan, J.M., Lehar, S.M., and Bevan, M.J. (2000). Cd8+ but Not Cd8− Dendritic Cells Cross-Prime Cytotoxic T Cells in Vivo. J. Exp. Med. *192*, 1685–1696. https://doi.org/10.1084/jem.192.12.1685.
15. Schenkel, J.M., Herbst, R.H., Canner, D., Li, A., Hillman, M., Shanahan, S.-L., Gibbons, G., Smith, O.C., Kim, J.Y., Westcott, P., et al. (2021). Conventional type I dendritic cells maintain a reservoir of proliferative tumor-antigen specific TCF-1+ CD8+ T cells in tumor-draining lymph nodes. Immunity *54*, 2338–2353.e6. https://doi.org/10.1016/j.immuni.2021.08.026.
16. Burger, M.L., Cruz, A.M., Crossland, G.E., Gaglia, G., Ritch, C.C., Blatt, S.E., Bhutkar, A., Canner, D., Kienka, T., Tavana, S.Z., et al. (2021). Antigen dominance hierarchies shape TCF1+ progenitor CD8 T cell phenotypes in tumors. Cell *184*, 4996–5014.e26. https://doi.org/10.1016/j.cell.2021.08.020.
17. Duraiswamy, J., Turrini, R., Minasyan, A., Barras, D., Crespo, I., Grimm, A.J., Casado, J., Genolet, R., Benedetti, F., Wicky, A., et al. (2021). Myeloid antigen-presenting cell niches sustain antitumor T cells and license PD-1 blockade via CD28 costimulation. Cancer Cell *39*, 1623–1642.e20. https://doi.org/10.1016/j.ccell.2021.10.008.
18. Prokhnevska, N., Cardenas, M.A., Valanparambil, R.M., Sobierajska, E., Barwick, B.G., Jansen, C., Reyes Moon, A., Gregorova, P., delBalzo, L., Greenwald, R., et al. (2023). CD8+ T cell activation in cancer comprises an initial activation phase in lymph nodes followed by effector differentiation within the tumor. Immunity *56*, 107–124.e5. https://doi.org/10.1016/j.immuni.2022.12.002.
19. Lei, X., Khatri, I., de Wit, T., de Rink, I., Nieuwland, M., Kerkhoven, R., van Eenennaam, H., Sun, C., Garg, A.D., Borst, J., and Xiao, Y. (2023). CD4+ helper T cells endow cDC1 with cancer-impeding functions in the human tumor micro-environment. Nat. Commun. *14*, 217. https://doi.org/10.1038/s41467-022-35615-5.
20. Wu, R., Ohara, R.A., Jo, S., Liu, T.-T., Ferris, S.T., Ou, F., Kim, S., Theisen, D.J., Anderson, D.A., Wong, B.W., et al. (2022). Mechanisms of CD40-dependent cDC1 licensing beyond costimulation. Nat. Immunol. *23*, 1536–1550. https://doi.org/10.1038/s41590-022-01324-w.
21. Borst, J., Ahrends, T., Bąbała, N., Melief, C.J.M., and Kastenmüller, W. (2018). CD4+ T cell help in cancer immunology and immunotherapy. Nat. Rev. Immunol. *18*, 635–647. https://doi.org/10.1038/s41577-018-0044-0.
22. Lei, X., de Groot, D.C., Welters, M.J.P., de Wit, T., Schrama, E., van Eenennaam, H., Santegoets, S.J., Oosenbrug, T., van der Veen, A., Vos, J.L., et al. (2024). CD4+ T cells produce IFN-I to license cDC1s for induction of cytotoxic T-cell activity in human tumors. Cell. Mol. Immunol. *21*, 374–392. https://doi.org/10.1038/s41423-024-01133-1.
23. Ferris, S.T., Durai, V., Wu, R., Theisen, D.J., Ward, J.P., Bern, M.D., Davidson, J.T., Bagadia, P., Liu, T., Briseño, C.G., et al. (2020). cDC1 prime and are licensed by CD4+ T cells to induce anti-tumour immunity. Nature *584*, 624–629. https://doi.org/10.1038/s41586-020-2611-3.
24. Jobin, K., Seetharama, D., Rüttger, L., Fenton, C., Kharybina, E., Wirsching, A., Huang, A., Knöpper, K., Kaisho, T., Busch, D.H., et al. (2025). A distinct priming phase regulates CD8 T cell immunity by orchestrating paracrine IL-2 signals. Science *388*, eadq1405. https://doi.org/10.1126/science.adq1405.
25. Ahrends, T., Spanjaard, A., Pilzecker, B., Bąbała, N., Bovens, A., Xiao, Y., Jacobs, H., and Borst, J. (2017). CD4+T Cell Help Confers a Cytotoxic T Cell Effector Program Including Coinhibitory Receptor Downregulation and Increased Tissue Invasiveness. Immunity *47*, 848–861.e5. https://doi.org/10.1016/j.immuni.2017.10.009.
26. Ahrends, T., Busselaar, J., Severson, T.M., Bąbała, N., de Vries, E., Bovens, A., Wessels, L., van Leeuwen, F., and Borst, J. (2019). CD4+ T cell help creates memory CD8+ T cells with innate and help-independent recall capacities. Nat. Commun. *10*, 5531. https://doi.org/10.1038/s41467-019-13438-1.
27. Spranger, S., Dai, D., Horton, B., and Gajewski, T.F. (2017). Tumor-Residing Batf3 Dendritic Cells Are Required for Effector T Cell Trafficking and Adoptive T Cell Therapy. Cancer Cell *31*, 711–723.e4. https://doi.org/10.1016/j.ccell.2017.04.003.
28. Meiser, P., Knolle, M.A., Hirschberger, A., de Almeida, G.P., Bayerl, F., Lacher, S., Pedde, A.-M., Flommersfeld, S., Hönninger, J., Stark, L., et al. (2023). A distinct stimulatory cDC1 subpopulation amplifies CD8+ T cell responses in tumors for protective anti-cancer immunity. Cancer Cell *41*, 1498–1515.e10. https://doi.org/10.1016/j.ccell.2023.06.008.
29. Oh, S.A., Wu, D.-C., Cheung, J., Navarro, A., Xiong, H., Cubas, R., Totpal, K., Chiu, H., Wu, Y., Comps-Agrar, L., et al. (2020). PD-L1 expression by dendritic cells is a key regulator of T-cell immunity in cancer. Nat. Cancer *1*, 681–691. https://doi.org/10.1038/s43018-020-0075-x.
30. Larkin, J., Chiarion-Sileni, V., Gonzalez, R., Grob, J.-J., Rutkowski, P., Lao, C.D., Cowey, C.L., Schadendorf, D., Wagstaff, J., Dummer, R., et al. (2019). Five-Year Survival with Combined Nivolumab and Ipilimumab in Advanced Melanoma. N. Engl. J. Med. *381*, 1535–1546. https://doi.org/10.1056/NEJMoa1910836.
31. Garon, E.B., Rizvi, N.A., Hui, R., Leighl, N., Balmanoukian, A.S., Eder, J.P., Patnaik, A., Aggarwal, C., Gubens, M., Horn, L., et al. (2015). Pembrolizumab for the Treatment of Non–Small-Cell Lung Cancer. N. Engl. J. Med. *372*, 2018–2028. https://doi.org/10.1056/NEJMoa1501824.
32. Hamid, O., Robert, C., Daud, A., Hodi, F.S., Hwu, W.J., Kefford, R., Wolchok, J.D., Hersey, P., Joseph, R., Weber, J.S., et al. (2019). Five-year survival outcomes for patients with advanced melanoma treated with pembrolizumab in KEYNOTE-001. Ann. Oncol. *30*, 582–588. https://doi.org/10.1093/annonc/mdz011.
33. Luca, B.A., Steen, C.B., Matusiak, M., Azizi, A., Varma, S., Zhu, C., Przybyl, J., Espín-Pérez, A., Diehn, M., Alizadeh, A.A., et al. (2021). Atlas of clinically distinct cell states and ecosystems across human solid tumors. Cell *184*, 5482–5496.e28. https://doi.org/10.1016/j.cell.2021.09.014.
34. Rosenberg, S.A., and Restifo, N.P. (2015). Adoptive cell transfer as personalized immunotherapy for human cancer. Science *348*, 62–68. https://doi.org/10.1126/science.aaa4967.
35. Zhao, Q., Jiang, Y., Xiang, S., Kaboli, P.J., Shen, J., Zhao, Y., Wu, X., Du, F., Li, M., Cho, C.H., et al. (2021). Engineered TCR-T Cell Immunotherapy in Anticancer Precision Medicine: Pros and Cons. Front. Immunol. *12*, 658753. https://doi.org/10.3389/fimmu.2021.658753.

36. Cohen, C.J., Gartner, J.J., Horovitz-Fried, M., Shamalov, K., Trebska-McGowan, K., Bliskovsky, V.V., Parkhurst, M.R., Ankri, C., Prickett, T.D., Crystal, J.S., et al. (2015). Isolation of neoantigen-specific T cells from tumor and peripheral lymphocytes. J. Clin. Investig. *125*, 3981–3991. https://doi.org/10.1172/JCI82416.

37. Simoni, Y., Becht, E., Fehlings, M., Loh, C.Y., Koo, S.-L., Teng, K.W.W., Yeong, J.P.S., Nahar, R., Zhang, T., Kared, H., et al. (2018). Bystander CD8+ T cells are abundant and phenotypically distinct in human tumour infiltrates. Nature *557*, 575–579. https://doi.org/10.1038/s41586-018-0130-2.

38. Gros, A., Tran, E., Parkhurst, M.R., Ilyas, S., Pasetto, A., Groh, E.M., Robbins, P.F., Yossef, R., Garcia-Garijo, A., Fajardo, C.A., et al. (2019). Recognition of human gastrointestinal cancer neoantigens by circulating PD-1+ lymphocytes. J. Clin. Investig. *129*, 4992–5004. https://doi.org/10.1172/JCI127967.

39. van den Bulk, J., Verdegaal, E.M.E., Ruano, D., Ijsselsteijn, M.E., Visser, M., van der Breggen, R., Duhen, T., van der Ploeg, M., de Vries, N.L., Oosting, J., et al. (2019). Neoantigen-specific immunity in low mutation burden colorectal cancers of the consensus molecular subtype 4. Genome Med. *11*, 87. https://doi.org/10.1186/s13073-019-0697-8.

40. Moravec, Z., Zhao, Y., Voogd, R., Cook, D.R., Kinrot, S., Capra, B., Yang, H., Raud, B., Ou, J., Xuan, J., et al. (2025). Discovery of tumor-reactive T cell receptors by massively parallel library synthesis and screening. Nat. Biotechnol. *43*, 214–222. https://doi.org/10.1038/s41587-024-02210-6.

41. Kuilman, T., Schrikkema, D.S., Gadiot, J., Gomez-Eerland, R., Bies, L., Walker, J., Spaapen, R.M., Kok, H., Houg, D., Viyacheva, M., et al. (2025). Enabling next-generation engineered TCR-T therapies based on high-throughput TCR discovery from diagnostic tumor biopsies. Nat. Commun. *16*, 649. https://doi.org/10.1038/s41467-024-55420-6.

42. Yossef, R., Krishna, S., Sindiri, S., Lowery, F.J., Copeland, A.R., Gartner, J.J., Parkhurst, M.R., Parikh, N.B., Hitscherich, K.J., Levi, S.T., et al. (2023). Phenotypic signatures of circulating neoantigen-reactive CD8+ T cells in patients with metastatic cancers. Cancer Cell *41*, 2154–2165.e5. https://doi.org/10.1016/j.ccell.2023.11.005.

43. Malekzadeh, P., Yossef, R., Cafri, G., Paria, B.C., Lowery, F.J., Jafferji, M., Good, M.L., Sachs, A., Copeland, A.R., Kim, S.P., et al. (2020). Antigen Experienced T Cells from Peripheral Blood Recognize p53 Neoantigens. Clin. Cancer Res. *26*, 1267–1276. https://doi.org/10.1158/1078-0432.CCR-19-1874.

44. Cafri, G., Yossef, R., Pasetto, A., Deniger, D.C., Lu, Y.-C., Parkhurst, M., Gartner, J.J., Jia, L., Ray, S., Ngo, L.T., et al. (2019). Memory T cells targeting oncogenic mutations detected in peripheral blood of epithelial cancer patients. Nat. Commun. *10*, 449. https://doi.org/10.1038/s41467-019-08304-z.

45. Blank, C.U., Haanen, J.B., Ribas, A., and Schumacher, T.N. (2016). The "cancer immunogram.". Science *352*, 658–660. https://doi.org/10.1126/science.aaf2834.

46. Fesnak, A.D., June, C.H., and Levine, B.L. (2016). Engineered T cells: the promise and challenges of cancer immunotherapy. Nat. Rev. Cancer *16*, 566–581. https://doi.org/10.1038/nrc.2016.97.

47. O'Donnell, J.S., Teng, M.W.L., and Smyth, M.J. (2019). Cancer immunoediting and resistance to T cell-based immunotherapy. Nat. Rev. Clin. Oncol. *16*, 151–167. https://doi.org/10.1038/s41571-018-0142-8.

48. Kirkling, M.E., Cytlak, U., Lau, C.M., Lewis, K.L., Resteu, A., Khodadadi-Jamayran, A., Siebel, C.W., Salmon, H., Merad, M., Tsirigos, A., et al. (2018). Notch Signaling Facilitates In Vitro Generation of Cross-Presenting Classical Dendritic Cells. Cell Rep. *23*, 3658–3672.e6. https://doi.org/10.1016/j.celrep.2018.05.068.

49. Balan, S., Arnold-Schrauf, C., Abbas, A., Couespel, N., Savoret, J., Imperatore, F., Villani, A.C., Vu Manh, T.P., Bhardwaj, N., and Dalod, M. (2018). Large-Scale Human Dendritic Cell Differentiation Revealing Notch-Dependent Lineage Bifurcation and Heterogeneity. Cell Rep. *24*, 1902–1915.e6. https://doi.org/10.1016/j.celrep.2018.07.033.

50. van Eck van der Sluijs, J., van Ens, D., Thordardottir, S., Vodegel, D., Hermens, I., van der Waart, A.B., Falkenburg, J.H.F., Kester, M.G.D., de Rink, I., Heemskerk, M.H.M., et al. (2021). Clinically applicable CD34+-derived blood dendritic cell subsets exhibit key subset-specific features and potently boost anti-tumor T and NK cell responses. Cancer Immunol. Immunother. *70*, 3167–3181. https://doi.org/10.1007/s00262-021-02899-3.

51. Xiao, Y., Zijl, S., Wang, L., de Groot, D.C., van Tol, M.J., Lankester, A.C., and Borst, J. (2015). Identification of the Common Origins of Osteoclasts, Macrophages, and Dendritic Cells in Human Hematopoiesis. Stem Cell Rep. *4*, 984–994. https://doi.org/10.1016/j.stemcr.2015.04.012.

52. Xiao, Y., van Halteren, A.G.S., Lei, X., Borst, J., Steenwijk, E., de Wit, T., Grabowska, J., Voogd, R., Kemps, P., Picarsic, J., et al. (2020). Bone marrow–derived myeloid progenitors as driver mutation carriers in high- and low-risk Langerhans cell histiocytosis. Blood *136*, 2188–2199. https://doi.org/10.1182/blood.2020005209.

53. Xiao, Y., Peperzak, V., van Rijn, L., Borst, J., and de Bruijn, J.D. (2010). Dexamethasone treatment during the expansion phase maintains stemness of bone marrow mesenchymal stem cells. J. Tissue Eng. Regen. Med. *4*, 374–386. https://doi.org/10.1002/term.250.

54. da Silva, C.L., Gonçalves, R., Crapnell, K.B., Cabral, J.M.S., Zanjani, E.D., and Almeida-Porada, G.; New Collective Author (2005). A human stromal-based serum-free culture system supports the ex vivo expansion/maintenance of bone marrow and cord blood hematopoietic stem/progenitor cells. Exp. Hematol. *33*, 828–835. https://doi.org/10.1016/j.exphem.2005.03.017.

55. Majumdar, M.K., Thiede, M.A., Mosca, J.D., Moorman, M., and Gerson, S.L. (1998). Phenotypic and functional comparison of cultures of marrow-derived mesenchymal stem cells (MSCs) and stromal cells. J. Cell. Physiol. *176*, 57–66. https://doi.org/10.1002/(SICI)1097-4652(199807)176:1%3C57::AID-JCP7%3E3.0.CO.

56. Mair, F., and Liechti, T. (2021). Comprehensive Phenotyping of Human Dendritic Cells and Monocytes. Cytometry. A. *99*, 231–242. https://doi.org/10.1002/cyto.a.24269.

57. See, P., Dutertre, C.-A., Chen, J., Günther, P., McGovern, N., Irac, S.E., Gunawan, M., Beyer, M., Händler, K., Duan, K., et al. (2017). Mapping the human DC lineage through the integration of high-dimensional techniques. Science *356*, eaag3009. https://doi.org/10.1126/science.aag3009.

58. Constantino, J., Gomes, C., Falcão, A., Cruz, M.T., and Neves, B.M. (2016). Antitumor dendritic cell-based vaccines: lessons from 20 years of clinical trials and future perspectives. Transl. Res. *168*, 74–95. https://doi.org/10.1016/j.trsl.2015.07.008.

59. Kwakkenbos, M.J., Diehl, S.A., Yasuda, E., Bakker, A.Q., van Geelen, C.M.M., Lukens, M.V., van Bleek, G.M., Widjojoatmodjo, M.N., Bogers, W.M.J.M., Mei, H., et al. (2010). Generation of stable monoclonal antibody-producing B cell receptor-positive human memory B cells by genetic programming. Nat. Med. *16*, 123–128. https://doi.org/10.1038/nm.2071.

60. Böttcher, J.P., and Reis e Sousa, C. (2018). The Role of Type 1 Conventional Dendritic Cells in Cancer Immunity. Trends Cancer *4*, 784–792. https://doi.org/10.1016/j.trecan.2018.09.001.

61. Balan, S., O'Brien, L., Peros, A., Wang, X., Leal Rojas, I., McClain, C., Radford, K.J., and Bhardwaj, N. (2025). Harnessing Notch Signaling to Enhance the Generation and Functionality of Human Conventional Type 1 Dendritic Cells for Cancer Immunotherapy Applications. Cancer Immunol. Res. *13*, 1328–1341. https://doi.org/10.1158/2326-6066.CIR-25-0034.

62. Noubade, R., Majri-Morrison, S., and Tarbell, K.V. (2019). Beyond cDC1: Emerging Roles of DC Crosstalk in Cancer Immunity. Front. Immunol. *10*, 1014. https://doi.org/10.3389/fimmu.2019.01014.

63. Gressier, E., Schulte-Schrepping, J., Petrov, L., Brumhard, S., Stubbemann, P., Hiller, A., Obermayer, B., Spitzer, J., Kostevc, T., Whitney, P.G., et al. (2023). CD4+ T cell calibration of antigen-presenting cells optimizes antiviral CD8+ T cell immunity. Nat. Immunol. *24*, 979–990. https://doi.org/10.1038/s41590-023-01517-x.

64. Verdegaal, E.M.E., Visser, M., Ramwadhdoebé, T.H., van der Minne, C.E., van Steijn, J.A.Q.M.J., Kapiteijn, E., Haanen, J.B.A.G., van der Burg, S.H.,

Nortier, J.W.R., and Osanto, S. (2011). Successful treatment of metastatic melanoma by adoptive transfer of blood-derived polyclonal tumor-specific CD4+ and CD8+ T cells in combination with low-dose interferon-alpha. Cancer Immunol. Immunother. *60*, 953–963. https://doi.org/10.1007/s00262-011-1004-8.

65. Verdegaal, E.M.E., Santegoets, S.J., Welters, M.J.P., de Bruin, L., Visser, M., van der Minne, C.E., de Kok, P.M., Loof, N.M., Boekestijn, S., Roozen, I., et al. (2023). Timed adoptive T cell transfer during chemotherapy in patients with recurrent platinum-sensitive epithelial ovarian cancer. J. Immunother. Cancer *11*, e007697. https://doi.org/10.1136/jitc-2023-007697.

66. Jafarzadeh, L., Masoumi, E., Fallah-Mehrjardi, K., Mirzaei, H.R., and Hadjati, J. (2020). Prolonged Persistence of Chimeric Antigen Receptor (CAR) T Cell in Adoptive Cancer Immunotherapy: Challenges and Ways Forward. Front. Immunol. *11*, 702. https://doi.org/10.3389/fimmu.2020.00702.

67. Sharma, P., Hu-Lieskovan, S., Wargo, J.A., and Ribas, A. (2017). Primary, Adaptive, and Acquired Resistance to Cancer Immunotherapy. Cell *168*, 707–723. https://doi.org/10.1016/j.cell.2017.01.017.

68. Ruffell, B., and Coussens, L.M. (2015). Macrophages and Therapeutic Resistance in Cancer. Cancer Cell *27*, 462–472. https://doi.org/10.1016/j.ccell.2015.02.015.

69. Tanaka, A., and Sakaguchi, S. (2019). Targeting Treg cells in cancer immunotherapy. Eur. J. Immunol. *49*, 1140–1146. https://doi.org/10.1002/eji.201847659.

70. van der Leun, A.M., Thommen, D.S., and Schumacher, T.N. (2020). CD8+ T cell states in human cancer: insights from single-cell analysis. Nat. Rev. Cancer *20*, 218–232. https://doi.org/10.1038/s41568-019-0235-4.

71. Philip, M., and Schietinger, A. (2022). CD8+ T cell differentiation and dysfunction in cancer. Nat. Rev. Immunol. *22*, 209–223. https://doi.org/10.1038/s41577-021-00574-3.

72. Mallajosyula, V., Ganjavi, C., Chakraborty, S., McSween, A.M., Pavlovitch-Bedzyk, A.J., Wilhelmy, J., Nau, A., Manohar, M., Nadeau, K.C., and Davis, M.M. (2021). CD8 + T cells specific for conserved coronavirus epitopes correlate with milder disease in patients with COVID-19. Sci. Immunol. *6*, eabg5669. https://doi.org/10.1126/sciimmunol.abg5669.

## STAR★METHODS

### KEY RESOURCES TABLE

| REAGENT or RESOURCE | SOURCE | IDENTIFIER |
|---|---|---|
| Antibodies | | |
| Anti-Human CD1a Alexa Fluor 700 | BioLegend | Cat#300120; clone HI149; RRID: AB_528764 |
| Anti-Human CD1c BUV564 | BD Biosciences | Cat# 750181; clone F10/21A3; RRID: AB_2874384 |
| Anti-Human CD1c PE/Cy7 | eBioscience | Cat#331515; Clone L161; RRID:AB_1953227 |
| Anti-Human CD2 BV605 | BioLegend | Cat# 300223; clone RPA-2.10; RRID:AB_2687242 |
| Anti-Human CD3 BV650 | BioLegend | Cat#317324; clone OKT3; RRID: AB_2563352 |
| Anti-Human CD3 BV480 | BD Biosciences | Cat#566166; clone UCHT1; RRID:AB_2739563 |
| Anti-Human CD3 FITC | eBioscience | Cat# 509366; clone OKT3; RRID:AB_2016669 |
| Anti-Human CD4 BV785 | BioLegend | Cat#317408; clone OKT4; RRID: AB_571951 |
| Anti-Human CD4 PE-Cy7 | BioLegend | Cat#317442; clone OKT4; RRID:AB_2563242 |
| Anti-Human CD5 BUV737 | BD Biosciences | Cat# 748493; clone L17F12; RRID: AB_2872904 |
| Anti-Human CD8 PerCP-Cy5.5 | BioLegend | Cat#344710; clone SK1; RRID:AB_2044010 |
| Anti-Human CD11c BUV737 | BD Biosciences | Cat#568328; clone B-ly6; RRID: AB_3684184 |
| Anti-Human CD11c Alexa Fluro 700 | BioLegend | Cat#337220; clone Bu15; RRID:AB_2561503 |
| Anti-Human CD11c APC-Fire750 | BioLegend | Cat#301646; clone 3.9; RRID: AB_2814122 |
| Anti-Human CD11c APC | BD Biosciences | Cat# 560895; clone B-LY6; RRID: AB_398680 |
| Anti-Human CD14 FITC | BioLegend | Cat#367116; clone 63D3; RRID:AB_2571929 |
| Anti-Human CD14 SparkUV 387 | BioLegend | Cat#399216; clone S18004B; RRID:AB_2922626 |
| Anti-Human CD19 BV650 | BioLegend | Cat# 302238; clone HIB19; RRID: AB_2562097 |
| Anti-Human CD19 FITC | eBioscience | Cat#: 11019182; clone MB19-1; RRID: AB_464965 |
| Anti-Human CD25 PE | BioLegend | Cat# 302606; clone BC96; RRID:AB_314276 |
| Anti-Human CD34 Pacific Blue | BioLegend | Cat#343512; clone 581; RRID: AB_1877197 |
| Anti-Human CD40 BV711 | BioLegend | Cat#334334; clone 5C3; RRID: AB_2564212 |
| Anti-Human CD45 PE-CF594 | BD Biosciences | Cat#562279; clone HI30; RRID: AB_11154577 |
| Anti-Human CD45RA BV510 | BioLegend | Cat# 304142; clone HI100; RRID:AB_2561947 |
| Anti-Human CD45RA BV650 | BioLegend | Cat# 304136; clone HI100; RRID: AB_2563653 |
| Anti-Human CD45RO PE-CF 594 | BD Biosciences | Cat#562299; clone UCHL1; RRID:AB_11154398 |
| Anti-Human CD70 PerCP-Cy5.5 | BioLegend | Cat#355108; clone 113-16; RRID:AB_2562479 |
| Anti-Human CD80 BV785 | BioLegend | Cat#305238; clone 2D10; RRID:AB_2734272 |
| Anti-Human CD83 BV421 | BioLegend | Cat#305324; clone HB15e; RRID:AB_2561829 |
| Anti-Human CD86 BV510 | BioLegend | Cat#305432; clone IT2.2; RRID:AB_2562064 |
| Anti-Human CD88 BV711 | BD Biosciences | Cat# 742319; clone D53-1473; RRID: AB_2874389 |
| Anti-Human CD107α PerCP-Cy5.5 | BioLegend | Cat#328638; clone H4A3; RRID:AB_2565838 |
| Anti-Human CD117 (C-Kit) PE-Cy7 | eBioscience | Cat# 25-1178-42; clone 104D2; RRID: AB_10718535 |
| Anti-Human CD123 PerCP | BioLegend | Cat# 306050; clone 6H6; RRID:AB_2894455 |
| Anti-Human CD141 RB613 | BD Biosciences | Cat# 759328; clone 1A4; RRID: AB_3691416 |
| Anti-Human CD141 PE-Fire810 | BioLegend | Cat# 344133; clone M80; RRID:AB_2927907 |

(Continued on next page)

CellPress
OPEN ACCESS

*Continued*

| REAGENT or RESOURCE | SOURCE | IDENTIFIER |
|---|---|---|
| Anti-Human CD163 PE-Dazzle594 | BioLegend | Cat# 333623; clone GHI/61; RRID: AB_2564200 |
| Anti-Human CD172α (SIRPα) APC | BioLegend | Cat# 372105; clone 15-414; RRID: AB_2650862 |
| Anti-Human CD197(CCR7) PerCP-Cy5.5 | BioLegend | Cat#353220; clone G043H7; RRID:AB_10916121 |
| Anti-Human CD206 PE-Fire700 | BioLegend | Cat# 21770; clone 15-2; RRID:AB_2910389 |
| Anti-Human CD274 (PD-L1) BV711 | BioLegend | Cat# 329722; clone 29E.2A3; RRID: AB_2565764 |
| Anti-Human CD303 BV786 | BD Biosciences | Cat# 748000; clone V24-785; RRID: AB_2872461 |
| Anti-Human CD370 (CLEC9A) BV421 | BD Bioscience | Cat# 564266; clone 3A4; RRID: AB_2738716 |
| Anti-Human CD370 (CLEC9A) PE | BioLegend | Cat# 353803; clone 8F9; RRID: AB_10959651 |
| Anti-Human AXL BV421 | BD Biosciences | Cat# 747864; clone 108724; RRID: AB_2872326 |
| Anti-Human β2m PE | BD Biosciences | Cat# 551337; clone TÜ99; RRID: AB_394152 |
| Anti-Human CXCL9 PE | BioLegend | Cat#357904; clone J1015E10; RRID:AB_2562009 |
| Anti-Human CXCL10 PE | BioLegend | Cat#519503; clone J034D6; RRID: AB_2561408 |
| Anti-Human DLL1 PE | BioLegend | Cat# 346403; clone MHD1-314; RRID: AB_2092809 |
| Anti-Human Granzyme B PE-Cy7 | BioLegend | Cat#372208; clone QA16A02; RRID:AB_2687032 |
| Anti-Human HLA-A2 FITC | BD Biosciences | Cat# 551285; clone BB7.2; RRID: AB_394130 |
| Anti-Human HLA-ABC FITC | BioLegend | Cat#311404; clone W6/32; RRID:AB_314873 |
| Anti-Human HLA-DR Spark Violet 538 | BioLegend | Cat# 20388; clone L243; RRID: AB_2561913 |
| Anti-Human HLA-DR BV605 | BD Biosciences | Cat#562845; clone G46-6; RRID: AB_2744478 |
| Anti-Human HLA-DR RB744 | BD Biosciences | Cat#758140; clone L243; RRID: AB_3690294 |
| Anti-Human HLA-DR APC-Cy7 | BioLegend | Cat#307618; clone L243; RRID:AB_493586 |
| Anti-Human IFNγ FITC | BioLegend | Cat# 506504; clone B27; RRID: AB_315437 |
| Anti-Human IRF8 PE-Cy7 | eBioscience | Cat# 25-9852-82; clone V3GYWCH; RRID: AB_2784675 |
| Anti-Human FLT3 RY703 | BD Biosciences | Cat# 772019; clone 4G8; RRID: AB_3693838 |
| Anti-Human Siglec-6 BV786 | BD Biosciences | Cat# 747909; clone 767329; RRID: AB_2872371 |
| Anti-Human XCR1 PE | BioLegend | Cat# 372603; clone S15046E; RRID: AB_2632710 |
| Anti-Human XCR1 BV421 | BioLegend | Cat#372610; clone S15046E; RRID:AB_2687373 |
| goat anti-mouse IgG(H+L) Alexa Fluor 647 | Invitrogen | Cat#A-21235; RRID: AB_2535804 |
| goat anti-rabbit IgG(H+L) Alexa Fluor 488 | Invitrogen | Cat#A-11008; RRID: AB_143165 |
| TAP1 polyclonal antibody | Bioss Antibodies | Cat#bs-2789R; RRID: AB_10857457 |
| TAP2 polyclonal antibody | Bioss Antibodies | Cat#bs-2374R; RRID: AB_10857127 |
| Anti-Human CD3 | Sanquin | Cat#M1654 clone CLB-T3/4.E |
| Anti-Human CD28 | Sanquin | Cat#M1650 clone CLB-CD28/1 |
| Biological samples | | |
| Human peripheral blood samples | Sanquin | N/A |
| Chemicals, peptides, and recombinant proteins | | |
| Recombinant human IL-2 | Miltenyi Biotec | Cat#130-097-743 |

(*Continued on next page*)

| *Continued* | | |
|---|---|---|
| REAGENT or RESOURCE | SOURCE | IDENTIFIER |
| Recombinant human IL-4 | ImmunoTools | Cat# 11340043 |
| Recombinant human IL-7 | Miltenyi Biotec | Cat#130-093-937 |
| Recombinant human IL-15 | Miltenyi Biotec | Cat#130-095-760 |
| Recombinant human IL-21 | Miltenyi Biotec | Cat# 130-095-767 |
| Recombinant human Flt3-Ligand | Miltenyi Biotec | Cat#130-093-854 |
| Recombinant human M-CSF | Miltenyi Biotec | Cat#130-093-963 |
| Recombinant human TPO | Miltenyi Biotec | Cat#130-094-013 |
| Recombinant human SCF | Miltenyi Biotec | Cat#130-096-692 |
| Recombinant human GM-CSF | Miltenyi Biotec | Cat#130-095-372 |
| AF488 or APC-conjugated HLA-A2/MART-$1_{26\text{-}35}$ tetramers | Peptide facility in LUMC | N/A |
| APC or PE-conjugated HLA-A2/$CDK4_{23\text{-}32}$ tetramers | Peptide facility in LUMC | N/A |
| PE-conjugated HLA-A2/MAGE3 tetramers | Peptide facility in LUMC | N/A |
| MART-$1_{26\text{-}35}$ short peptide | Peptide facility in LUMC | N/A |
| MART-$1_{15\text{-}40}$ long peptide | Peptide facility in LUMC | N/A |
| IMDM | Gibco | Cat#12440053 |
| RPMI-1640 | Gibco | Cat#11875093 |
| Fetal calf serum (FCS) | Serana | Cat#S-FBS-SA-010 |
| Trypsin | Gibco | Cat#11560626 |
| β-mercaptoethanol | Sigma-Aldrich | Cat#M6250 |
| Penicillin/streptomycin | Gibco | Cat#15140122 |
| Dimethyl sulfoxide (DMSO) | Dimethyl sulfoxide | Cat#472301 |
| CellTrance Violet(CTV) | Invitrogen | Cat#C34557 |
| 7-amino-actinomycin D (7AAD) | eBioscience | Cat#00-6993-50 |
| Zombie Red Fixable Viability Kit | BioLegend | Cat#423109 |
| Zombie UV Fixable Viability Kit | BioLegend | Cat#423107 |
| Near-IR Dead Cell Stain Kit | Invitrogen | Cat#L10119 |
| UltraComp eBeads™ Compensation Beads | Invitrogen | Cat#01-2222-42 |
| CD14 magnetic MicroBeads | Miltenyi Biotec | Cat#130-097-052 |
| CD19 magnetic MicroBeads | Miltenyi Biotec | Cat#130-050-301 |
| eBioscience™ Foxp3 / Transcription Factor Staining Buffer Set | Invitrogen | Cat#00-5523-00 |
| Protein Transport Inhibitor (BD GolgiPlug) | BD Biosciences | Cat#555029 |
| BD Cytofix/Cytoperm kit | BD Biosciences | Cat#554714 |
| Buffer RLT | QIAGEN | Cat#79216 |
| TRAIL | Sigma | Cat#T9701 |
| FASL | AdipoGen | Cat#AG-40B-0001-C010 |
| Phorbol 12-myristate 13-acetate (PMA) | Sigma | Cat#79346 |
| Ionomycin | InvivoGen | Cat#inh-ion |
| LPS | InvivoGen | Cat#tlrl-peklps |
| poly(I:C) HMW | InvivoGen | Cat#tlrl-pic |
| R848 | InvivoGen | Cat#tlrl-r848 |
| Caspase-3/7 Green Apoptosis Reagent | Satorius | Cat#4440 |
| 4% PFA | Sigma | Cat#1.00496 |
| Red blood cell lysis buffer | Santa Cruz Biotechnology | Cat#sc-296258 |
| Ficoll-paque™ Plus | Cytiva | Cat#17-1440-02 |
| nCounter host response panel | Nanostring | N/A |
| Experimental models: Cell lines | | |
| Melanoma526 | ATCC | RRID:CVCL_8051 |

(*Continued on next page*)

*Continued*

| REAGENT or RESOURCE | SOURCE | IDENTIFIER |
|---|---|---|
| Software and algorithms | | |
| GraphPad Prism (v 10.2.3) | GraphPad software | https://www.graphpad.com |
| FlowJo$^{TM}$ software (v 10.10.0) | BD Biosciences | https://www.flowjo.com/solutions/flowjo/downloads |
| OMIQ flow cytometry software | Dotmatics | https://www.omiq.ai/ |
| nSolver advanced analysis module (v 1.1.4) | Nanostring | https://nanostring.com/products/analysis-solutions/ncounter-analysis-solutions/nsolver-data-analysis-support/ |
| Incucyte S3 software (v2018B) | Sartorius | https://www.sartorius.com/ |
| limma R packages (v 3.58.1) | Ritchie ME et al. Nucleic Acids Res. 2015 | https://bioconductor.org/packages/limma |
| fgsea R packages (v 1.28.0) | Korotkevich et al., bioRxiv, 2019 | https://bioconductor.org/packages/fgsea |
| ggplot2 R packages (v 3.5.1) | ggplot2: Elegant Graphics for Data Analysis, 2016 | https://ggplot2.tidyverse.org |
| pheatmap T package (v1.0.12) | CRAN | https://cran.r-project.org/package=pheatmap |
| R (v 4.1.2) | CRAN | https://www.r-project.org/ |

## EXPERIMENTAL MODEL AND STUDY DETAILS

### Human peripheral blood samples

Buffy coats were obtained from adult healthy donors after written informed consent at Sanquin, in accordance with the Declaration of Helsinki and Dutch regulations for the use of human materials, and were anonymized before release to the researchers. PBMC were isolated from buffy coats using Ficoll-Paque Plus (GE Healthcare). HPC, monocytes and B cells were isolated from HLA-A2$^{+}$ donors, while CD4$^{+}$ and CD8$^{+}$ T cells were used regardless of HLA type and donor origin. Donor matching varied by assay and is specified in the relevant method sections.

### Feeder cells

OP9-hDLL1 cells were generated by retroviral transduction of OP9 cells with hDLL1 construct. MSC were isolated from healthy donors' bone marrow.[52] CD40L-L cells were generated by transduction of mouse L cell fibroblasts with CD40L construct.

## METHOD DETAILS

### *In vitro* generation of blood progenitor-derived DC

Human MSC, OP9, and OP9-hDLL1 were harvested using 0.05% trypsin. MSC were irradiated at 16 Gy, and OP9/OP9-hDLL1 at 30 Gy, prior to plating as feeder cells. OP9 and OP9-hDLL1 cells were mixed at a 3:1 ratio for culture method 1 and 2 (Figure S1A**)**. MSC and OP9-hDLL1 were mixed at a 3:1 ratio for culture method 3. Feeder cells were then seeded at 3000 cells/well in round-bottom 96-well plates in Iscove's Modified Dulbecco's Medium (IMDM) supplemented with 10% fetal calf serum (FCS), 100 U/mL penicillin, 100 μg/mL streptomycin, and 50 μM β-mercaptoethanol. After 24 h, the feeder cell layer was gently washed with the same medium to remove non-adherent cells, and CD3$^{-}$CD19$^{-}$CD34$^{+}$c-KIT$^{+}$ HPC (progenitors, 1000 cells/well), sorted from PBMC, were seeded on top of the feeder cells in the presence of the indicated cytokine cocktails. For culture method 1, freshly sorted progenitors were initially seeded at 1000 cells/well without feeders, with cytokines, and whole well transferred to the OP9/OP9-hDLL1 feeder layer after 7 days. DC differentiation cytokines were used at final concentrations of 100 ng/mL FLT3L, 5 ng/mL each of TPO, SCF and GM-CSF, 1 ng/mL of IL-7 and 0.5 ng/mL M-CSF. For experiments comparing all different culture methods, cytokines were present throughout the culture period as indicated in Figure S1A. Half of the medium was refreshed every 6-7 days with fresh cytokine-containing medium. Cultures were maintained at 37°C with 5% $CO_2$ for 14–21 days before analysis. Generated progenitor-derived DCs were used for phenotypic, functional, and co-culture assays as indicated.

### Flow cytometry staining and cell sorting

For sorting HPC, CD4$^{+}$ T cells and CD8$^{+}$ T cells, CD19$^{+}$ cells were depleted from PBMC using CD19 MicroBeads (Miltenyi Biotec). Cells were then incubated in BD staining buffer with fluorochrome-conjugated monoclonal antibodies (mAb) at 4°C for 45 min and sorted on a BD FACS Aria III. HPC were defined as CD3$^{-}$CD19$^{-}$CD34$^{+}$c-KIT$^{+}$ cells. Naïve CD4$^{+}$ T cells as CD3$^{+}$HLA-DR$^{-}$CD4$^{+}$CD25$^{-/low}$ CD45RA$^{+}$. Naïve CD8$^{+}$ T cells as CD3$^{+}$HLA-DR$^{-}$CD8$^{+}$CD25$^{-/low}$ CD45RA$^{+}$ and total CD8$^{+}$ T cells as CD3$^{+}$HLA-DR$^{-}$CD8$^{+}$ cells.

For flow cytometry analysis, cells were stained with mAbs against surface and intracellular markers, or tetramers. Distinct antibody panels (Table S5) and related reagents (key resources table) were used to characterize DC phenotype, DC activation, and T-cell priming. For intracellular staining, DC were incubated with GolgiPlug (1:1000, BD Biosciences) ), whereas CD8$^{+}$ T cells were stimulated for

3 h with 50 ng/ml PMA, 1 μg/ml Ionomycin, and GolgiPlug (1:1000, BD Biosciences). Cells were subsequently fixed and permeabilized using the Cytofix/Cytoperm kit (BD Biosciences). TAP1/2 detection required secondary antibody staining, validated by fluorescence minus one (FMO) controls. Dead cells were excluded using viability dye. Compensation beads, unstained cells, single stained cells and FMO were included for compensation and gating. Data acquisition was performed on BD LSR Fortessa™, Cytek Aurora spectral flow cytometers or SONY ID7000™ Spectral Cell Analyzer and analyzed using FlowJo™ or OMIQ software.

### NanoString nCounter gene expression analysis

Progenitor-derived DC were cocultured with $CD4^+$ T cells for 12 h, then sorted using HLA-DR, CD3, and 7-AAD markers**.** Cells were lysed in 5-6 μl of 1/3 diluted RLT buffer (QIAGEN) at 2000 cells/μl and then hybridized with probes according to NanoString nCounter's user manual for whole cell lysates. Samples were analyzed with the NanoString nCounter MAX platform using the Human Host Response Panel (773 genes, Table S1). RNA counts were normalized with nSolver (v1.1.4) and log2-transformed data were analyzed in R (v4.1.2). DEGs (Table S2**)** were identified using 'limma' ($p < 0.05$, log2FC > 1 or < –1). Volcano plots and heatmaps were generated in R (v4.1.2) using 'ggplot2' and 'pheatmap'. The cDC1 "help" signature from a prior study[19] was used (Tables S3 and S4). Reactome pathway enrichment was performed with 'fgsea' (v1.28.0).

### Tumor antigen-specific CTL priming platform

This method was adapted from our previous publication[19], $CD8^+$ T cells were either retrovirally transduced to express the MART-$1_{26\text{-}35}$/HLA-A2-specific TCR, or used as polyclonal population. $CD8^+$ T cells were cultured in RPMI-1640 with 10% FBS and human IL-2/IL-7/IL-15 (each at 10 ng/ml) before use. Naïve $CD4^+$ T cells were cultured in the presence of agonistic mAbs against CD3 (0.1 μg/ml) and CD28 (0.2 μg/ml) for 24-36 h to induce $CD4^+$ T-cell activation. MART-$1_{26\text{-}35}$ short peptide (SP, ELAGIGILTV, 500 ng/ml), MART-$1_{15\text{-}40}$ long peptide (LP, KGHGHSYTTAEELAGIGILTV, 20 μg/ml) or debris of MART-1 expressing Mel526 cells was used as antigen source. Mel526 cell debris was prepared as previously described.[19]

HLA-$A2^+$ progenitor-derived DC were co-cultured with either activated or naïve $CD4^+$ T cells at a 1:1 ratio for 2 h IMDM supplemented in 1% FBS, followed by adding tumor antigen along with PRR stimuli consisting of 50 ng/ml LPS (InvivoGen), 20 μg/ml poly I:C (InvivoGen) and 3 μg/ml R848 (InvivoGen). To trace proliferation, $CD8^+$ T cells were labeled with CTV before being addition to the co-culture platform. After 12-16 h, culture supernatants were removed and CTV-labeled $CD8^+$ T cells were added at a 1:10 DC: T-cell ratio. Cultures were maintained for 6-7 days in RPMI-1640 supplemented with 10% FCS, 2 mM L-glutamine and IL-7/IL-15 (each 0.2 ng/ml). Cells were then harvested, stained, and analyzed by flow cytometry. All reagents are listed in key resources table.

### Antigen presenting cell comparison for tumor specific CTL detection and expansion

B cells were isolated from HLA-$A2^+$ PBMC by positive selection using CD19 MicroBeads (Miltenyi Biotec), followed by isolation of monocytes from the remaining fraction using CD14 MicroBeads (Miltenyi Biotec), according to the manufacturer's instructions. Purified $CD19^+$ B cells ($2 \times 10^6$ per well) were cultured for 7 days on irradiated (55 Gy) CD40L-expressing fibroblasts[60] ($5 \times 10^5$ per well) in 6-well plates in the presence of IL-21 (25 ng/mL). Purified $CD14^+$ monocytes ($5 \times 10^6$ per well) were cultured for 7 days in 6-well plates in the presence of GM-CSF (100 ng/mL) and IL-4 (50 ng/mL) to generate moDC.[19] Cells were cultured in complete IMDM supplemented with 10% FCS, 100 U/mL penicillin, 100 μg/mL streptomycin and 50 μM β-mercaptoethanol at 37°C and 5% $CO_2$, and cytokine-containing medium was refreshed on day 4. Presence of activated B cells and moDC in the cultures was assessed by flow cytometry on day 7.

To compare APC function, progenitor-derived DC, activated B cells and moDC obtained from the same donors (n=3) were plated at 100,000 cells per well in 48-well plates and co-cultured with either activated or naïve $CD4^+$ T cells at a 1:1 ratio for 2 h in IMDM supplemented with 1% FBS. Subsequently, Mel526 cell debris and PRR stimuli were added as described in the previous section. After 12-16 h, culture supernatants were removed, and CTV-labeled polyclonal $CD8^+$ T cells were added at an APC:T cell ratio of 1:10. Cultures were maintained for 6 days in RPMI-1640 supplemented with 10% FCS, 2 mM L-glutamine, and IL-7 and IL-15 (0.2 ng/mL each). MART1-specific $CD8^+$ T-cell responses were assessed by flow cytometry using MART-1/HLA-A2 tetramer staining, in combination with Granzyme B staining and CTV dilution.

### Allogeneic mixed lymphocyte reaction

Progenitor-derived DC from donors X1-X3 were co-cultured with allogeneic activated or naïve $CD4^+$ T cells from donors Y1-Y3 at a 1:1 ratio for 2 h in complete IMDM containing 1% FBS. Subsequently, CTV-labeled allogeneic naïve $CD8^+$ T cells from the same donors (Y1–Y3) were added at a DC:T cell ratio of 1:10. Cultures were maintained for 6 days in RPMI-1640 supplemented with 10% FCS, 2 mM L-glutamine, and low-dose IL-7 and IL-15 (0.2 ng/mL each) to support T cell survival. Naïve $CD8^+$ T-cell priming was assessed by flow cytometry based on CTV dilution.

### IncuCyte T-cell killing assay

Mel526 cells were seeded at 5000 single cells/well in 96-well plates. The next day, medium was removed and sorted $CD8^+$ T cells were added at a 4:1 effector: target ratio and IncuCyte Caspase-3/7 Green Apoptosis Reagent (20 μM, Sartorius) in culture medium. Plates were incubated for 30 min at room temperature and next imaged in the IncuCyte ZOOM® (Sartorius) every 2 h for 24 h.

CellPress
OPEN ACCESS

Afterwards, suspend cells were collected for flow cytometry; remaining Mel526 cells were fixed with 4% PFA (Sigma), stained with crystal violet, and imaged by CCD microscopy (Zeiss Axio Imager Z1). Data analysis was done using IncuCyte S3 Software (v2018B).

## QUANTIFICATION AND STATISTICAL ANALYSIS

Data were analyzed with GraphPad Prism 9.0 using Student's t-test (paired or unpaired), one-way ANOVA or two-way ANOVA, followed by Tukey's multiple comparisons test for group comparisons. All data are presented as means ± SEM or SD; $^{*}p < 0.05$; $^{**}p < 0.01$; $^{***}p < 0.001$; $^{****}p < 0.0001$, $p > 0.05$ not shown.

Cell Reports Methods, Volume *6*

## Supplemental information

# Efficient generation of human cDC1-like cells to detect and enhance tumor-reactive T cells via $CD4^+$ T cell help

**Yizhi Wang (汪逸之), Xin Lei (雷馨), Tom de Wit, Ellen Schrama, Marij J.P. Welters, Karel van Leeuwen, Sander de Kivit, Miriam de Boer, Jitske van den Bulk, Noel F.C. C. de Miranda, Ton N. Schumacher, Chong Sun (孙崇), Jannie Borst, and Yanling Xiao (肖艳灵)**

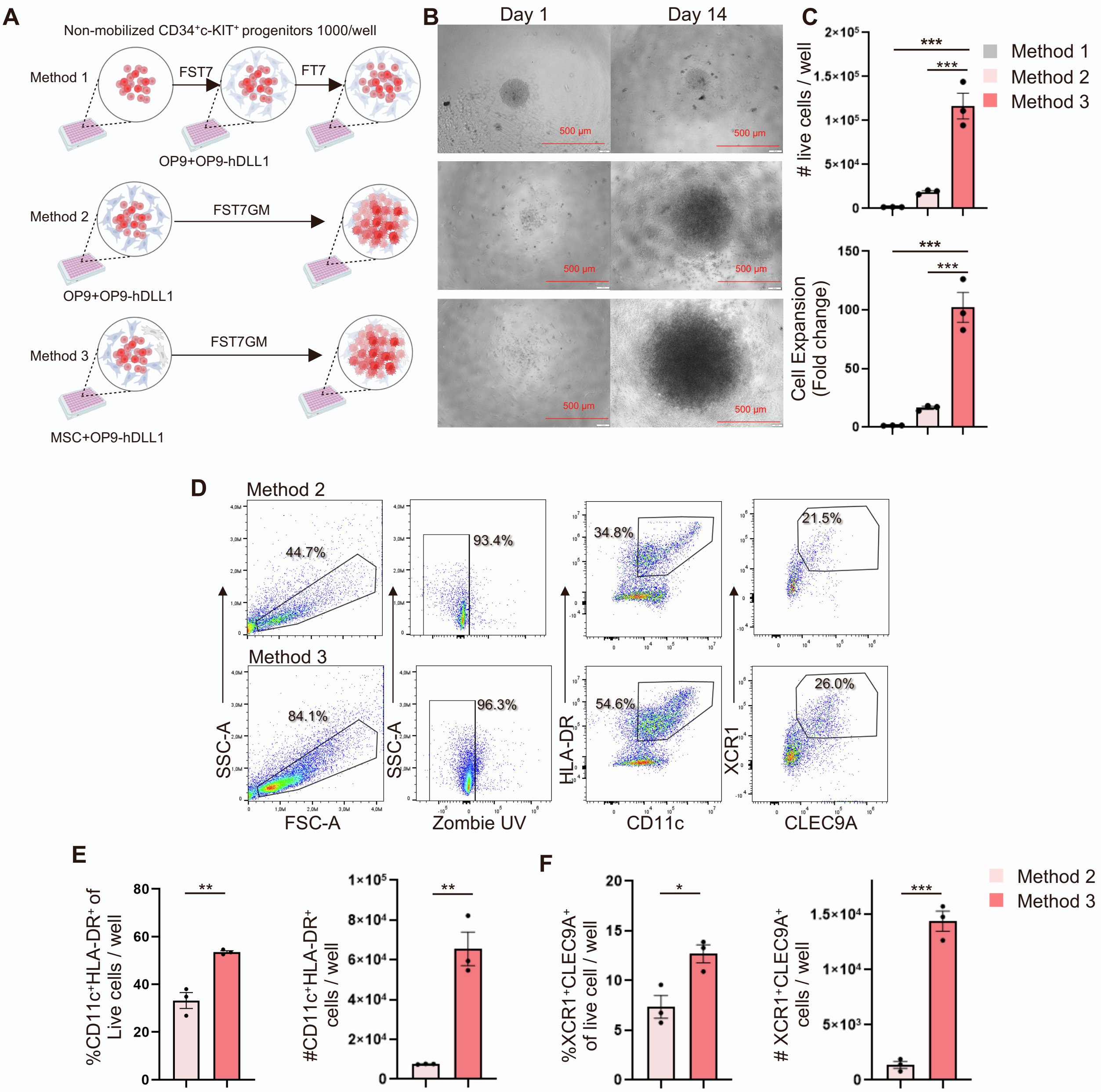


**Figure S1. Optimizing DC generation from non-mobilized PBMC progenitors, related to Figure 1.**
DC generation from non-mobilized PBMC-derived CD34⁺c-KIT⁺ progenitors were compared under various conditions.
(**A**) Schematic representation of three co-culture strategies for *in vitro* DC differentiation. Method 1 follows protocol established by Balan *et al.*[1], using OP9+OP9-hDLL1 feeder layers and sequential cytokine cocktails. Method 2 uses the same feeder composition but uses a single cytokine mix throughout the procedure. Method 3 incorporates a modified feeder composition (human MSC+OP9-hDLL1) along with a single cytokine mix. For clarity, the cytokines are as follows: F (Flt3L), S (SCF), T (TPO), 7 (IL-7), G (GM-CSF), M (M-CSF).
(**B**) Microscopic pictures depicting the growth of progenitor-derived DC formed on Day 1 and Day 14 in each culture condition (scale bar: 500 μm).
(**C**) Quantification by counting of total live cells per well (up) and fold expansion of total cells (below) after 14 days of culture.
(**D**) Representative flow cytometry plots showing gating strategy for method 2 and method 3. Note: method-1 did not generate sufficient cells for flow cytometry analysis.
(**E**) % Frequency among live cells (left) and # absolute number (right) of $CD11c^+HLA\text{-}DR^+$ cells per well.
(**F**) % Frequency among live cells (left) and # absolute number (right) of $XCR1^+CLEC9A^+$ cells per well.
Data are shown in C-F (n=3) as mean ± SEM, with each dot representing one donor. *$p<0.05$, **$p<0.01$, ***$p<0.001$ by one-way ANOVA with Tukey's multiple comparisons in C, two-sided unpaired Student's t-test in E,F.

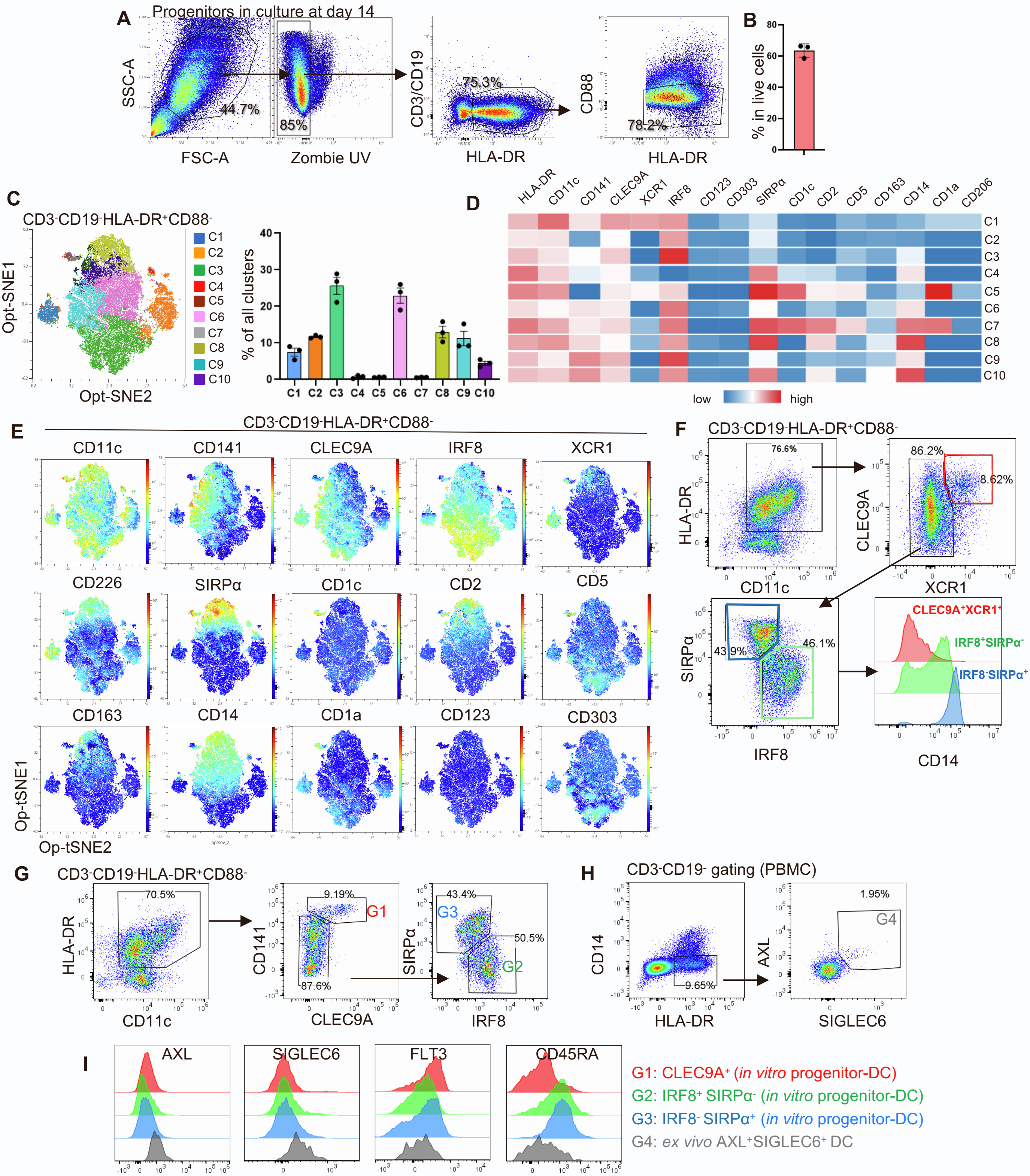


**Figure S2. Generating human cDC1-like cells from non-mobilized blood-derived progenitors *in vitro,* related to Figure 1.**

Phenotypic analysis was performed to assess heterogeneity within the CD3⁻CD19⁻HLA-DR⁺CD88⁻ DC compartment.

(**A**) Gating strategy for progenitor-derived DC phenotype analysis.

(**B**) Quantification of frequencies (%) of CD3⁻CD19⁻HLA-DR⁺CD88⁻ DC progeny in live cells.

(**C**) Opt-SNE plot (left) of 10 clusters identified within CD3⁻CD19⁻HLA-DR⁺CD88⁻ progeny cells and associated frequencies (%, right) of 10 clusters in HLA-DR⁺CD88⁻ DC progeny.

(**D**) Heatmap of median expression values of key phenotypic markers identifying cDC1-like and other DC types.

(**E**) Opt-SNE plots depicting expression of indicated markers on CD3⁻CD19⁻HLA-DR⁺CD88⁻ population.

(**F**) Gating strategy used to define populations corresponding to clusters C1, C3, C6 and C9 identified by Opt-SNE plots, and representative histogram showing CD14 expression for CLEC9A⁺XCR1⁺, SIRPα⁻IRF8⁺ and SIRPα⁻IRF8⁺ subpopulations.

(**G**) Subpopulation gating strategy of progenitor-derived DC to assess the expression of pre-DC associated markers.

(**H**) Gating strategy for identifying AXL⁺SIGLEC6⁺ pre-DC populations in *ex vivo* blood samples.

(**I**) Representative histogram showing expression of AXL, SIGLEC6, FLT3 and CD45RA in progenitor-derived DC.

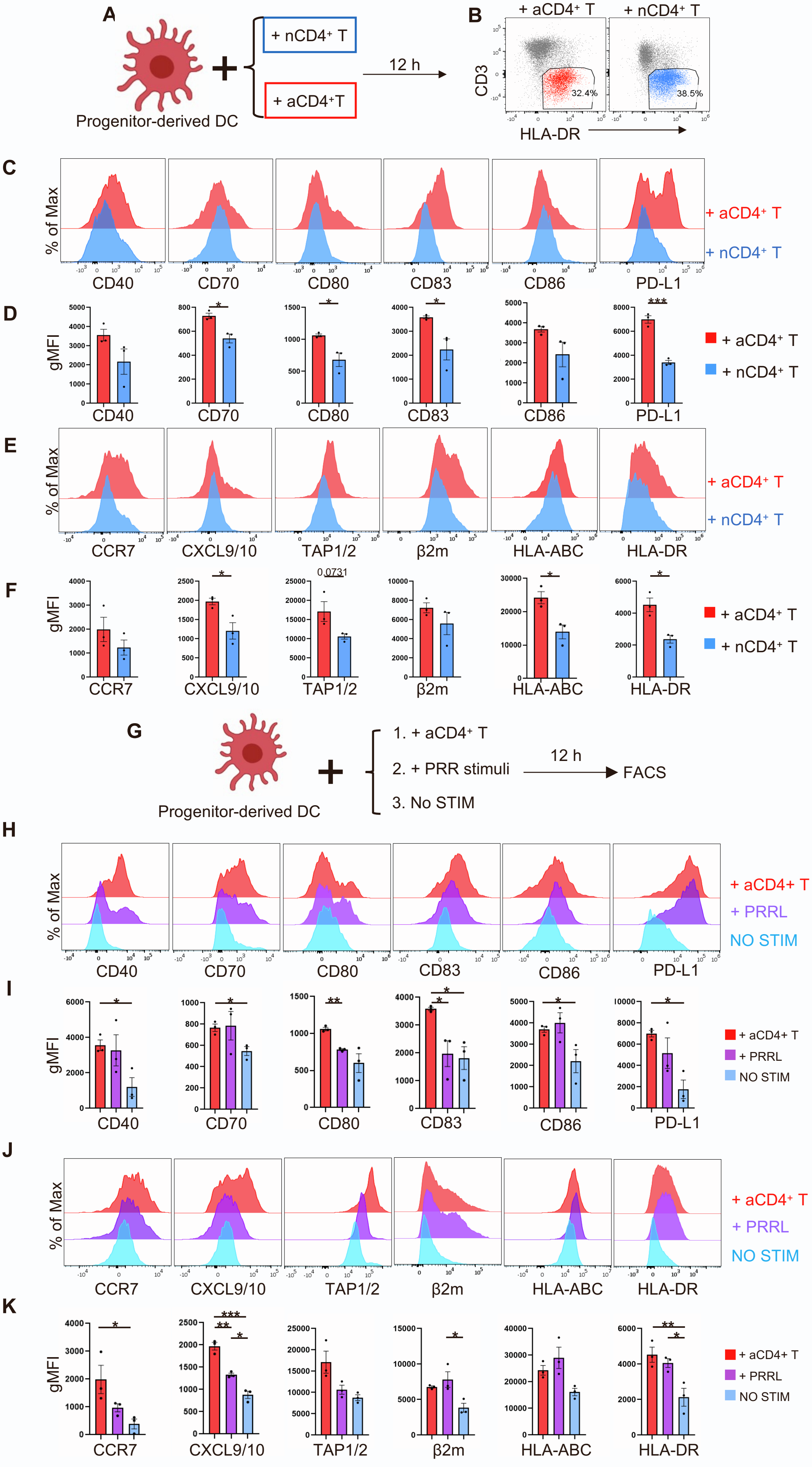

A
Progenitor-derived DC
+ nCD4⁺ T
+ aCD4⁺T
12 h
B
+ aCD4⁺ T
+ nCD4⁺ T
CD3
HLA-DR
32.4%
38.5%
C
% of Max
CD40
CD70
CD80
CD83
CD86
PD-L1
+ aCD4⁺ T
+ nCD4⁺ T
D
gMFI
E
CCR7
CXCL9/10
TAP1/2
β2m
HLA-ABC
HLA-DR
F
0.0731
G
1. + aCD4⁺ T
2. + PRR stimuli
3. No STIM
FACS
H
+ aCD4+ T
+ PRRL
NO STIM
I
J
K

**Figure S3. Response of progenitor-derived cDC1-like cells to activated CD4⁺ T cells, related to Figure 2**.
Progenitor-derived DCs were co-cultured overnight either with activated (a) or naïve (n) $CD4^+$ T cells, or under the indicated stimulation conditions including activated $CD4^+$ T cells or PRR stimuli. Next, key molecules of the cDC1 "help" signature[2] were analyzed by flow cytometry.
(**A**) Experimental design for **C**-**F**.
(**B**) Gating strategy for flow cytometric analysis of the $CD3^-$HLA-$DR^+$ progenitor-derived DC population after co-culture.
(**C-F**) Histograms (**C**, **E**) and geometric mean fluorescence intensity (gMFI) quantifications (**D**, **F**) for indicated markers expressed by progenitor-derived DC under indicated conditions.
(**G**) Experimental design for **H**-**K**.
(**H**-**K**) Histograms (**H** and **J**) and gMFI quantifications (**I** and **K**) for indicated markers expressed by progenitor-derived DC under indicated conditions.
Data are pooled from three (n=3) independent donors (D, F, I, K). Data are shown as means $\pm$ standard error of mean (SEM). *p<0.05, **p<0.01, ***p<0.001. Statistics analyses were performed using a two-sided unpaired Student's t-test for panels (D) and (F), one-way ANOVA with Tukey's multiple comparisons for panels (I) and (K).

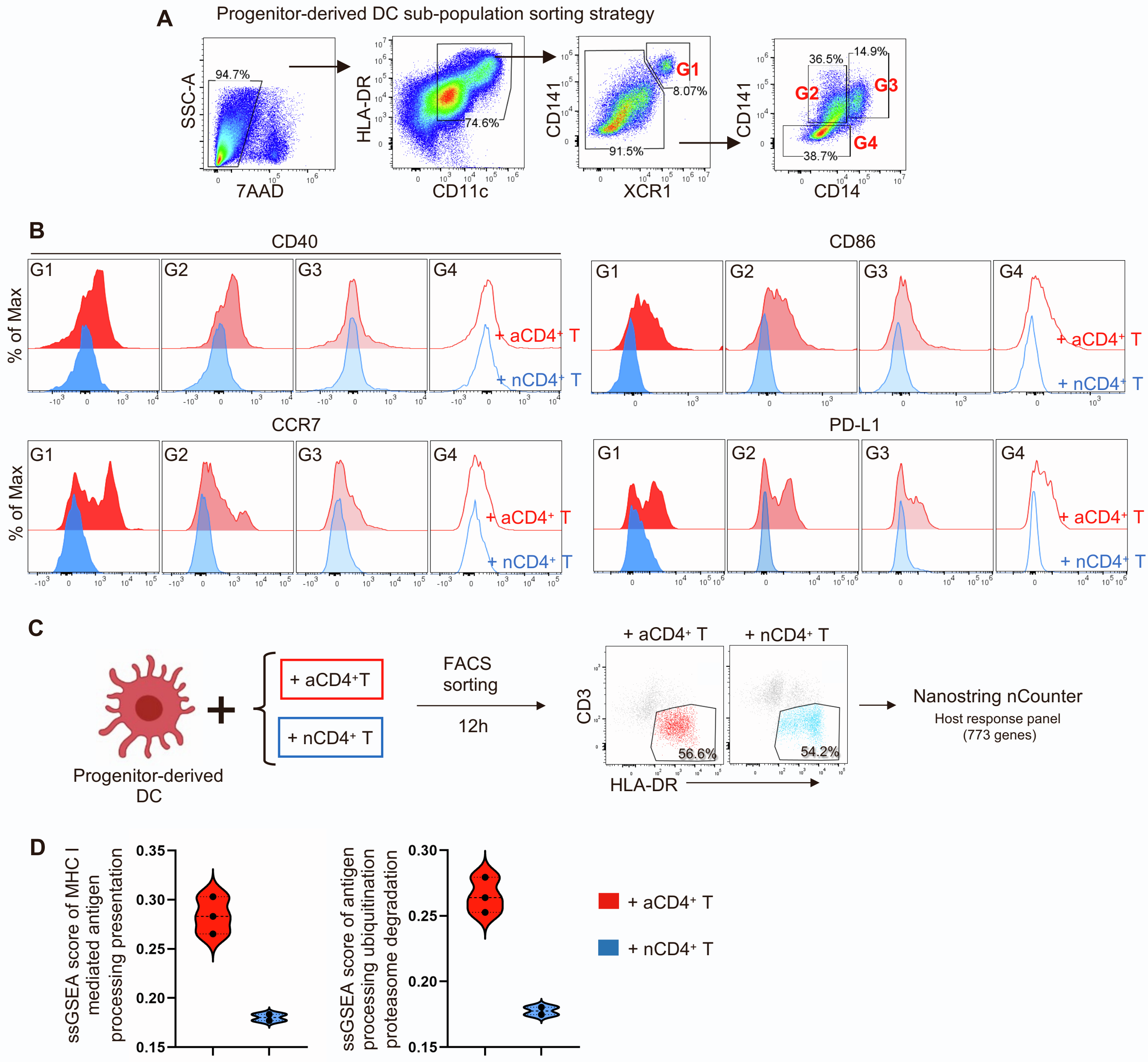


**Figure S4. Responses of sorted progenitor-derived DC to activated CD4⁺ T cells, related to Figure 2 and 3.** Sorted progenitor-derived DC populations were examined for their response to activated CD4⁺ T-cell help. A and B are associated to Figure 2, C and D are associated to Figure 3.

(**A**) Gating strategy for sorting the four progenitor-derived DC populations.

(**B**) Representative histograms of CD40, CD86, CCR7 and PD-L1 on the corresponding sorted progenitor-derived DC populations under the indicated conditions.

(**C**) Gating strategy for sorting progenitor-derived DC used in the NanoString nCounter analysis

(**D**) Single-sample gene set enrichment analysis (ssGSEA) was used to assess pathway activity. Violin plots show the distribution of ssGSEA scores for the class I MHC antigen presentation pathway and the ubiquitination/proteasome degradation pathway in progenitor-derived DC co-cultured with activated (a)CD4⁺ and naïve (n)CD4⁺ T cells. Each dot represents one sample.

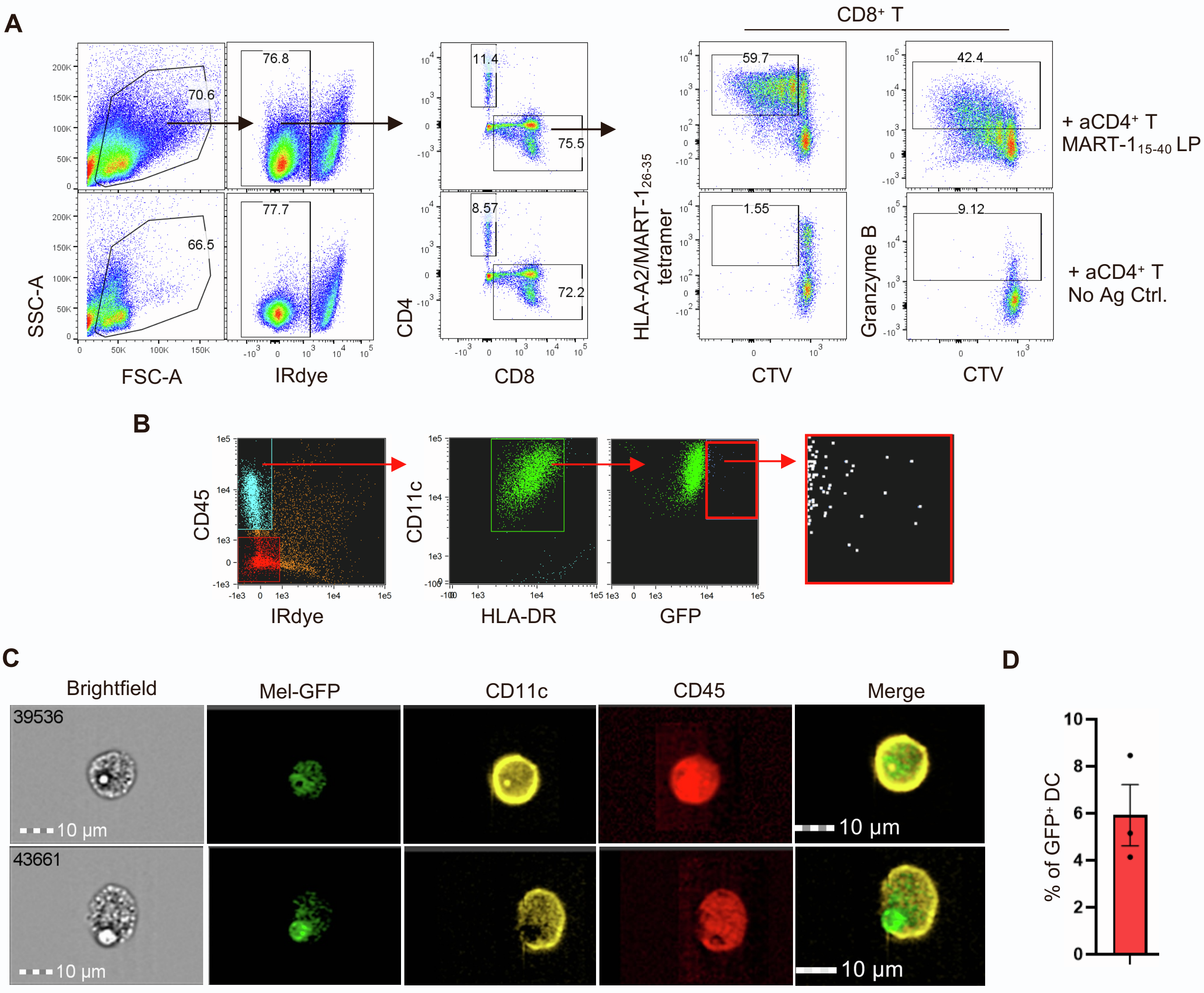


**Figure S5. Progenitor-derived DC have the ability to relay CD4⁺ T-cell help for CTL responses, related to Figure 4.**
(**A**) Gating strategies for detecting CD8⁺ T-cell responses in our *in vitro* cytotoxic T-cell priming system based on CTV dilution and intracellular Granzyme B staining. CD8⁺ T cells had been transduced to express the HLA-A2/MART-$1_{26\text{-}35}$-specific TCR before being added to priming system.
(**B-D**) Imaging flow cytometry was performed to analyze the phagocytotic capacity of progenitor-derived DC.
(**B**) Gating strategy for analyzing phagocytotic capacity of progenitor-derived DC response.
(**C**) Representative brightfield and fluorescent images of CD11c⁺CD45⁺ progenitor-derived DC phagocytosing GFP⁺ melanoma cell debris.
(**D**) Quantification of % of GFP⁺ cells in total live DC. Data are pooled from three (n=3) independent donors in D, and are shown as means ± SEM.

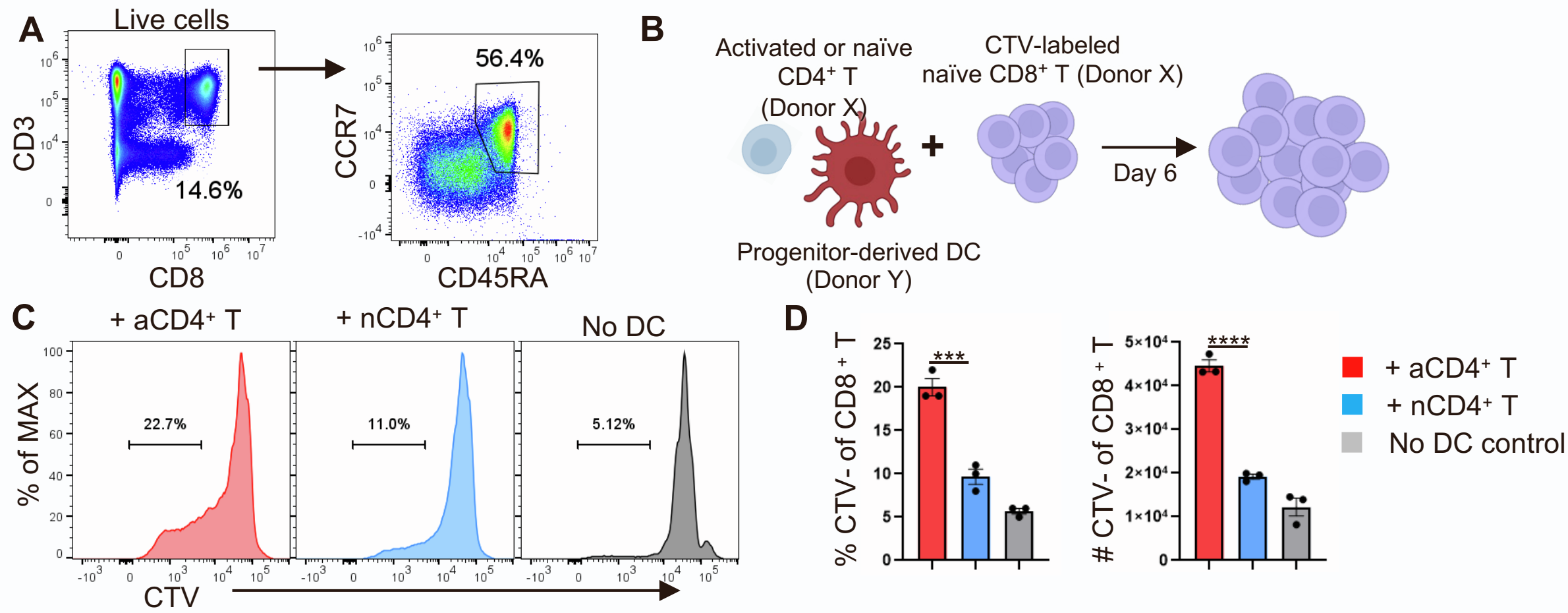


**Figure S6. Progenitor-derived DC prime naïve CD8⁺ T cells in an allogeneic MLR, related to Figure 5.**
The capacity of progenitor-derived DC to prime naïve CD8⁺ T cells was assessed using an allogeneic mixed lymphocyte reaction. Approximately 10,000 progenitor-derived DC from donors Y1-Y3 (n=3) were co-cultured with an equal number of allogeneic activated (a) or naïve (n) CD4⁺ T cells from donors X1-X3 (n=3), together with ~100,000 naïve CD8⁺ T cells from the same donors (X1-X3), to evaluate the effect of CD4⁺ T cell help on naïve CD8⁺ T-cell priming.
(**A**) Gating strategy for sorting naïve CD8⁺ T cells.
(**B**) Schematic illustration of the MLR experimental design.
(**C**) Representative CTV histograms showing proliferation of naïve CD8⁺ T cells under indicated conditions.
(**D**) Frequency (%) and absolute number (#) of CTV⁻ CD8⁺ T cells among total CD8⁺ T cells. Data are pooled from three (n=3) independent donors in **D** and shown as means ± SEM. ***p<0.001, ****p<0.0001(One-way ANOVA with Tukey's multiple comparisons).

**A**

| *Peptide ID* | *Peptide sequence* | *HLA allele* | *Fluorochrome* |
|---|---|---|---|
| MART-1$_{25\text{-}36}$ | ELAGYGILTV | HLA-A0201 | AF488 & APC |
| CDK4$_{23\text{-}32}$ | ALDPHSGHFV | HLA-A0201 | APC & PE |
| MAGE3 | FLWGPRALV | HLA-A0201 | PE |

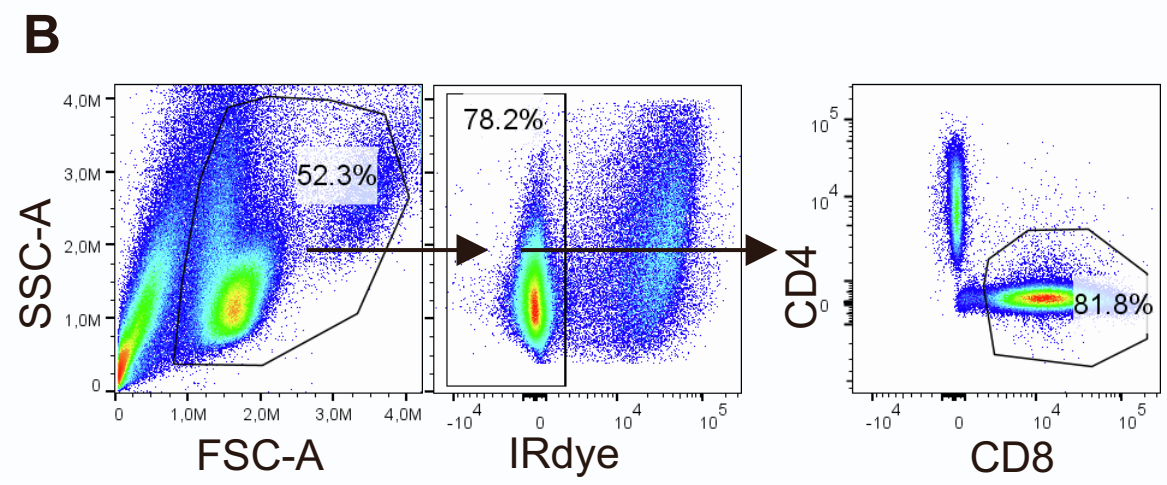


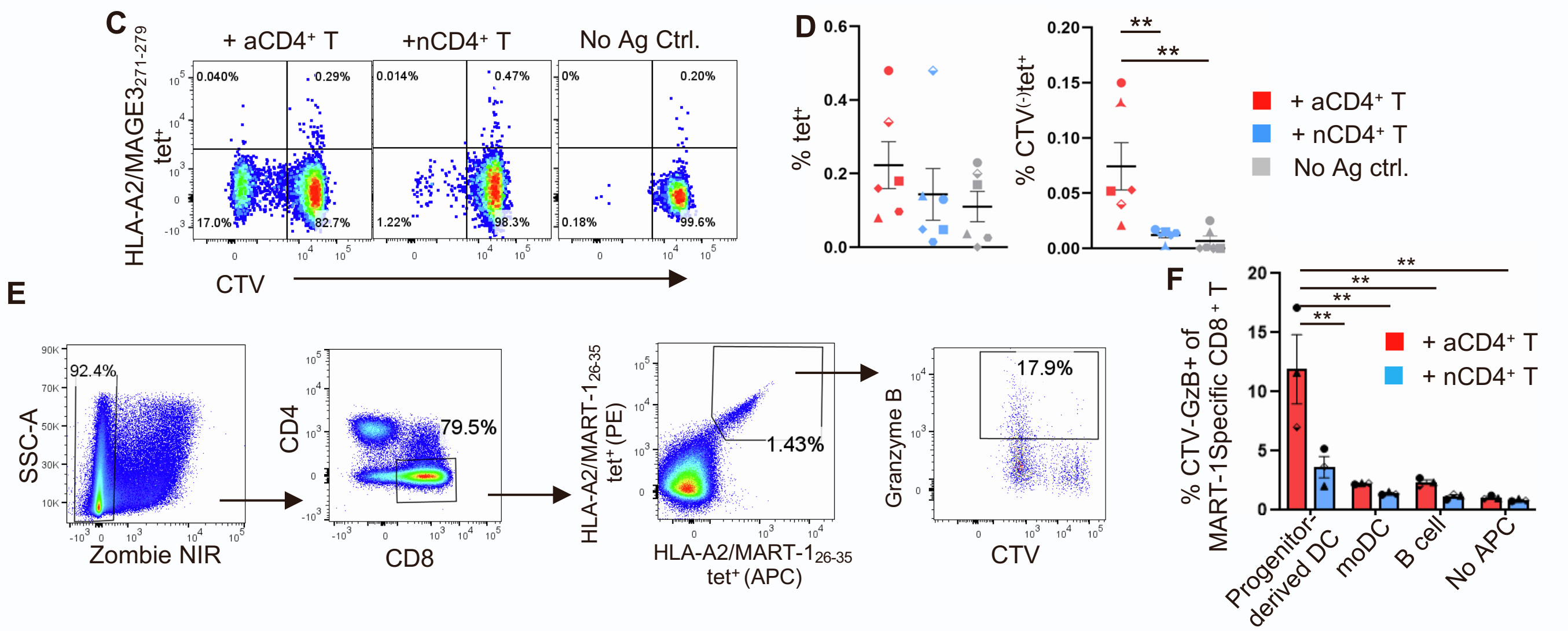


**Figure S7. Helped progenitor-derived DC efficiently detect tumor antigen-specific CD8⁺ T cells in blood and outperform moDC and B cells, related to Figure 5 and 6.**
(**A**) Overview of the tetramers used in the experiment(s).
(**B**) Gating strategy for detecting tumor antigen-specific CD8⁺ T cells using tetramers.
(**C**) Flow cytometric plots depicting CD8⁺ T cells specific for HLA-A2/MAGE3$_{271\text{-}279}$.
(**D**) Quantification % of MAGE3$_{271\text{-}279}$-specific population within CD8⁺ T cells (left), and # of MAGE3$_{271\text{-}279}$-specific CD8⁺ T cells (right).
(E-F) Progenitor-derived DC loaded with dead Mel526 cell debris were used to detect endogenous MAGE3$_{271\text{-}279}$-specific CD8⁺ T cells from blood in the presence of activated (a) or naïve (n) CD4⁺ T cells. Next, progenitor-derived DC, moDC, or B cells from same donor were compared for their ability to detect MART$_{26\text{-}35}$-specific CD8⁺ T cells in blood samples.
(**E**) Gating strategies for detecting CD8⁺ T-cell responses in the co-culture system based on MART-1$_{26\text{-}35}$/HLA-A2 tetramers staining and intracellular Granzyme B staining.
(**F**) Frequency (%) of CTV⁽⁻⁾Granzyme B⁺ MART-1$_{26\text{-}35}$/HLA-A2 tetramer⁺ CD8⁺ T cells under the indicated conditions. Data are pooled from six (n=6) healthy donors in D and pooled from three (n=3) independent donors in F. Data are shown as means ± SEM. *p<0.05, **p<0.01 by one-way ANOVA with Tukey's multiple comparisons.

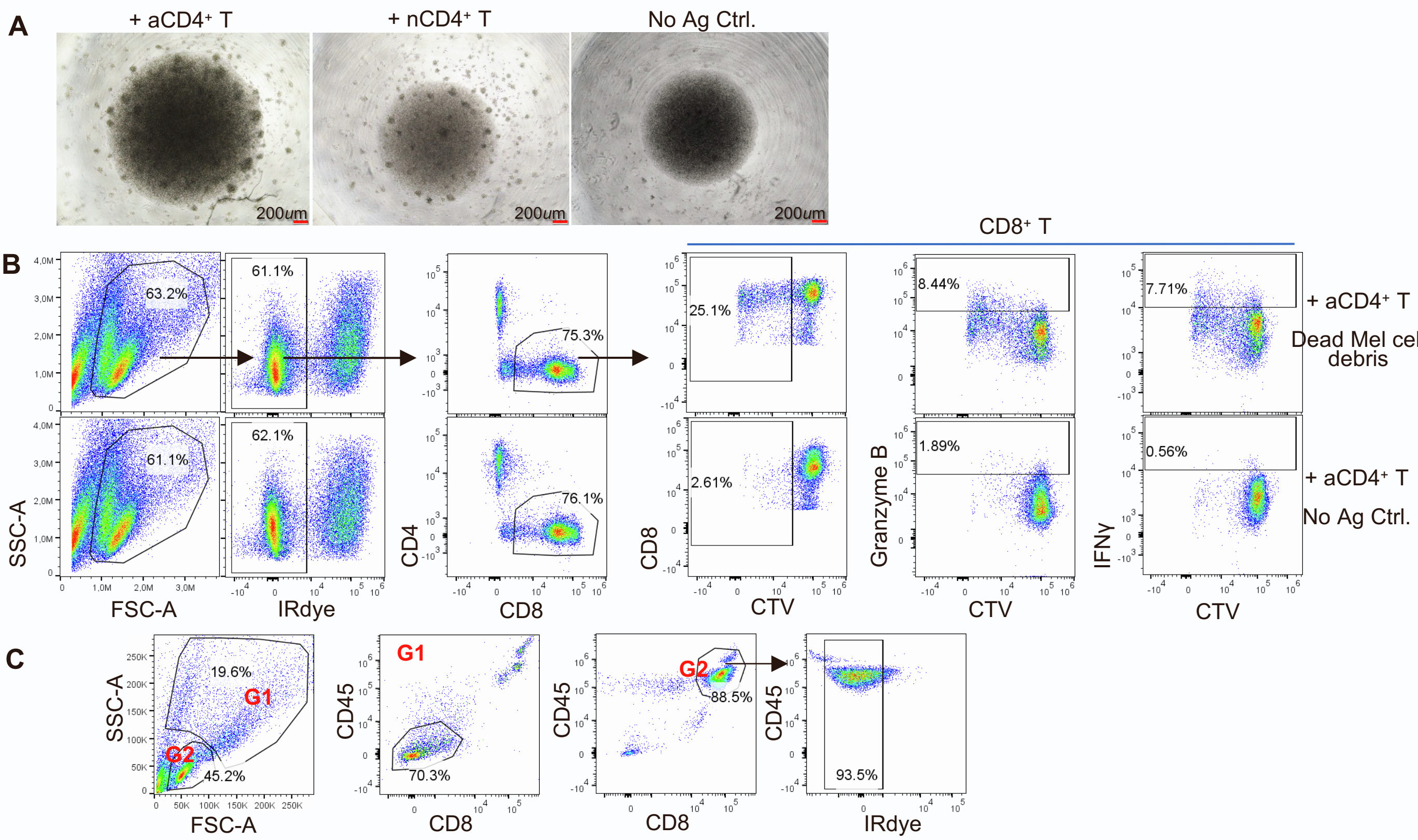


**Figure S8. "Licensed/helped" progenitor-derived DC prime CTL that exhibit enhanced anti-tumor function, related to Figure 7.**

CD8⁺ T cells were sorted from PBMC and co-cultured with progenitor-derived DC loaded with dead Mel526 cell debris in presence of activated (a)- or naïve (n)CD4⁺ T cells. Then CD8⁺ T-cell response was measured at day 6 after co-culture by flow cytometry or subjected to killing assay.

(**A**) Representative microscopic images depicting CD8⁺ T cells 6 days after co-culture.

(**B**) Gating strategies for detecting CD8⁺ T-cell responses in our *in vitro* cytotoxic T-cell priming system based on CTV dilution, and intracellular Granzyme B and IFNγ staining.

(**C**) Gating strategies for cells in suspension collected at the end of killing assay.

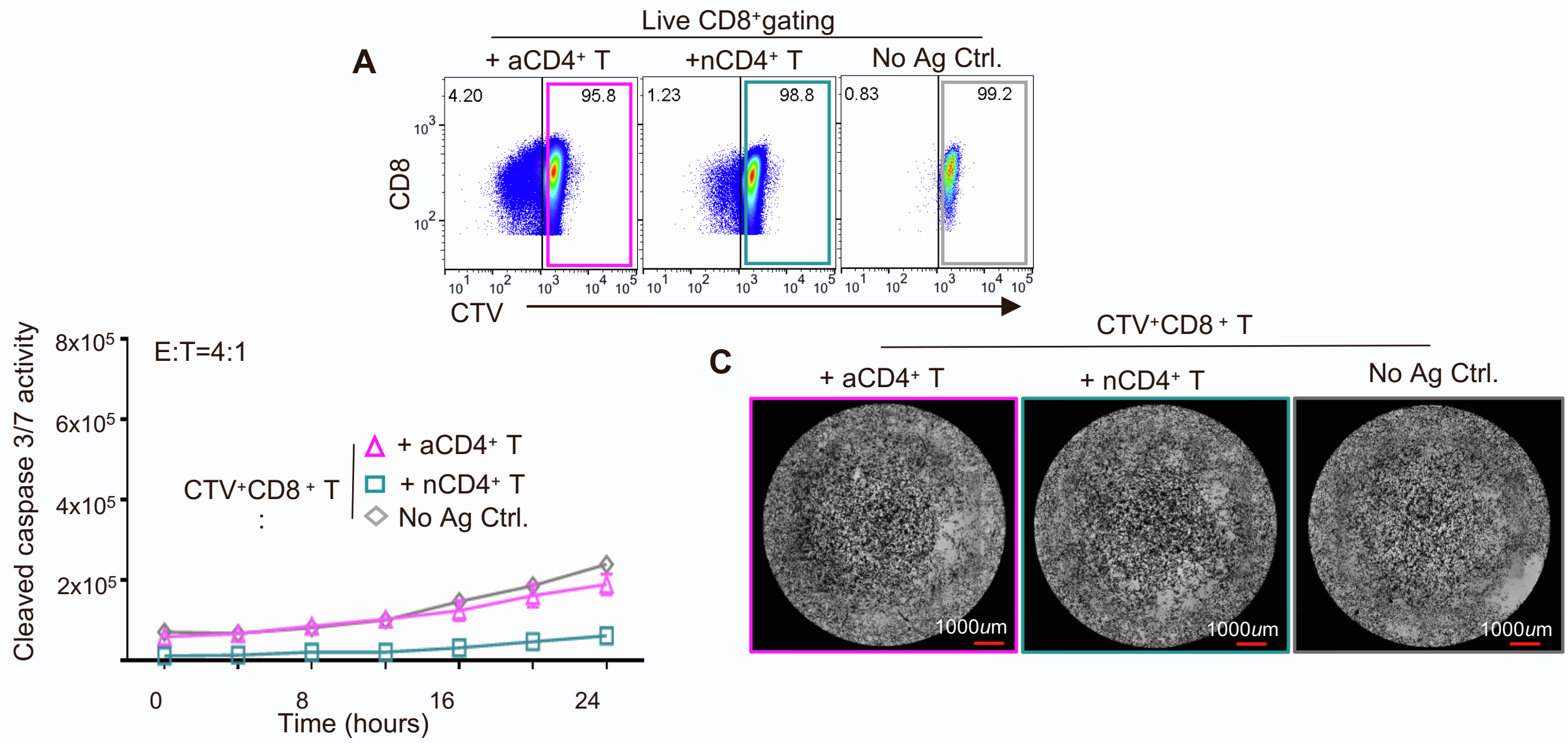


**Figure S9. Non-proliferative CTV⁺CD8⁺ T cells have limited tumor cell killing capacity, related to Figure 7.**

CD8⁺ T cells were sorted from PBMC and co-cultured with progenitor-derived DC loaded with dead Mel526 cell debris in presence of activated (a)- or naïve (n)CD4⁺ T cells. Then, killing assay was performed using non-proliferative (CTV⁺) CD8⁺ T cells purified from the priming system and Mel526 tumor cells at effector/target (E/T) ratio of 4/1, and analyzed by IncuCyte during 24h. At the end of the assay, remaining Mel526 cells in each well from all conditions were fixed and stained with crystal violet.

(**A**) Gating strategy for isolating CTV⁺CD8⁺ T cells from the CTL priming system. CTV⁺CD8⁺ T cells without encountering antigen were used as control. Purple, teal and grey boxes highlight isolated T-cell population.

(**B**) Cleaved caspase 3/7 activity in tumor cells during killing assay under indicated conditions.

(**C**) Representative CCD microscopic images depicting remaining Mel526 cells in the plate at 24h post co-culture under indicated conditions. Data are pooled from two (n=2) independent donors, are shown as means ± SD.

## SUPPLEMENTAL REFERENCES

1. Balan, S., Arnold-Schrauf, C., Abbas, A., Couespel, N., Savoret, J., Imperatore, F., Villani, A.C., Manh, T.P.V., Bhardwaj, N., and Dalod, M. (2018). Large-Scale Human Dendritic Cell Differentiation Revealing Notch-Dependent Lineage Bifurcation and Heterogeneity. Cell Reports 24, 1902-1915.e6. https://doi.org/10.1016/j.celrep.2018.07.033.

2. Lei, X., Khatri, I., Wit, T. de, Rink, I. de, Nieuwland, M., Kerkhoven, R., Eenennaam, H. van, Sun, C., Garg, A.D., Borst, J., et al. (2023). CD4+ helper T cells endow cDC1 with cancer-impeding functions in the human tumor micro-environment. Nature Communications 14. https://doi.org/10.1038/s41467-022-35615-5.
